# Safety and immunogenicity of investigational seasonal influenza hemagglutinin DNA vaccine followed by trivalent inactivated vaccine administered intradermally or intramuscularly in healthy adults: An open-label randomized phase 1 clinical trial

**DOI:** 10.1371/journal.pone.0222178

**Published:** 2019-09-18

**Authors:** Cristina Carter, Katherine V. Houser, Galina V. Yamshchikov, Abbie R. Bellamy, Jeanine May, Mary E. Enama, Uzma Sarwar, Brenda Larkin, Robert T. Bailer, Richard Koup, Grace L. Chen, Shital M. Patel, Patricia Winokur, Robert Belshe, Cornelia L. Dekker, Barney S. Graham, Julie E. Ledgerwood, the VRC 703 study team

**Affiliations:** 1 Vaccine Research Center, National Institute of Allergy and Infectious Diseases, National Institutes of Health, Bethesda, MD, United States of America; 2 The Emmes Corporation, Rockville, MD, United States of America; 3 Departments of Medicine and Molecular Virology and Microbiology, Baylor College of Medicine, Houston, TX, United States of America; 4 Department of Internal Medicine, Carver College of Medicine, University of Iowa, Iowa City, IA, United States of America; 5 Division of Infectious Diseases, Allergy and Immunology, Saint Louis University, St. Louis, MO, United States of America; 6 Department of Pediatrics (Infectious Diseases), Stanford University Medical Center, Stanford, CA, United States of America; IAVI, UNITED STATES

## Abstract

**Background:**

Seasonal influenza results in significant morbidity and mortality worldwide, but the currently licensed inactivated vaccines generally have low vaccine efficacies and could be improved. In this phase 1 clinical trial, we compared seasonal influenza vaccine regimens with different priming strategies, prime-boost intervals, and administration routes to determine the impact of these variables on the resulting antibody response.

**Methods:**

Between August 17, 2012 and January 25, 2013, four sites enrolled healthy adults 18–70 years of age. Subjects were randomized to receive one of the following vaccination regimens: trivalent hemagglutinin (HA) DNA prime followed by trivalent inactivated influenza vaccine (IIV3) boost with a 3.5 month interval (DNA-IIV3), IIV3 prime followed by IIV3 boost with a 10 month interval (IIV3-IIV3), or concurrent DNA and IIV3 prime followed by IIV3 boost with a 10 month interval (DNA/IIV3-IIV3). Each regimen was additionally stratified by an IIV3 administration route of either intramuscular (IM) or intradermal (ID). DNA vaccines were administered by a needle-free jet injector (Biojector). Study objectives included evaluating the safety and tolerability of each regimen and measuring the antibody response by hemagglutination inhibition (HAI).

**Results:**

Three hundred and sixteen subjects enrolled. Local reactogenicity was mild to moderate in severity, with higher frequencies recorded following DNA vaccine administered by Biojector compared to IIV3 by either route (p <0.02 for pain, swelling, and redness) and following IIV3 by ID route compared to IM route (p <0.001 for swelling and redness). Systemic reactogenicity was similar between regimens. Though no overall differences were observed between regimens, the highest titers post boost were observed in the DNA-IIV3 group by ID route and in the IIV3-IIV3 group by IM route.

**Conclusions:**

All vaccination regimens were found to be safe and tolerable. While there were no overall differences between regimens, the DNA-IIV3 group by ID route, and the IIV3-IIV3 group by IM route, showed higher responses compared to the other same-route regimens.

## Introduction

In the United States, seasonal influenza results in significant disease burden, with estimated ranges of 114,018–633,001 hospitalizations and 4,866–27,810 deaths each year [1]. Vulnerable populations, including older adults, are more susceptible to experiencing severe complications following influenza infection [1–3]. A recent survey spanning three consecutive influenza seasons found that between 54–70% of hospitalizations and 71–85% of deaths occur among adults above 65 years of age [1]. Even in young healthy adults where infection severity is typically milder, illness still results in substantial economic impact through absence from work and healthcare visits [4].

Vaccination is currently the most effective way to protect the population and lessen influenza disease burden. Licensed seasonal influenza vaccines include inactivated, live attenuated, and recombinant vaccines [5, 6]. The trivalent (IIV3) and quadrivalent (IIV4) inactivated influenza vaccines are updated annually to include hemagglutinin (HA) proteins from two circulating influenza A strains (H1N1 and H3N2) and one or two influenza B strains [5, 7, 8]. Inactivated vaccines can be administered by either the intramuscular (IM) route for individuals ≥6 months or by the intradermal (ID) route in adults 18–64 years old [5, 9]. While inactivated vaccines are the most widely administered, randomized controlled trials indicate inactivated vaccine efficacies typically range between only 62–75% for healthy adults, and just 43% for older adults [10–13]. Additional disadvantages to inactivated vaccines include a labor-intensive manufacturing process and, with the exception of recombinant and cell-based vaccines, most require embryonated eggs for production [14, 15].

DNA vaccines are an alternative vaccine platform that may improve immune responses and overcome the aforementioned disadvantages of inactivated influenza vaccines [16]. DNA vaccines consist of plasmids which can be easily modified through recombinant technology, and then rapidly developed and produced. While there are no licensed DNA vaccines to date, they do have an excellent safety profile, as demonstrated in multiple phase 1 clinical trials examining West Nile, SARS, influenza, and Zika [17–30]. Vaccination regimens consisting of a DNA prime followed by a heterologous inactivated boost have been previously explored as a strategy to improve immunogenicity for both seasonal and avian influenza subtypes. To date, such regimens have resulted in improved antibody responses for avian subtypes including H5 and H7 [17–20], but not for seasonal influenza subtypes [21, 22]. Timing of the prime-boost interval after a DNA prime has proven important for optimal antibody responses against novel antigens, including avian influenza subtypes. Antibody responses following DNA priming were higher after an inactivated boost with an interval of 3–6 months compared to an interval of only 1 month [19, 20]. However, the optimal prime-boost interval between a DNA prime and inactivated boost in seasonal influenza is unknown. Another question of interest for seasonal influenza is whether priming with a DNA and inactivated vaccine concurrently may result in an improved antibody response after an inactivated boost. Previously, a concurrent prime resulted in similar HAI titers to a DNA prime alone for an avian H7 subtype [17].

In this phase 1 clinical trial, we compared different priming strategies, prime-boost intervals, and routes of administration to determine whether these variables impacted the antibody response. Specifically, we evaluated whether priming with a DNA vaccine alone or concurrently with an inactivated vaccine would affect the antibody response following an inactivated boost in the same or subsequent influenza season. Additionally, we assessed the antibody response of the regimens in older adults to determine if a DNA prime would improve the vaccine response following an inactivated boost in this population.

## Materials and methods

### Ethics statement

**Fig 1:**
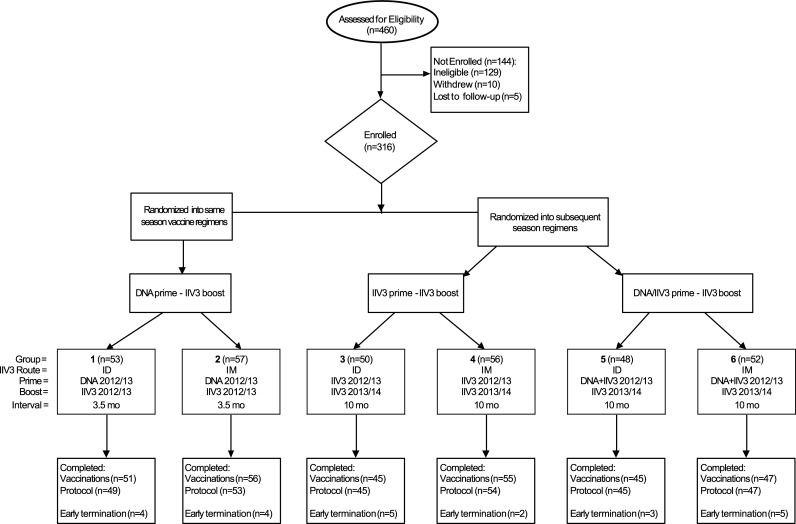
CONSORT diagram for VRC 703 clinical trial. Each group was stratified by age: younger adults (18–50 years) and older adults (51–70 years for IM or 51–64 for ID). All subjects who received at least one vaccination (n = 316) were analyzed for safety and reactogenicity. All subjects who completed the vaccination schedule (n = 299) were also analyzed for immunogenicity.

This study was a phase 1 open-label randomized clinical trial in healthy adults (clinicaltrials.gov, NCT01676402) to compare vaccination schedules over the same or subsequent influenza season (Fig 1). The study enrolled subjects at four sites, and the institutional review board (IRB) at each site approved the trial protocol (Baylor College of Medicine IRB approved the trial on July 3, 2012, University of Iowa on July 17, 2012, Saint Louis University on July 11, 2012, and Stanford University Medical Center on August 24, 2012). Subjects for this study were recruited by each site in accordance with their site IRB standard for recruitment practices. Enrollment for the trial occurred between August 17, 2012 and January 25, 2013, and the last subject visit occurred on April 25, 2014. Each subject signed a written informed consent prior to study participation. The trial registration on clinicaltrials.gov occurred on August 30, 2012, adhering to 42 CFR Part 11 requirements by occurring within 21 days of enrolling the first subject. The trial followed guidelines for conducting clinical research with human subjects from the US Department of Health and Human Services and was performed in accordance with 45 CFR Part 46, US Food and Drug Administration regulations for investigational products, and principles expressed in the Declaration of Helsinki.

### Study design and participants

Subjects were randomized in this clinical trial into groups through a staggered enrollment plan. Groups 1 and 2 (same influenza season regimens) consisted of a 2012/13 trivalent HA DNA prime and 2012/13 IIV3 boost administered by either ID or IM route with an approximate interval of 3.5 months (DNA-IIV3 regimen) (Fig 1, Table 1). The 3.5 month interval was chosen based on results from previous phase 1 trials investigating DNA vaccines of avian influenza subtypes which indicated that an improved antibody response is achieved with a 3–6 months interval between DNA prime and inactivated boost [19, 20]. The trial was designed to allow for the prime-boost interval to be shortened in the DNA prime groups (groups 1 and 2 only) when there was a local outbreak of influenza in the community, so that subjects would be able to receive a licensed product while the trial was ongoing. The interval was shortened for 10 subjects at a single site (4 subjects in group 1 and 6 subjects in group 2), and these subjects were included with the remainder of their groups in the immunogenicity analysis. Groups 3 to 6 (subsequent influenza season regimens) consisted of a prime of either 2012/13 IIV3 alone administered by either ID or IM route (groups 3 and 4) or with a concurrent HA DNA vaccine in the opposite arm (groups 5 and 6). These subjects were then boosted with 2013/14 IIV3 using the same route of administration as the IIV3 prime, with an approximate interval of 10 months (IIV3-IIV3 and DNA/IIV3-IIV3 regimens, respectively). Each group was stratified by age: younger adults (18–50 years) and older adults (51–70 years for IM or 51–64 years for ID). The age ranges in the older adults are consistent with the FDA approval for the IIV3 products [9, 31]. Other inclusion criteria included a BMI less than 40 and no prior receipt of the 2012/13 seasonal influenza vaccine. A complete listing of eligibility criteria is provided in the study protocol in the supplementary materials (S1 and S2 Protocols).

**Table 1:** Vaccine regimens and intervals of each group.

Group	IIV3 route^a^	Vaccine Regimen		Prime-boost Interval
		Prime	Boost	
**1**	ID	4mg HA DNA	2012/13 seasonal IIV3	3.5 months
**2**	IM			
**3**	ID	2012/13 seasonal IIV3	2013/14 seasonal IIV3	10 months
**4**	IM			
**5**	ID	HA DNA and 2012/13 seasonal IIV3	2013/14 seasonal IIV3	
**6**	IM			

^a^ Each administration of IIV3 by IM route contains 45mg total HA, while IIV3 by ID route contains 27mg total HA

Subjects self-reported local and systemic reactogenicity on a diary card for seven days after each vaccination, after receiving guidance on the grading of each symptom during the vaccination visit. All adverse events were recorded for 28 days following each vaccination. Serious adverse events (SAE) and influenza-like illnesses (ILI) were recorded for the entire duration of the trial.

### Vaccines

The investigational 2012/13 trivalent seasonal HA DNA (VRC-FLUDNA063-00-VP) consisted of identical amounts by weight of three DNA plasmids designed to closely match the recommended HA sequences contained within the licensed seasonal 2012/13 IIV3: VRC-9328 (A/California/04/2009, [A(H1N1)pdm09]), VRC-3027 (A/Victoria/361/2011, H3N2), and VRC-2722 (B/Wisconsin/1/2010) (Table 2). The trivalent HA DNA vaccine was manufactured at the NIAID Vaccine Research Center Vaccine Pilot Plant using Good Manufacturing Practices and formulated at 4mg/mL in PBS. The authors confirm that all ongoing and related trials for this product are registered.

**Table 2:** Influenza strains included in the vaccines.

Vaccine	A(H1N1)pdm09	H3N2	B
**2012/13 HA DNA**	A/California/04/2009	A/Victoria/361/2011	B/Wisconsin/1/2010
**2012/13 seasonal IIV3**	A/California/07/2009	A/Victoria/361/2011-like	B/Wisconsin/1/2010-like: B/Texas/6/2011
**2013/14 seasonal IIV3**	A/California/07/2009	A/Texas/50/2012	B/Massachusetts/2/2012

Fluzone (Sanofi Pasteur) was the licensed seasonal IIV3 used for IM (0.5mL, 45mg total HA) and ID (0.1mL, 27mg total HA) administration for both influenza seasons. The strains in the seasonal 2012/13 IIV3 included A/California/07/2009 [A(H1N1)pdm09], A/Victoria/361/2011-like virus (H3N2), and B/Wisconsin/1/2010-like: B/Texas/6/2011 (Table 2). The strains in the seasonal 2013/14 IIV3 included A/California/07/2009 [A(H1N1)pdm09], A/Texas/50/2012 (H3N2), and B/Massachusetts/2/2012 (Table 2).

All DNA vaccines were administered into the deltoid muscle using a needle-free jet injector, Biojector 2000 (Bioject; Tualatin, OR, USA). IIV3 was administered in the deltoid muscle either by ID route using a supplied short-needle microinjection system (groups 1, 3, and 5) or IM route using a needle and syringe (groups 2, 4, and 6).

### Randomization and masking

The study followed a staggered enrollment plan, with the same influenza season regimens enrolling first. In this first part, subjects were randomized with equal allocation to groups 1 or 2. Randomization was then opened to the subsequent influenza season regimens with equal allocation to groups 3 to 6. The randomization was staggered so that all subjects could receive the seasonal 2012/13 IIV3 at approximately the same time, soon after it became available. Subjects 65–70 years of age were enrolled only into groups 2, 4, and 6 to ensure IIV3 administration by the IM route. The randomization sequence was generated by the trial statistician using permuted blocked randomization with randomly selected block sizes (2 or 4 in the first part, 4 or 8 in the second part), stratified by age and site. Upon enrollment, each subject’s randomized assignment was displayed in the electronic data entry system and vaccinations were administered open-label.

### Immunogenicity assays

Blood samples were collected prior to each vaccination, 3 weeks after both the prime and boost vaccinations, and 6 months after the boost. Subject sera were tested for antibody response by hemagglutination inhibition (HAI). HAI assays were completed at Southern Research, Inc. (Birmingham, AL) using previously described validated methods [21]. Briefly, the HAI assays were performed in V-bottom 96-well plates with four HA units of virus and 0.5% turkey red blood cells.

### Statistical analysis

The primary objectives of the study were (1) to assess the safety and tolerability of each vaccination schedule, including concurrent prime of DNA and IIV3 in different arms, and (2) to compare the antibody responses by HAI at 3 weeks after IIV3 administration between the DNA-IIV3 regimen and IIV3 alone. The primary outcomes of safety and tolerability were measured by a combination of solicited and unsolicited reactogenicity for 7 days following each vaccination, and the occurrence of adverse events, serious adverse events, and influenza-like illnesses for the duration of the trial. Solicited reactogenicity included pain/tenderness, swelling, redness, malaise, myalgia, headache, chills, nausea, and temperature. Secondary objectives included evaluating HAI antibody titers in all groups at 3 weeks after each study injection. We analyzed all outcomes on the basis of the intention-to-treat principle. All subjects who received at least one vaccination were analyzed for safety and reactogenicity. All subjects who completed the vaccination schedule were also analyzed for immunogenicity. This trial was designed as a descriptive study with a planned enrollment of 55 subjects per group. The study was not designed to test a formal null hypothesis for superiority or non-inferiority. With the planned sample size, the study was likely to detect event occurring with high frequency (>90% chance to observe at least one safety event if the true rate is at least 0.05 (1 of 20), but would be unlikely (>90% chance of observing no events) occurring at a rate less than 0.002 (1 of 500). This minimum group size allowed for 80% power with significance level 5% to detect a 28% difference in seroconversion rates between two groups if the proportion of responders in the reference group was 40%.

The seroconversion rate for HAI was determined per the FDA definition as either a baseline (Day 0) HAI titer <1:10 and a post boost HAI titer ≥1:40, or a baseline HAI titer ≥1:10 and a minimum four-fold rise from baseline [32]. The magnitude of immune response was calculated as the geometric mean titer (GMT). Comparisons were made between overall groups and between age groups within each overall group. A Fisher’s exact test was used for comparisons of seroconversion rates and the t-test was used to compare magnitudes of log-transformed HAI titers. The primary analysis adjusted for the effect of site using the logistic (seroconversion outcome) and analysis of variance (log-transformed titer response outcome). Chi-square tests were used to compare the distribution of solicited symptom severity between groups. Statistical significance was considered at a level of alpha = 0.05. Adjustment for multiple comparisons was not performed as statistical inferences in this small phase 1 clinical trial are hypothesis-generating, and not confirming. Statistical analyses were performed in SAS 9.3 (SAS Institute, Cary, North Carolina).

## Results

### Study population and vaccine safety

A total of 316 subjects were enrolled in the study between August 17, 2012 and January 25, 2013 (Fig 1). The group demographics are displayed in Table 3. Overall, there were 125 (39.6%) males and 191 (60.4%) females, with an average age of 41 years. Study vaccinations were completed by 299 subjects (94.6%), and 293 subjects (92.7%) completed the protocol. Reasons for the 23 subjects who did not complete the protocol include: ineligible due to previous receipt of 2012/13 IIV3, failure to disclose hepatitis C infection, withdrawal after diagnosis of pancreatic cancer (unrelated SAE), death by myocardial infarction (unrelated SAE), unrelated ongoing fatigue, and subjects moved or were lost to follow up.

**Table 3:** Baseline demographics of participants.

Characteristics	Treatment Group						Overall (n = 316)
	Group 1 DNA/IIV3 ID route^a^ (n = 53)	Group 2 DNA/IIV3 IM route (n = 57)	Group 3 IIV3/IIV3 ID route (n = 50)	Group 4 IIV3/IIV3 IM route (n = 56)	Group 5 DNA-IIV3/IIV3 ID route (n = 48)	Group 6 DNA-IIV3/IIV3 IM route (n = 52)	
**Sex - no. (%)**							
**Male**	21 (39.6)	22 (38.6)	19 (38.0)	20 (35.7)	20 (41.7)	23 (44.2)	125 (39.6)
**Female**	32 (60.4)	35 (61.4)	31 (62.0)	36 (64.3)	28 (58.3)	29 (55.8)	191 (60.4)
**Age - years**^**b**^							
**Mean (SD)**	39.4 (14)	42.4 (17)	41.9 (14)	41.9 (16)	40.2 (15)	41.2 (15)	41.2 (15)
**Range**	[20, 63]	[20, 70]	[19, 62]	[18, 69]	[18,63]	[18, 64]	[18, 70]
**Race - no. (%)**							
**American Indian/Alaskan Native**	0 (0.0)	1 (1.8)	0 (0.0)	0 (0.0)	0 (0.0)	0 (0.0)	1 (0.3)
**Asian**	3 (5.7)	4 (7.0)	5 (10.0)	2 (3.6)	5 (10.4)	4 (7.7)	23 (7.3)
**Black or African American**	2 (3.8)	4 (7.0)	6 (12.0)	1 (1.8)	5 (10.4)	6 (11.5)	24 (7.6)
**Native Hawaiian/Pacific Islander**	0 (0.0)	0 (0.0)	0 (0.0)	0 (0.0)	0 (0.0)	0 (0.0)	0 (0.0)
**White**	44 (83.0)	47 (82.5)	38 (76.0)	47 (83.9)	35 (72.9)	41 (78.8)	252 (79.7)
**Multiracial**	4 (7.5)	0 (0.0)	1 (2.0)	5 (8.9)	3 (6.3)	1 (1.9)	14 (4.4)
**Other/Unknown**	0 (0.0)	1 (1.8)	0 (0.0)	1 (1.8)	0 (0.0)	0 (0.0)	2 (0.6)
**Ethnicity - no. (%)**							
**Non-Hispanic/Latino**	50 (94.3)	52 (91.2)	48 (96.0)	50 (89.3)	47 (97.9)	50 (96.2)	297 (94.0)
**Hispanic/Latino**	3 (5.7)	5 (8.8)	2 (4.0)	6 (10.7)	1 (2.1)	2 (3.8)	19 (6.0)
**Body Mass Index (BMI)**^**c**^							
**Mean (SD)**	26.8 (4.9)	25.9 (4.4)	25.8 (5.0)	26.3 (5.1)	26.4 (4.9)	26.4 (4.3)	26.2 (4.7)
**Range**	[17.2, 38.3]	[19.1, 35.1]	[18.3, 38.3]	[18.6, 38.5]	[18.5, 38.2]	[19.3, 35.6]	[17.2, 38.5]
**Influenza vaccinations in the previous 5 years - no. (%)**							
**>5 times**	4 (7.5)	11 (19.3)	10 (20.0)	8 (14.3)	6 (12.5)	10 (19.2)	49 (15.5)
**3–5 times**	22 (41.5)	24 (42.1)	18 (3.0)	21 (37.5)	26 (54.2)	18 (34.6)	129 (40.8)
**1–2 times**	19 (35.8)	15 (26.3)	9 (18.0)	15 (26.8)	10 (20.8)	10 (19.2)	78 (24.7)
**0 times**	8 (15.1)	7 (12.3)	13 (26.0)	12 (21.4)	6 (12.5)	14 (26.9)	60 (19.0)

^a^Route only pertains to IIV3 vaccine. DNA vaccines were administered by Biojector.

^b^Age represents age at enrollment (date of prime vaccination).

^c^Height and weight (used for BMI) measured at Day 0.

All local reactogenicity was mild to moderate in severity (Table 4). DNA prime administered by Biojector (groups 1, 2, 5 and 6) resulted in significantly higher frequencies of pain and tenderness, redness, and swelling compared to IIV3 by either route (groups 3, 4, 5 and 6) (p <0.02 for all symptoms). IIV3 administered by the ID route resulted in significantly higher frequencies of redness and swelling compared to IIV3 administered by IM route (p <0.001 for both redness and swelling). There was one report of a severe headache following concurrent DNA/IIV3 prime when IIV3 was administered by the ID route and one report of a fever of 102.3°F following IIV3 boost by the ID route that coincided with reports of ILIs in the subjects. One subject reported a combination of severe malaise, headache, and nausea following IIV3 boost by the ID route with no related illness. All of these events resolved without sequelae. Otherwise, systemic reactogenicity was similar among all groups (S1 Table).

**Table 4:** Summary of solicited local reactogenicity after prime and boost vaccination.

Symptoms Intensity	Group 1 DNA/IIV3 ID (N = 53/N = 51)^a^	Group 2 DNA/IIV3 IM (N = 57/N = 56)	Group 3 IIV3-IIV3 ID (N = 50/N = 45)	Group 4 IIV3-IIV3 IM (N = 56/N = 55)	Group 5 DNA/IIV3-IIV3 ID (N = 48/N = 45)^b^		Group 6 DNA/IIV3-IIV3 IM (N = 52/N = 47)^b^	
					DNA	IIV3	DNA	IIV3
**Prime Vaccination**								
**PAIN/TENDERNESS**								
**None**	4 (7.5%)	10 (17.5%)	19 (38.0%)	26 (46.4%)	7 (14.6%)	21 (43.8%)	9 (17.3%)	26 (50.0%)
**Mild**	47 (88.7%)	41 (71.9%)	31 (62.0%)	29 (51.8%)	38 (79.2%)	25 (52.1%)	38 (73.1%)	25 (48.1%)
**Moderate**	2 (3.8%)	6 (10.5%)	0 (0.0%)	1 (1.8%)	3 (6.3%)	2 (4.2%)	5 (9.6%)	1 (1.9%)
**SWELLING**								
**None**	49 (92.5%)	51 (89.5%)	32 (64.0%)	56 (100.0%)	42 (87.5%)	28 (58.3%)	47 (90.4%)	52 (100.0%)
**Mild**	4 (7.5%)	5 (8.8%)	16 (32.0%)	0 (0.0%)	4 (8.3%)	18 (37.5%)	5 (9.6%)	0 (0.0%)
**Moderate**	0 (0.0%)	1 (1.8%)	2 (4.0%)	0 (0.0%)	2 (4.2%)	2 (4.2%)	0 (0.0%)	0 (0.0%)
**REDNESS**								
**None**	48 (90.6%)	53 (93.0%)	15 (30.0%)	54 (96.4%)	41 (85.4%)	26 (54.2%)	44 (84.6%)	52 (100.0%)
**Mild**	5 (9.4%)	4 (7.0%)	26 (52.0%)	2 (3.6%)	6 (12.5%)	18 (37.5%)	7 (13.5%)	0 (0.0%)
**Moderate**	0 (0.0%)	0 (0.0%)	9 (18.0%)	0 (0.0%)	1 (2.1%)	4 (8.3%)	1 (1.9%)	0 (0.0%)
**ANY LOCAL SYMPTOM**^**c**^								
**None**	3 (5.7%)	10 (17.5%)	6 (12.0%)	26 (46.4%)	2 (4.2%)		3 (5.8%)	
**Mild**	48 (90.6%)	41 (71.9%)	34 (68.0%)	29 (51.8%)	37 (77.1%)		43 (82.7%)	
**Moderate**	2 (3.8%)	6 (10.5%)	10 (20.0%)	1 (1.8%)	9 (18.8%)		6 (11.5%)	
**Boost Vaccination**								
**PAIN/TENDERNESS**								
**None**	20 (39.2%)	26 (46.4%)	18 (40.0%)	30 (54.5%)	21 (46.7%)		28 (59.6%)	
**Mild**	31 (60.8%)	30 (53.6%)	26 (57.8%)	25 (45.5%)	23 (51.1%)		19 (40.4%)	
**Moderate**	0 (0.0%)	0 (0.0%)	1 (2.2%)	0 (0.0%)	1 (2.2%)		0 (0.0%)	
**SWELLING**								
**None**	39 (76.5%)	55 (98.2%)	31 (68.9%)	54 (98.2%)	33 (73.3%)		47 (100.0%)	
**Mild**	11 (21.6%)	1 (1.8%)	11 (24.4%)	1 (1.8%)	8 (17.8%)		0 (0.0%)	
**Moderate**	1 (2.0%)	0 (0.0%)	0 (0.0%)	0 (0.0%)	0 (0.0%)		0 (0.0%)	
**REDNESS**								
**None**	17 (33.3%)	55 (98.2%)	17 (37.8%)	53 (96.4%)	23 (51.1%)		46 (97.9%)	
**Mild**	23 (45.1%)	1 (1.8%)	16 (35.6%)	2 (3.6%)	16 (35.6%)		1 (2.1%)	
**Moderate**	11 (21.6%)	0 (0.0%)	12 (26.7%)	0 (0.0%)	6 (13.3%)		0 (0.0%)	
**ANY LOCAL SYMPTOM**^**c**^								
**None**	8 (15.7%)	26 (46.4%)	6 (13.3%)	29 (52.7%)	12 (26.7%)		28 (59.6%)	
**Mild**	32 (62.7%)	30 (53.6%)	26 (57.8%)	26 (47.3%)	25 (55.6%)		19 (40.4%)	
**Moderate**	11 (21.6%)	0 (0.0%)	13 (28.9%)	0 (0.0%)	8 (17.8%)		0 (0.0%)	

For participants reporting a symptom on multiple days, the symptom is counted once at the maximum severity.

^a^(N = # subjects receiving prime vaccination/N = # subjects receiving boost vaccination)

^b^Groups 5 and 6 received a prime vaccination that included 2012/13 IIV3 administered in one arm and HA DNA vaccine in the opposite arm, allowing for the collection of distinct solicited local reactogenicity

^c^Any Local Symptom displays the summation of the individually listed solicited local reactogenicity

There were eight SAEs reported during the trial, and all were evaluated as unrelated to the study vaccines. There were 15 reported cases of ILI during the trial; however, no testing was conducted to distinguish influenza infection from other etiologies.

### Preexisting antibody responses were present in all groups

Prior to vaccination, all groups had preexisting antibody titers by HAI to the influenza vaccine antigens included in the analysis (S2 Table). The proportion of subjects with baseline titers of ≥1:40 were similar across groups with the following ranges: 35–45% for A/California/07/2009 [A(H1N1)pdm09], 16–26% for A/Victoria/361/2011 (H3N2), and 40–47% for A/Texas/50/2012 (H3N2). Preexisting responses to influenza B strains were lower: 14–26% for both B/Wisconsin/1/2010 and B/Texas/6/2011, and 8–21% to B/Massachusetts/2/2012. Individual baseline titers were not found to have a significant impact on the response to the vaccine regimens in the trial.

### DNA priming resulted in higher antibody GMTs and response rates following IIV3 boost compared to a single administration of IIV3 by the ID route

The primary immunogenicity objective was to compare whether the addition of DNA priming would affect the magnitude of the antibody response or seroconversion rates of a single administration of IIV3. To achieve this, two groups of subjects received a DNA prime followed by an IIV3 boost (DNA-IIV3 regimen) approximately 3.5 months later by either ID route (group 1) or IM route (group 2) (Table 1). Low antibody GMTs and seroconversion rates were observed following DNA prime prior to IIV3 administration (S2 and S3 Tables), consistent with previous studies demonstrating that single administrations of DNA vaccines encoding soluble protein antigens do not significantly boost preexisting antibody responses [23]. To compare the antibody response following completion of the DNA-IIV3 regimen to a single administration of IIV3, we evaluated the antibody response after the IIV3 boost in groups 1 and 2 to the antibody response at 3 weeks following the IIV3 prime vaccination in groups 3 (IIV3 by ID route) and 4 (IIV3 by IM route) (Table 1).

**Fig 2:**
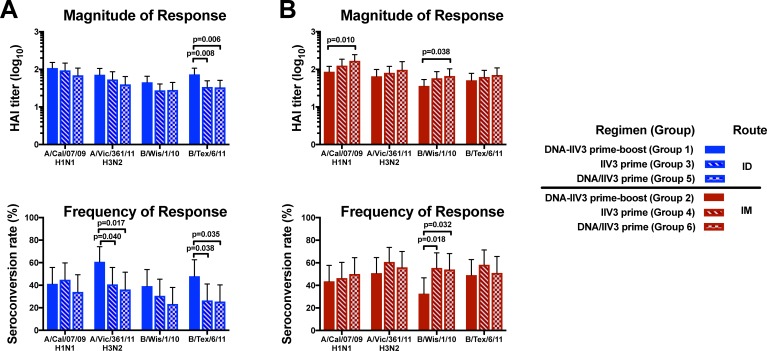
Magnitude and frequency of antibody responses at three weeks after IIV3 administration for DNA-IIV3 prime-boost, IIV3 prime, or concurrent DNA/IIV3 prime. The antibody responses were measured for the (A) ID route and (B) IM route by hemagglutination inhibition assay (HAI) and are displayed by group geometric mean titers (GMT) or group seroconversion rates for all 2012/13 vaccine strains, with error bars indicating the 95% CI. Comparisons were made between vaccine regimens for each route. Displayed p values for seroconversion rates were calculated based on Fisher’s Exact test, while GMT comparisons were based on pairwise T-test.

Following IIV3 administration via the ID route, the DNA-IIV3 regimen displayed a significant increase compared to a single administration of IIV3 in both the magnitude of the antibody response and the seroconversion rates for B/Texas/6/11 (p values of 0.008 and 0.038, respectively), and the seroconversion rates for A/Victoria/361/11 (p value of 0.040) (groups 1 and 3, Fig 2A). The DNA-IIV3 regimen by the ID route also trended towards increased antibody responses compared to a single administration of IIV3 for all strains tested, although these differences were not statistically significant (groups 1 and 3, Fig 2A).

For regimens administering IIV3 by the IM route, a single administration of IIV3 displayed a significantly higher seroconversion rate compared to the DNA-IIV3 regimen for B/Wisconsin/1/10 (p = 0.018) (groups 2 and 4, Fig 2B). A single administration of IIV3 also trended towards higher antibody responses compared to DNA-IIV3 for all other strains tested, although these differences were not statistically significant (groups 2 and 4, Fig 2B).

### Concurrent administration of DNA and IIV3 did not result in improved antibody responses compared to IIV3 alone

We also included a regimen in the trial with a prime vaccination that included a concurrent administration of DNA and IIV3 (DNA/IIV3) to assess whether simultaneous vaccination with DNA and IIV3 in separate arms could improve antibody responses compared with IIV3 (Table 1). This would allow for a single annual vaccination visit rather than a prime-boost regimen requiring two separate visits. Similar to the other vaccine regimens, the IIV3 was administered by either ID route (group 5) or IM route (group 6). Our analysis at 3 weeks following the prime vaccination suggest that administering DNA and IIV3 concurrently did not result in a consistently improved immune response over a single administration of IIV3 by either route (Fig 2).

### The DNA-IIV3 regimen by ID route and the IIV3-IIV3 regimen by IM route obtained the highest antibody responses after boost

**Fig 3:**
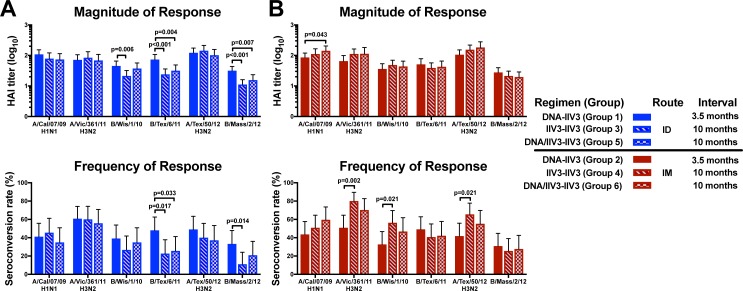
Vaccine regimen comparison of magnitude and frequency of antibody response at 3 weeks post boost. The GMT and seroconversion rates are shown for antibody responses measured by HAI for all 2012/13 and 2013/14 vaccine strains for the (A) ID route and (B) IM route, with error bars indicating the 95% CI. Comparisons were made between vaccine regimens for each route. Displayed p values for seroconversion rates were calculated based on Fisher’s Exact test, while GMT comparisons were based on pairwise T-test.

While DNA priming provides an optimal antibody response with a prime-boost interval between 3–6 months [20], inactivated influenza vaccines are typically given on an annual basis. In order to compare the prime-boost responses of the DNA-IIV3 regimen to a more traditional IIV3 prime-boost schedule, the groups that received a prime administration of IIV3 (groups 3–6) were boosted with a second IIV3 during the subsequent 2013/14 influenza season, with an interval of approximately 10 months (Table 1). For each group, the IIV3 boost was administered through the same route as the initial IIV3. We then compared the antibody responses by HAI for the three completed vaccine regimens (DNA-IIV3, IIV3-IIV3, and DNA/IIV3-IIV3) at 3 weeks post boost (Fig 3).

The DNA-IIV3 regimen by the ID route resulted in significantly higher antibody titers and seroconversion rates compared to the other two vaccine regimens against the influenza B strains: B/Texas/6/11 (overall p values of <0.001 and 0.020, respectively) and B/Massachusetts/2/12 (overall p values of <0.001and 0.033, respectively), and a significantly higher magnitude of antibody response for B/Wisconsin/1/10 (overall p = 0.020) (Fig 3A). The magnitude of antibody response continued to be significantly increased at 6 months post boost for B/Texas/6/11 and B/Massachusetts/2/12 (overall p = 0.012 for both) (S3 Table). No differences were noted in the antibody responses for the influenza A strains between regimens.

Among the regimens where IIV3 was administered by the IM route, the IIV3-IIV3 regimen showed higher seroconversion rates compared to the DNA-IIV3 regimen for the H3N2 strains A/Victoria/361/11 (p = 0.002) and A/Texas/50/12 (p = 0.021), as well as B/Wisconsin/1/10 (p = 0.021) (Fig 3B). Therefore, the DNA-IIV3 regimen trended towards higher antibody responses in influenza B strains among the ID regimens and IIV3-IIV3 trended towards higher seroconversion after boost among the regimens where IIV3 was administered IM.

### Administration of IIV3 by ID route was not always dose-sparing

Antibody responses post boost were also evaluated by comparing the ID route to the IM route within each regimen. The ID route is intended to be dose-sparing by providing antibody responses comparable to IM administration while requiring less antigen per dose [33–35]. No consistent improvement in antibody responses was detected between routes for all regimens. However, when significant strain-specific differences were observed, the higher responses were found following IM administration (S1 Fig), indicating that the ID route was not always dose-sparing in this trial. These differences diminished by 6 months post boost (S2 and S3 Tables). Interestingly, in regimens with a DNA only prime (groups 1 and 2), there were no significant differences between IM and ID groups, suggesting that a DNA only prime may augment responses to the low-dose antigen of an IIV3 ID boost.

### Neither DNA prime nor ID route improved the vaccine response in older adults

Since IIV3 can be less effective in the older population, we also compared the antibody responses between the younger (18–50 years) and older (51+ years) age groups within each vaccination regimen. In general, there were significantly increased antibody titers and seroconversion rates in the younger population when compared to the older population, at all time points measured (S4 and S5 Tables). These results are consistent with previous studies using DNA vaccines to prime subsequent boosting with conventional inactivated influenza vaccines [22].

**Fig 4:**
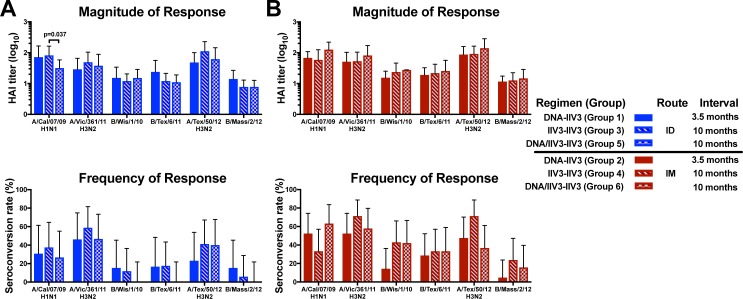
Magnitude and frequency of antibody response at 3 weeks post boost in older adults. The GMT and seroconversion rates are shown for antibody responses measured by HAI for all 2012/13 and 2013/14 vaccine strains in older adults following (A) ID route and (B) IM route, with error bars indicating the 95% CI. Comparisons were made between vaccine regimens for each route. Displayed p values for seroconversion rates were calculated based on Fisher’s Exact test, while GMT comparisons were based on pairwise T-test.

We were also interested in determining whether DNA priming would improve the antibody response in older adults. For this analysis, we examined the antibody responses of the three vaccine regimens in the older adults at time points throughout the clinical trial (Fig 4, S4 and S5 Tables). There was no overall improvement at any time point, suggesting that none of the tested regimens resulted in consistently improved antibody responses in older adults. Therefore, neither the addition of a DNA prime with a shorter prime-boost interval nor a concurrent DNA/IIV3 prime with a longer prime-boost interval improved the antibody response in the older population.

Antibody responses in the older population were also compared by route of administration at 3 weeks post boost. We did not observe any effect of age on the route comparison due to the different FDA-approved age ranges for IIV3 administration routes in the older adults. Generally, both the magnitude of the antibody response and the seroconversion rates were similar between administration routes for each regimen (S2 Fig). Similar to our observations for the entire study population, when differences were observed, IM administration resulted in higher responses compared to ID administration, suggesting that administration of IIV3 by ID route was not always dose-sparing in these subjects unless they were primed with DNA.

## Discussion

In this phase 1 open-label randomized clinical trial, we assessed the safety and tolerability of multiple seasonal influenza vaccination regimens in healthy adults. Local reactogenicity was mild to moderate in severity, with higher frequencies of pain/tenderness, swelling, and redness following DNA vaccination by Biojector compared to IIV3 by either administration route, and higher frequencies of redness and swelling following IIV3 administration by the ID route compared to the IM route. These findings are consistent with previous clinical trials [21, 26, 36]. Overall, all vaccine regimens were found to be safe and well tolerated. We also found that variables including priming vaccines, prime-boost intervals, and administration routes did not consistently impact the antibody response following an IIV3 boost in healthy adults who had substantial levels of preexisting immunity to the vaccine antigens used in this study. A limitation to this study is the small trial size, which is typical of phase 1 clinical trials.

While the antibody responses between the different vaccination regimens did not differ overall, we did observe several trends that remained consistent throughout the trial. After each IIV3 administration we observed the highest antibody responses in the DNA-IIV3 group when IIV3 was administered by the ID route (group 1), and the IIV3-IIV3 group when IIV3 was administered by the IM route (group 4). The observed trend in group 1 may indicate that ID is the preferred route for IIV3 administration following a DNA prime, but only when paired with an optimal prime-boost interval of 3–6 months. If the boost occurs outside of this window, our data suggest that an inactivated prime delivered by IM may provide a superior response. Interestingly, a difference in antibody response was not observed between the concurrent DNA/IIV3-IIV3 regimen by ID route (group 5) or IM route (group 6), perhaps due to immune interference or the prolonged prime-boost interval. Even when compared to a single IIV3, concurrent administrations of DNA and IIV3 did not improve the antibody response, confirming that a DNA prime must be paired with an appropriately timed boost to achieve optimal immunogenicity. This finding suggests that DNA priming establishes a distinct population of responsive memory B cells that are not induced or sustained in the same way by immunization with protein as in the IIV3.

The subjects in group 1 who received the DNA-IIV3 regimen by ID route had significantly higher antibody responses to the influenza B strains B/Wisconsin/1/2010 and B/Texas/6/2011 compared to the other vaccine regimens administered by the ID route. These subjects also exhibited an increased response to the 2013/14 strain B/Massachusetts/2/12, even though this strain was not included in either 2012/13 vaccine product. Since all of the B strains in this trial are of the Yamagata lineage, one possible explanation is that DNA priming allowed for an increased breadth of antibody response against drifted influenza B strains. In a previous trial examining seasonal influenza vaccination with a DNA prime followed by an IIV3 boost, a significant antibody increase was not observed for the influenza B strains tested [22]. However, that trial included a DNA prime with an influenza B strain of the Victoria lineage while the IIV3 boost contained a B strain from the Yamagata lineage. Therefore, DNA priming may induce memory B cells against influenza B that are strain-specific and are not boosted well by antigenically varied vaccines.

In this trial, we did not observe a regimen that was superior for the older population. DNA vaccines have been a strategy of interest in older adults because they are believed to not only expand memory B cell populations, but also induce a variety of T cell responses [37–39]. While we did not directly analyze T cell responses in this study, the regimens that included a DNA prime did not result in significantly improved antibody responses in the older adults. Multiple studies comparing ID and IM routes of administration for inactivated influenza vaccines in older adults have shown comparable immunogenicity outcomes [36, 40, 41]; whereas we observed that the ID route for IIV3 was not always dose-sparing in this trial. However, this phase 1 trial was small in size and not designed with dose-escalations within the ID or IM routes, and therefore not powered for a more conclusive analysis of this observation. Currently, high-dose IIV3 via the IM route (0.5mL, 180 μg total HA) is recommended for older adults because it has been shown to improve immunogenicity via both ID and IM routes [41–43]. Future studies might consider incorporating DNA priming with high-dose IIV3 boosting, and include an assessment of T cell responses, to further evaluate whether DNA priming could improve immunogenicity in the older population.

Since this clinical trial began in 2012 there have been several advances for both DNA and influenza vaccines. Additional clinical trials have been published for DNA vaccines, providing further proof of the safety and immunogenicity of this vaccine platform [24, 30], and preclinical studies have begun evaluating novel delivery routes including microneedle patches [44, 45]. Several new influenza vaccines have also become licensed, including a quadrivalent version of the seasonal inactivated vaccine [5, 7]. This clinical trial began before the quadrivalent vaccine became commercially available, and we were therefore unable to include the IIV4 in our evaluation. However, we predict the IIV4 would result in similar trends as we observed with the IIV3, while possibly providing increased protection against more antigenically drifted influenza B strains.

In summary, we found that all vaccine regimens in the study were safe and well tolerated. We did not observe any consistent improvement in antibody responses by varying the priming vaccination, prime-boost interval, or administration route for IIV3 in this phase 1 study in healthy adults. In the older population, the addition of a DNA prime did not improve the antibody response following an IIV3 boost, but future studies may be warranted to assess responses if paired with a high-dose IIV3.

## Supporting information

S1 CONSORT Checklist CONSORT checklist for VRC 703 clinical trial. (DOC)

S1 Protocol VRC 703 clinical trial protocol. Protocol version before enrollment.
(PDF)

S2 Protocol VRC 703 clinical trial protocol. Final protocol version.
(PDF)

S1 Table Summary of solicited systemic reactogenicity after prime and boost vaccination. (PDF)

S2 Table Seroconversion rates for study groups as measured by HAI. (PDF)

S3 Table Magnitude of antibody responses for study groups as measured by HAI. (PDF)

S4 Table Magnitude of antibody responses for age subgroups as measured by HAI. (PDF)

S5 Table Seroconversion rates for age subgroups as measured by HAI. (PDF)

S1 Fig Route comparison of magnitude and frequency of antibody response in all subjects post boost. (A) GMT and (B) seroconversion rates are shown for antibody responses measured by HAI at 3 weeks post boost for all 2012/13 and 2013/14 vaccine strains, with error bars indicating the 95% CI. For each vaccination regimen results are displayed for both the ID (blue) and IM (red) routes. Comparisons were made between administration routes for each vaccine regimen. Displayed p values for seroconversion rates were calculated based on Fisher’s Exact test, while GMT comparisons were based on pairwise T-test.
(PDF)

S2 Fig Route comparison of magnitude and frequency of antibody response in the older adult subgroups post boost. (A) GMT and (B) seroconversion rates are shown for antibody responses measured by HAI at 3 weeks post boost for all 2012/13 and 2013/14 vaccine strains, with error bars indicating the 95% CI. For each vaccination regimen results are displayed for both the ID (blue) and IM (red) routes. Comparisons were made between administration routes for each vaccine regimen. Displayed p values for seroconversion rates were calculated based on Fisher’s Exact test, while GMT comparisons were based on pairwise T-test.
(PDF)

S1 Data Raw data from VRC 703 clinical trial. (XLSX)
