# Supplementary material for: Safety and immunogenicity of investigational seasonal influenza hemagglutinin DNA vaccine followed by trivalent inactivated vaccine administered intradermally or intramuscularly in healthy adults: An open-label randomized phase 1 clinical trial
Source: PLoS One. 2019 Sep 18;14(9):e0222178. doi: 10.1371/journal.pone.0222178 (PMC6750650; doi:10.1371/journal.pone.0222178)
Supplement: S2 Protocol — Final protocol version. (PDF) [file pone.0222178.s003.pdf]

An Open-Label, Randomized Phase 1b Study of the Safety and Immunogenicity of Investigational Seasonal Influenza DNA Vaccine (HA DNA) followed by Trivalent Inactivated Vaccine (TIV) Administered Intradermally (ID) or Intramuscularly (IM) in Healthy Adults Ages 18-70 Years

Protocol VRC 703

Sponsored by:
National Institute of Allergy and Infectious Diseases (NIAID)
Vaccine Research Center (VRC)
Bethesda, Maryland

BB-IND 15147– held by VRC, NIAID, NIH

NIAID Funding Mechanism:
Contract # HHSN272201000049I

Clinical Trial Management:
Universal Influenza Clinical Program Support Center (UNI-CPSC)
The EMMES Corporation
Rockville, MD 20850

Version 4.0
June 21, 2013

Confidentiality Statement

This document is confidential and is to be distributed for review only to investigators, potential investigators, consultants, study staff, and applicable independent ethics committees or institutional review boards. The contents of this document shall not be disclosed to others without written authorization from NIAID (or others, as applicable), unless it is necessary to obtain informed consent from potential study participants.

Statement of Compliance

The trial will be conducted in compliance with the protocol, the applicable regulatory requirements including but not limited to the U.S. Code of Federal Regulations applicable to clinical studies (45 CFR 46, 21 CFR including parts 50 and 56 concerning informed consent and IRB regulations, and 21 CFR 312 concerning Investigational New Drug (IND) application), International Conference on Harmonization Good Clinical Practice (ICH-GCP) guidance, and the NIAID Clinical Terms of Contract Award. The site will hold a current Federal Wide Assurance (FWA) issued by OHRP for federally funded research. Completion of Protection of Human Subjects Training will be required for all study personnel in accordance with NIH policy.

TABLE OF CONTENTS

INVESTIGATOR OF RECORD PROTOCOL SIGNATURE PAGE 6
ABBREVIATIONS 7
PRÉCIS 9
1 Introduction 11
1.1 Influenza Biology, Natural History and Vaccines 11
1.2 Rationale for Seasonal Influenza DNA Vaccine Prime Followed by TIV Boost 13
1.3 Rationale for Comparison of TIV IM to TIV ID Boost 13
1.4 Previous Human Experience with VRC DNA Vaccines 14
1.4.1 Plasmid DNA Vaccines Developed by VRC, NIAID, NIH 14
1.4.2 Influenza DNA Vaccines Developed by VRC, NIAID, NIH 14
1.5 Assessment of Immunogenicity 17
2 Study Vaccines 18
2.1 Formulation and Manufacturing of VRC-FLUDNA063-00-VP 18
2.2 Fluzone® Intramuscular (TIV IM) Administration 19
2.2.1 2012/13 Fluzone® Influenza Strains 19
2.2.2 2013/14 Fluzone® Influenza Strains 19
2.3 Fluzone® Intradermal (TIV ID) Administration 19
2.4 Pre-Clinical Studies of HA DNA Vaccines 19
3 Study Objectives 20
3.1 Primary objectives: 20
3.2 Secondary objectives: 20
3.3 Exploratory objectives: 20
4 Study Design 21
4.1 Study Population 21
4.1.1 Inclusion Criteria 22
4.1.2 Exclusion Criteria 23
4.2 Study Schedules 24
4.2.1 Pre-enrollment 25
4.2.2 Screening Visit(s) 25
4.2.3 Enrollment Visit 25
4.2.4 Administration of Injections 25
4.2.5 Blood Sample Collection 26
4.2.6 Concomitant Medications and Procedures 27
4.3 Criteria for Beginning Randomization into Groups 3-6 27
4.4 Criteria for Discontinuing Study Injections or Protocol Participation 27
4.4.1 Discontinuation of Study Injections 27
4.4.2 Discontinuation from Protocol Participation 27
4.5 Criteria for Pausing the Study 28
5 Safety and Adverse Events 28
5.1 Adverse Events 28

5.1.1 Adverse Event (AE) Definition 28
5.1.2 Adverse Event Reporting in the Study Database 28
5.2 Serious Adverse Events 29
5.2.1 Serious Adverse Event Definition 29
5.2.2 Reporting Serious Adverse Events to the IND Sponsor 29
5.2.3 IND Sponsor Reporting to the FDA 30
5.3 Reporting to Site IRBs 30
5.4 Safety Monitoring 30
6 Statistical Considerations and Sample Analysis 31
6.1 Overview 31
6.2 Objectives 31
6.3 Endpoints 31
6.3.1 Primary Endpoints: Safety 31
6.3.2 Primary Endpoint: Immune Response 31
6.3.3 Secondary Endpoints: Immune Responses 32
6.3.4 Exploratory Endpoints: Immune Responses 32
6.4 Sample Size and Accrual 32
6.4.1 Power Calculations for Evaluation of Safety 33
6.4.2 Power Calculations for Evaluation of Immune Responses 34
6.4.3 Power Calculations for Primary Immunogenicity Comparisons 34
6.5 Statistical Analysis 36
6.5.1 Analysis Variables 36
6.5.2 Baseline Characteristics 36
6.5.3 Safety Analysis 36
6.5.4 Analysis of Immune Responses 36
6.5.5 Interim Analyses 37
6.5.6 Randomization of Treatment Assignments 37
7 Pharmacy and Vaccine Administration Procedures 38
7.1 Study Agents 38
7.2 Study Agent Presentation and Storage 38
7.2.1 Study Agent Labels 38
7.2.2 Study Agent Storage 38
7.3 Preparation of Study Agent for Injection 39
7.3.1 Preparation of VRC-FLUDNA063-00-VP 39
7.3.2 Preparation of TIV ID and TIV IM 39
7.4 Study Agent Accountability 39
7.4.1 Documentation 39
7.4.2 Disposition 39
8 Human Subjects Protection 40
8.1 Institutional Review Board 40
8.2 Subject Recruitment and Enrollment 40
8.3 Informed Consent 40
8.4 Subject Confidentiality 40
8.5 Risks and Benefits 41
8.5.1 Risks of Blood Collections: 41

8.5.2 Risks of the DNA Vaccine: 41
8.5.3 Risks of the Seasonal TIV: 41
8.5.4 Risks of the Seasonal TIV administered ID: 42
8.5.5 Other Risks: 42
8.5.6 Benefits: 43
8.6 Plan for Use and Storage of Biological Samples 43
8.7 Compensation 43
8.8 Safety Monitoring 43
8.8.1 Protocol Safety Review Team 43
9 Administration and Legal Obligations 44
9.1 Protocol Initiation, Amendments and Termination 44
9.2 Study Documentation and Study Records Retention 44
9.3 Data Collection and Protocol Monitoring 44
9.3.1 Data Capture Methods 44
9.3.2 Source Documents and Access to Source Data/Documents 45
9.3.3 Protocol Monitoring 45
9.4 Language 45
9.5 Policy Regarding Research-Related Injuries 45
10 References 46
Appendix I: Template Informed Consent Form 49
Appendix II: Contact Information 57
Appendix III: Schedule of Evaluations 60
Appendix IV: Assessment of Relationship to Vaccine and Adverse Event Severity Grading 63

INVESTIGATOR OF RECORD PROTOCOL SIGNATURE PAGE
VRC 703

An Open-Label, Randomized Phase 1b Study of the Safety and Immunogenicity of Investigational Seasonal Influenza DNA Vaccine (HA DNA) Followed by Trivalent Inactivated Vaccine (TIV) Administered Intradermally (ID) or Intramuscularly (IM) in Healthy Adults Ages 18-70 Years

Sponsored by:
Vaccine Research Center, National Institute of Allergy and Infectious Diseases
National Institutes of Health

I, the Investigator of Record for the indicated Study Site, agree to conduct this study in full accordance with the provisions of this protocol. I agree to maintain all study documentation pertaining to the conduct of this study, including CRFs, source documents, consent forms, laboratory test results, and medication inventory records, for at least 2 years following approval of a Biologics License Application, unless directed otherwise by the VRC. No study records will be destroyed without prior authorization from NIAID. Publication of the results of this study will be governed by the VRC and NIAID policies. Any presentation, abstract, or manuscript will be made available by the investigators to the VRC Leadership Group and to NIAID for review prior to submission.

I have read and understand the information in this protocol and will ensure that all associates, colleagues, and employees assisting in the conduct of the study are informed about the obligations incurred by their contribution to the study.

______________________________	______________________________
Name /Title of Investigator of Record	Study Site Name/Identifier
______________________________	______________________________
Signature of Investigator of Record	Date

ABBREVIATIONS

Abbreviation	Term
ACIP	Advisory Committee on Immunization Practices
AE	adverse event
ATIV	MF59-adjuvanted trivalent influenza vaccine, FLUAD®
CBC	complete blood count
CC	Clinical Center
CDC	Center for Disease Control
CRO	Contract Research Organization
DNA	deoxyribonucleic acid
EDC	Electronic data capture
ELISA	enzyme-linked immunosorbent assay
ELISpot	enzyme-linked immunospot
EU	European Union
FDA	Food and Drug Administration
GCP	Good Clinical Practices
GMT	Geometric mean titer
HA	Hemagglutinin
HAI	Hemagglutination Inhibition
HIV	human immunodeficiency virus
HLA	human leukocyte antigen
ICS	intracellular cytokine staining
ID	Intradermal
IDES	Internet Data Entry System
IIV	Inactivated Influenza Vaccine
IoR	Investigator of Record
IRB	Institutional Review Board
ITT	Intent-to-treat
LIMS	Laboratory Information Management System
MedDRA®	Medical Dictionary for Regulatory Activities
MF59	proprietary adjuvant (Novartis)
NA	Neuraminidase
NH	Northern Hemisphere
NIAID	National Institute of Allergy and Infectious Diseases
NIH	National Institutes of Health
NVITAL	NIAID Vaccine Immune T-Cell and Antibody Laboratory
PBMC	peripheral blood mononuclear cells
PBS	Phosphate-buffered saline
PSRT	Protocol Safety Review Team
RNA	ribonucleic acid
SAE	serious adverse event
SH	Southern Hemisphere
S-OIV	Swine-Origin Influenza Virus

Abbreviation	Term
TIV	Unadjuvanted trivalent inactivated vaccine (for seasonal influenza)®
UNI-CPSC	Universal Influenza Clinical Program Support Center
VAERS	Vaccine Adverse Events Reporting System
VIS	vaccine information statements
VRC	Vaccine Research Center
WHO	World Health Organization

PRÉCIS

VRC 703: An Open-Label, Randomized Phase 1b Study of the Safety and Immunogenicity of Investigational Seasonal Influenza DNA Vaccine (HA DNA) Followed by Trivalent Inactivated Vaccine (TIV) Administered Intradermally (ID) or Intramuscularly (IM) in Healthy Adults Ages 18-70 Years

Objectives: The primary objectives relate to safety and tolerability of each prime-boost regimen and strain specific HAI titers at 3 weeks after each vaccination. Secondary and exploratory objectives are related to the quality of the immune responses over the period of study follow-up.

Products: HA DNA vaccine (VRC-FLUDNA063-00-VP) was developed by VRC, NIAID and is composed of 3 closed-circular DNA plasmids, each with a CMV/R promoter that encode for the hemagglutinin (HA) from the following 3 influenza strains recommended for 2012/13 influenza vaccines: Influenza A virus (A/California/04/2009) (H1N1), Influenza A virus (A/Victoria/361/2011)-like (H3N2), Influenza B virus (B/Wisconsin/1/2010). HA DNA vaccines have been previously evaluated as safe and able to improve the overall immune response when administered as a prime for inactivated influenza vaccine in humans. The inactivated seasonal influenza vaccines will be the licensed Fluzone®, manufactured by Sanofi Pasteur using the formulations available commercially for intramuscular (IM) or intradermal (ID) administration. The original study design involved evaluation of TIV (AGRIFLU) or adjuvanted TIV (FLUAD) manufactured by Novartis. However, due to uncertainty regarding availability of these products in late Oct 2012, the TIV boost products used in this study were changed by protocol amendment.

Subjects: 330 healthy adults ages 18 to 70 years

Study Plan: This study is evaluating the safety and immunogenicity of same season and sequential season vaccination schedules as shown in the following table.

Group	Subjects	Stratification by age	Prime–Aug/Sep 2012<br>Day 0	Boost-Nov/Dec 2012<br>Week 14	
			2012/13 strains		2013/14 strains
1	55	1a: 18-50 years	HA DNA	TIV ID	-
		1b: 51-64 years			
2	55	2a: 18-50 years	HA DNA	TIV IM	-
		2b: 51-70 years			
Group	Subjects	Stratification by age		Prime-Nov/Dec 2012<br>Day 0	Boost-*Aug-Oct 2013
3	55	3a: 18-50 years		TIV ID	TIV ID
		3b: 51-64 years			
4	55	4a: 18-50 years		TIV IM	TIV IM
		4b: 51-70 years			
5	55	5a: 18-50 years		HA DNA (left arm)<br>TIV ID (right arm)	TIV ID
		5b: 51-64 years			
6	55	6a: 18-50 years		HA DNA (left arm)<br>TIV IM (right arm)	TIV IM
		6b: 51-70 years			
Total	330	* May be delayed pending availability of the licensed TIV products.<br>All vaccinations are open-label and administered in the deltoid.<br>HA DNA injections will be administered via Biojector.<br>TIV injections will be administered using standard needle IM and ID methods.			

The randomization to Groups 1 and 2 occurred first. Randomization to Groups 3-6 occurred later. Randomizations are stratified by age, subgroups "a" include subjects 18-50 years of age and subgroups "b" include subjects 51-70 years of age in the TIV IM group and 51-64 in the TIV ID group (consistent with approvals for these products). Subjects who are 65-70 who have already been enrolled or completed screening at the time of the Version 3.0 amendment will be randomized to the TIV IM groups only. After all the subjects who had screened prior to the Version 3.0 amendment are enrolled, sites will proceed with enrolling only 18-64 year old subjects. This plan is intended to keep similar age distribution to the groups while also being consistent with the age range approvals for the licensed TIV products to be used through next influenza season (2013/2014). Of note, subjects age 64 will only be eligible if they can receive their 2013/14 TIV vaccination prior to their 65th birthday. Study teams will need to consider this in assessing eligibility of subjects who are age 64 at screening. The 2013/14 vaccinations in Groups 3-6 will begin upon availability of the licensed TIV products in August 2013 and are expected to be completed before widespread circulation of influenza in the community begins during the 2013/14 influenza season.

Study Duration: Subjects will be evaluated for safety and immune responses throughout the trial for 24 weeks following the boost. Duration of time on study will be 38 weeks for Groups 1 and 2 and 68 weeks for Groups 3, 4, 5 and 6.

1 INTRODUCTION

The Dale and Betty Bumpers Vaccine Research Center (VRC), NIAID, NIH (Bethesda, MD) is dedicated to translating the latest knowledge of disease pathogenesis and immunology into new vaccine strategies to provide safe and effective means to prevent and control infectious diseases. The 2009-2010 H1N1 pandemic influenza reinforces awareness that rapidly preparing a vaccine for use during an influenza season and consideration of the different levels of immunity and risks present in different age groups in the population is important for public health [1]. The need for influenza vaccines that are both more immunogenic and able to induce universal immune responses effective against a broad spectrum of influenza strains is well recognized. This protocol was originally designed to use DNA vaccine antigen delivery to induce immune responses against native hemagglutinin (HA) structures prior to boosting with licensed subunit inactivated trivalent influenza vaccine (TIV, AGRIFLU®) or with subunit inactivated trivalent influenza vaccine supplemented with MF59 adjuvant (ATIV, FLUAD®). In October 2012, when the FLUAD® and AGRIFLU® 2012/2013 products were suspended for use in other countries and not available to the protocol team in the U.S., the protocol was amended. There is pre-clinical and clinical evidence suggesting that the HA DNA prime-inactivated vaccine boost approach will contribute to development of influenza vaccines that induce cross-reactive immunity against old, new and re-emerging influenza strains, with the potential for improved durability of response [2-4]. The boost comparison in this study was amended to compare intradermal (ID) TIV to Intramuscular (IM) TIV using the licensed ID and IM formulations of Fluzone® (Sanofi Pasteur) for which lots have been released by the U.S. FDA for use in the 2012/2013 season.

In this study, we are evaluating the safety and immunogenicity of same season and sequential season vaccination schedules. The same season regimens (2012/13 prime and boost with a 14 week interval) consist of HA DNA prime with TIV ID boost -- or -- HA DNA prime with TIV IM boost; the active comparator for these regimens will be TIV ID or TIV IM alone because this is the standard for adult influenza vaccination within a single season. The sequential season regimens (2012/13 prime and 2013/14 boost) consist of concurrent administration (in different arms) of HA DNA and TIV ID prime with TIV ID boost -- or -- HA DNA and TIV IM prime with TIV IM boost; the active comparator for these regimens will be TIV ID followed by TIV ID boost or TIV IM followed by TIV IM boost. For the sequential season regimens, the interval will be about 7 months or longer, consistent with the use of these licensed products for sequential season vaccinations. Evaluation of these investigational schedules and active comparator schedules will inform development of novel influenza vaccine strategies that may offer improved and cross-protective immunity against antigenically diverse influenza strains.

1.1 INFLUENZA BIOLOGY, NATURAL HISTORY AND VACCINES

Influenza is a negative-strand ribonucleic acid (RNA) virus with a segmented genome that belongs to the family *Orthomyxoviridae*. Of the three genera of influenza circulating in nature (influenza A, B, and C), only the first two are known to cause epidemics [5]. Influenza A viruses have 8 open reading frames that encode 10 viral proteins. They are classified on the basis of the antigenicity of their surface glycoproteins: hemagglutinin (HA) and neuraminidase (NA). Sixteen HA subtypes and 9 NA subtypes are known to exist, but only three HA subtypes (H1, H2, and H3) and two NA subtypes (N1 and N2) have caused significant human epidemics [6].

The public health burden of influenza in the world is enormous. Annual influenza epidemics cause about 250,000 to 500,000 deaths worldwide [7]. Circulating viruses change quickly and

re-assort with each other creating new viruses. These present an immediate threat to public health and currently require the preparation of new vaccines directed at the changing viral strains that are prevalent annually. Emerging virus strains present the potential of a pandemic when there is little or no pre-existing immunity in the population as observed in worldwide outbreaks of influenza in the last century [8]. A global pandemic caused by spread of a novel swine-origin influenza virus (S-OIV) in the 2009-2010 influenza season is a recent example of this challenge to public health.

Vaccines are an effective way of preventing influenza infection and transmission in humans. Annually, the World Health Organization (WHO) and the U.S. FDA make recommendations on the composition of the seasonal influenza vaccine, with recommendations for the Northern Hemisphere (NH) and for the Southern Hemisphere (SH) considered at different times based on epidemiology data. The annually licensed influenza vaccines consist of 3 components: influenza A (H1N1), influenza A (H3N2), and an influenza B virus strain. These vaccines depend upon labor-intensive methods that limit manufacturing capacity and have low immunogenicity to induce type-specific responses in some vulnerable populations. Currently, the vaccine composition requires an adjustment for emerging antigenically-modified influenza strains and efficacy is limited in vulnerable populations.

In February 2013, the Advisory Committee on Immunization Practices (ACIP) recommended changing the influenza vaccine nomenclature such that the abbreviation “TIV” would be replaced with “IIV3” to indicate a trivalent inactivated influenza vaccine [9]. We acknowledge this recommendation; however, the change has not been implemented in the VRC 703 protocol to maintain consistency with all previously issued study documentation.

For seasonal influenza infection, the rates of serious illness and death are the highest among persons older than 65 years, children of less than 2 years old, and persons of any age who have medical conditions that place them at increased risk for complications from influenza [10]. Seasonal influenza causes significantly higher morbidity and mortality in older adults than in younger adults and these adverse outcomes are attributed to an age-related decline in immune function [11].

Seasonal TIVs have demonstrated a clinical efficacy of 70% to 90% in younger adults, ~ 58% in those aged 60 to 69 years, and 30% to 40% in those aged 70 or more years [12]. When the seasonal vaccine and circulating viruses are antigenically similar, seasonal TIV prevents laboratory-confirmed influenza illness among approximately 70%-90% of healthy adults less than 65 years old in randomized controlled trials. Immunization efficacy was 47%-77% in studies conducted during influenza seasons when the vaccine strains were not an exact antigenic match to the majority of circulating influenza strains [10].

In the 2009-2010 H1N1 influenza pandemic, the CDC determined that the seasonal TIV was unlikely to provide protection against a novel pandemic influenza A (H1N1) that was an antigenically distinct influenza virus to which little or no pre-existing immunity was detected in the population [1]. The epidemiologic pattern of this novel swine-origin influenza virus (S-OIV) strain included a high prevalence (60% of infected patients) in the population ≤ 18 years old, who had not been exposed to the influenza strains that circulated in 1950-1970s and had partial antigenic similarity with the novel H1N1 strain [1, 13]. Using stored serum specimens collected in previous vaccine studies, CDC assessed the level of cross-reactive antibodies to the novel influenza A (H1N1) virus in cohorts of children and adults before and after they had been

vaccinated with the 2005-2009 influenza seasonal vaccines [1]. The results indicated that 33% of adults older than 60 years and 6-9% of adults aged 18-64 years had some cross-reactive antibody to the novel influenza, and no cross-reactive antibody was found in children. The results also suggested that seasonal influenza vaccines are unlikely to provide protection against novel emerging influenza virus strains.

In the pursuit of more universal influenza vaccine, several conserved regions have been identified in influenza viral protein structures that may serve as broad neutralizing epitopes to afford cross-protection against a variety of influenza strains [2, 14, 15]. NIAID scientists have been engaged in investigation of immune responses to influenza and identification of the broadly-neutralizing antibodies that may lead to a development of a more universal influenza vaccine [2, 3]. The HA DNA vaccine prime-inactivated vaccine boost strategy that will be evaluated in this protocol has shown evidence of improved immune response, including to epitopes conserved between influenza subtypes [4]. Using the ID formulation of seasonal TIV in the HA DNA prime-TIV boost regimen will contribute additional information to a series of clinical trials being conducted with same season and sequential season HA DNA prime and inactivated vaccine boost.

1.2 Rationale for Seasonal Influenza DNA Vaccine Prime Followed by TIV Boost

DNA vaccines have the potential to be manufactured rapidly. They are known to induce balanced immune responses that induce both humoral and cellular immunity. The experience to date with HA DNA vaccines warrants continued investigation. One approach to improving immunity to influenza would be to prime the population with an HA DNA vaccine early in the year and to boost with TIV when the influenza season begins in order to provide better immunogenicity, especially to novel influenza strains. This may be a useful strategy for the older adult and pediatric populations for which the TIV vaccine alone has a lower efficacy than in young adults. In previous studies, the VRC has found that antibody responses are higher after the boost when there is a long prime-boost interval, such as 6 months compared to a short interval, such as 1 month [4]. For this reason, single dose DNA priming schedules with a long interval between prime and boost are being evaluated towards the goal of an influenza vaccination strategy with improved immunogenicity.

A DNA vaccine may also elicit CD8 T cell responses to conserved HA epitopes that may afford some cross-protection [16-18]. It has been also suggested that T cell responses are the important correlates of influenza vaccine protection, especially in the elderly populations immunized with the seasonal flu vaccines [19, 20].

1.3 Rationale for Comparison of TIV IM to TIV ID Boost

Intradermal administration of TIV has been widely studied and shown to be safe and immunogenic [21-23]. The intradermal product to be used in this study was approved by the U.S. FDA in May 2011 for use in adults 18-64 years old. The ID method of administration is dose-sparing and has the potential to make more doses of influenza vaccine available in a given year from the same amount of bulk supply. Route of administration is also known to affect the type and magnitude of immune responses induced. A comparison of IM to ID boost of HA DNA primed responses will contribute to a better understanding of how to improve immune responses to influenza antigens.

1.4 PREVIOUS HUMAN EXPERIENCE WITH VRC DNA VACCINES

1.4.1 Plasmid DNA Vaccines Developed by VRC, NIAID, NIH

Investigators at the VRC/NIAID/NIH in Bethesda, MD have evaluated plasmid DNA vaccine strategies targeted to several different infectious diseases since 2001 in preclinical and clinical studies. Cumulatively through March 2012, more than 2300 study subjects have been vaccinated with VRC DNA vaccines. Dosages up to 8 mg have been administered, with the majority of injections being at a 4 mg dosage. Data from dose-escalation studies indicate that a 4 mg dosage offers the combination of a good safety profile and greater ease of administration than an 8 mg dosage, as well as reliable immunogenicity as indicated by laboratory measures of immune response. Preclinical and clinical evaluations to date of several plasmid DNA vaccines support the safety and immunogenicity of VRC DNA vaccines at the 4 mg dosage. The results of human clinical trials with VRC DNA vaccines have been published for HIV DNA vaccines [24-31], an Ebola virus DNA vaccine [32], a SARS DNA vaccine [33], West Nile virus DNA vaccines [34, 35] and H5 DNA vaccine [4]. VRC clinical trials of WNV and SARS vaccines have provided evidence that a DNA vaccine can induce neutralizing antibody as assessed by a reporter virus particle (RVP) neutralizing antibody assay [33-35]. Experience to date indicates that there may be advantages to using a Biojector for DNA vaccine delivery as compared to using a standard needle and syringe [36].

1.4.2 Influenza DNA Vaccines Developed by VRC, NIAID, NIH

The VRC/NIAID/NIH has developed several investigational influenza DNA vaccine products. Clinical evaluation of the first influenza DNA product was initiated in 2006 with an avian influenza H5 DNA vaccine. Through a series of nine clinical trials, influenza DNA vaccines have been tested alone or in prime-boost regimens with the respective inactivated vaccines in the clinical trials as shown in **Table 1-1**. Seven of these studies are complete, while VRC 701 and VRC 702 were ongoing when VRC 703 began.

Table 1-1: Experience with VRC Influenza DNA Vaccines in Adults and Children

IND Number	Vaccine(s)	Antigen(s)	Protocols
BB-IND 13197	VRC-AVIDNA036-00-VP	HA of A/Indonesia/05/2005 (H5N1)	VRC 304<br>VRC 305
BB-IND 13836	VRC-AVIDNA036-00-VP (with inactivated vaccine boost)	HA of A/Indonesia/05/2005 (H5N1)	VRC 306<br>VRC 310
BB-IND 13939	VRC-FLUDNA047-00-VP (with inactivated vaccine boost)	Trivalent HA matching the 2008-2009 seasonal strains	VRC 307
	VRC-FLUDNA056-00-VP (with inactivated vaccine boost)	Trivalent HA matching the 2009-2010 seasonal strains	VRC 309
	VRC-FLUDNA061-00-VP (with inactivated vaccine boost)	Trivalent HA matching the 2011-2012 seasonal strains	VRC 701
BB-IND 14093	VRC-FLUDNA057-00-VP (with inactivated vaccine boost)	HA of pandemic A/California/04/2009 (H1N1)	VRC 308
BB-IND 15062	VRC-FLUDNA063-00-VP (with inactivated vaccine boost)	Trivalent HA matching the 2012-2013 seasonal strains	VRC 702

H5 DNA vaccine alone (BB-IND 13197) was evaluated in two Phase 1 studies. One study (VRC 304) investigated intramuscular (IM) administration of the vaccine or placebo in three groups of healthy adults 18-60 years of age who received 3 IM injections of 1 mg vaccine, 4 mg vaccine, or placebo, respectively. All injections in VRC 304 were via Biojector. The second study, VRC 305, investigated intradermal (ID) administration of the vaccine by 4 schedules: 0.5 mg ID by needle, 0.5 mg ID by Biojector, 0.5 mg ID x2 in the same arm, and 0.5 mg ID in each arm. Cumulatively, 89 subjects were enrolled. Between the two studies, 204/222 (92%) of the expected DNA vaccinations were administered to 74 vaccine recipients and 42/45 (93%) of expected phosphate buffered saline (PBS) injections were given to 15 placebo recipients.

The H5 DNA vaccine was well tolerated by both the IM and ID routes. Detailed final study reports are available in the BB-IND 13197. In VRC 304, no apparent dose effect on frequency, duration, or severity of reactogenicity was noted. There were two serious adverse events (SAE) in VRC 304: a Grade 3 leukocytosis, attributed to concomitant medications taken for a musculoskeletal injury and a Grade 3 paroxysmal hemicrania in a subject with a history of severe headaches. A neurologist assessed this as unlikely to be related to vaccine. In VRC 305, there were no SAEs. The H5 DNA vaccine alone was not strongly immunogenic by either the IM or ID routes.

H5 DNA Vaccine in prime-boost regimens (BB-IND 13836) was evaluated in two Phase 1 studies (VRC 306 and VRC 310) in healthy adults ages 18-60 years old. Between these two studies the prime-boost regimens evaluated included two doses of monovalent inactivated vaccine (MIV, Sanofi Pasteur H5N1 A/Indonesia 05/05 avian influenza vaccine) at 4 or 24week intervals or H5 DNA vaccine followed by MIV at varying intervals from 4 to 24 weeks. Cumulatively, 124 subjects were enrolled. Between the two studies, 114/114 (100%) of the expected H5 DNA injections were administered to 99 subjects randomized to schedules with H5 DNA primes and 147/149 (99%) of the expected H5N1 MIV injections were administered to 122 subjects.

There was no severe local or systemic reactogenicity. Mild local reactogenicity was reported in a majority of the H5 DNA vaccine recipients (83%) while 2% reported moderate local reactogenicity. The majority of the H5 DNA vaccine recipients reported no systemic reactogenicity; 23% reported mild and 5% reported moderate systemic reactogenicity. No SAE were reported following the H5 DNA vaccinations; there was one SAE reported in the follow-up period through 48 weeks: multiple injuries unrelated to study participation in the period after the H5N1 booster. All adverse events following H5 DNA injection were mild (Grade 1) or moderate (Grade 2) except for one severe (Grade 3) case of gastroenteritis with onset 15 days after receiving H5 DNA vaccination, which was assessed as unrelated to vaccine.

In assessing immunogenicity of H5 DNA vaccine followed by MIV, a single H5 DNA vaccine prime followed by a single MIV boost at short intervals (4-8 weeks) did not significantly improve HAI titers over levels achieved with MIV alone. However, a single H5 DNA 4 mg vaccination prime significantly improved HAI responses when the MIV boost interval was 24 weeks, as compared with two doses of MIV at the same interval [4]. In VRC 310, an effect was observed with 12-16 week DNA-MIV boost intervals. Therefore, a single dose of DNA priming may be sufficient to increase the magnitude and breadth of HA-specific antibody responses.

Seasonal HA DNA vaccine studies: Specifically, with regard to seasonal influenza vaccines developed by the VRC/NIAID/NIH, VRC-FLUDNA047-00-VP, VRC-FLUDNA056-00-VP,

VRC-FLUDNA061-00-VP (BB-IND 13939 and BB-IND 14093) and VRC-FLUDNA063-00-VP (BB-IND 15062) are season-specific 3-plasmid DNA vaccines; while VRC-FLUDNA057-00-VP (BB-IND 14093) is a single plasmid H1 DNA based on the 2009 pandemic H1N1 influenza virus. **Table 1-2** shows the composition of these investigational influenza DNA vaccines.

Table 1-2: Composition of VRC Seasonal Influenza DNA Vaccine Products

Product/IND reference	**Plasmids and Expressed Antigens**							
	**H1**		**H3**			**B**		
	VRC-9269	VRC-9328	VRC-9270	VRC-2439	VRC-3027	VRC-9271	VRC-9323	VRC-2722
	A/Brisbane 59/07	A/California 04/09	A/Brisbane 10/07	A/Perth 16/09	A/Victoria 361/11	B Florida 4/06	B Brisbane 60/08	B Wisconsin 1/10
VRC-FLUDNA047-00-VP Seasonal 2008-09/IND 13939	X		X			X		
VRC-FLUDNA056-00-VP Seasonal 2009-10/IND 13939	X		X				X	
VRC-FLUDNA057-00-VP Pandemic H1 2009/IND 14093		X						
VRC-FLUDNA061-00-VP Seasonal 2011-12/IND 13939		X		X			X	
VRC-FLUDNA063-00-VP Seasonal 2012-13/IND 15062		X			X			X

Three clinical trials, VRC 307 (2008/09 season), VRC 308 (2009 H1 pandemic influenza) and VRC 309 (2009/10 season) have been completed in adults with seasonal HA DNA vaccines. There is one ongoing clinical trial (VRC 701) for which the administration of the blinded HA DNA (or placebo) prime injections (based on 2011/2012 vaccine strains) was completed in February 2012 and the sequential season TIV boost with Fluzone® IM is ongoing in the 2012/2013 influenza season with a 36 week boost interval. The VRC 702 trial in adolescents and children started in June 2012 with the administration of 2012/2013 HA DNA vaccine primes. VRC 702 includes age-matched groups that receive 2012/2013 TIV primes (Fluzone® IM) and all participants will receive TIV IM boosts with an 18 week boost interval. The amendment of VRC 703 to use Fluzone® by IM or ID route at a 14 week same season interval or on a sequential schedule will contribute to fully evaluating boost intervals, route of administration and homologous vs. heterologous strains in influenza HA DNA prime-inactivated boost regimens.

In VRC 307 and VRC 309, the DNA constructs were the same except for the encoded influenza B antigen. Cumulatively, a total of 111 subjects were enrolled; 66 were randomized to a single HA DNA prime-TIV boost, 25 to a single PBS prime-TIV boost, and 20 to TIV prime-TIV boost schedules. Between the two studies 65/66 (99%) of the expected HA DNA vaccinations, 25/25 (100%) of the expected PBS injections and 126/131 (96%) of the expected TIV vaccinations were administered. The seasonal HA DNA vaccines were well tolerated. There was no severe local or systemic reactogenicity. Similar to the H5 DNA vaccine, a majority of seasonal HA DNA vaccine recipients (78%) experienced mild local reactogenicity and 2% experienced moderate local reactogenicity. The majority (66%) of HA DNA vaccine recipients reported no

systemic reactogenicity, while 31% reported, mild and 3% reported moderate systemic reactogenicity. No SAE were reported in either VRC 307 or VRC 309. All adverse events were mild or moderate except for three Grade 3 events following HA DNA prime injections (urticaria at one day post vaccination, influenza at 11 days post vaccination, and increased alanine aminotransferase [ALT] at 33 days post vaccination); one Grade 3 event following PBS prime injection (neutropenia at 14 days post injection); and one Grade 3 event (gastroenteritis at 24 days post vaccination) following TIV. Based on temporal relationship, urticaria was the only Grade 3 event attributed as possibly related to DNA vaccine.

VRC 308 included 20 subjects on a schedule of three H1 DNA vaccinations at 4 week intervals; 60/60 (100%) of expected H1 DNA vaccines were administered and the vaccine was well tolerated. When the licensed H1N1 inactivated vaccine became available, 18 subjects opted to receive the boost as well. There was no severe local or systemic reactogenicity. The majority (90%) of H1 DNA vaccine recipients reported mild local reactogenicity; one subject (5%) reported moderate local reactogenicity. Over 3 injections with H1 DNA vaccine, 60% reported mild and 15% reported moderate systemic reactogenicity. There were no SAEs and all adverse events were mild or moderate in severity.

VRC 701 included 131 subjects (18-70 years) enrolled in Jan-Feb 2012 who were randomized to receive a blinded injection of 2011/12 HA DNA vaccine or placebo, which was followed by a boost with the 2012/13 TIV in Fall 2012. All study visits have been completed and data analysis is in progress. VRC 702 includes 75 subjects (6-17 years) enrolled in June-August 2012 who were randomized to receive 2012/2013 HA DNA vaccine or TIV prime, which was followed by a boost with the 2012/13 TIV. The HA DNA prime injections were well tolerated.

<u>Summary of HA DNA safety and immunogenicity</u>: The safety data for the VRC HA DNA vaccine trials to date shows that these vaccines were well tolerated. No severe local or systemic reactogenicity occurred nor were there any serious adverse events related to vaccine. The pattern of local and systemic reactogenicity appears to be generally mild and similar across the studies, independent of the antigens encoded by the HA DNA constructs included in any one vaccine.

Regarding immunogenicity, seasonal HA DNA priming did not significantly improve HAI response compared to TIV alone with the boost administered at a 3-4 week interval. Data from studies of the H5 DNA vaccine using various H5N1 inactivated vaccine (MIV) boost intervals show that a longer interval is associated with a higher magnitude of immune response [4].

Based on experience with the H5 DNA vaccine, future development of the trivalent seasonal HA DNA is directed towards evaluation of an inactivated boost at intervals 12 weeks or longer post-prime as a potential method of reliably inducing a strong immune response to seasonal influenza vaccine. The immunogenicity data from the 36 week boost interval in VRC 701 study in adults and 18 week boost in VRC 702 study in children was not available before VRC 703 initiation.

1.5 Assessment of Immunogenicity

In protocol VRC 703, specimens to evaluate immunogenicity will be taken at baseline and at specified time points. The primary immunogenicity timepoint is 3 weeks after the boost. Measurements of antibody, B cell and T cell responses will be assessed. Hemagglutinin (HA)-specific antibody as measured by HAI assay is the traditional benchmark measure of immune response to influenza vaccines and will be conducted on stored samples obtained throughout the study. The HA-specific immune responses will be assessed through 24 weeks after the boost.

Because these adult study subjects are likely to have pre-existing immune responses to many influenza hemagglutinin antigens and the effect of vaccination on other humoral and T cell responses is of interest, a variety of exploratory evaluations of immunogenicity may also be performed. HA-specific T cell responses measured by intracellular cytokine staining (ICS) assay or ELISpot, other HA-antibody assays, and assays to evaluate cross-reactivity will be performed at timepoints throughout the study as exploratory evaluations.

The detection of antibody by HAI assay is based on a validated laboratory method. The ICS assay is based upon previously published methods [37] and quantitates the frequency of $CD4^+$ and $CD8^+$ cells that produce interleukin-2, interferon-gamma, or TNF-alpha in response to pools of overlapping peptides representing HA antigens. Specific peptides will also be used to detect T cell responsiveness by an ELISpot assay, modified from a previously published method [38]. Research samples for immunogenicity assays will be processed by the NIAID Vaccine Immune T Cell and Antibody Laboratory (NVITAL) in Gaithersburg, MD, where many of the immunogenicity assays will also be performed. Some immunogenicity assays may be performed by VRC laboratories in Bethesda, MD, approved contract laboratories, or approved research collaborators.

2 STUDY VACCINES

2.1 FORMULATION AND MANUFACTURING OF VRC-FLUDNA063-00-VP

The VRC-FLUDNA063-00-VP Investigational Drug Substance consists of three closed-circular plasmid DNA macromolecules (VRC-9328, VRC-3027 and VRC-2722), in equal amounts by weight; each 1 mL single dose contains 4 mg total. The plasmid DNA expresses influenza HA sequences for strains that meet the criteria for production of the 2012/13 licensed seasonal influenza vaccine, as follows:

- VRC-9328: HA (Gene Bank Protein Accession #ACQ76318)-Influenza A virus (A/California/04/2009) (H1N1),
- VRC-3027: HA (EpiFlu Accession #EPI353906)-Influenza A virus (A/Victoria/361/2011) (H3N2),
- VRC-2722: HA (NCBI Accession # AET22022)-Influenza B virus (B/Wisconsin/1/2010)

The plasmid CMV/R promoter consists of translational enhancer region of the CMV immediate early region 1 enhancer (CMV-IE) substituted with the 5′-untranslated human T-cell leukemia virus type 1 (HTLV-1) R-U5 region of the human T-cell leukemia virus type 1 HTLV-1 long terminal repeat (LTR), and has been shown to increase expression of the encoded gene in comparison to the CMV promoter [39]. This promoter has been evaluated in preclinical safety studies as well as in many clinical trials.

VRC-FLUDNA063-00-VP is manufactured at the VRC/NIAID/Vaccine Pilot Plant (VPP) using plasmid DNA received from the VRC to produce clinical trial material under current Good Manufacturing Practices (cGMP). The process for manufacturing, filling, and packaging the vaccine is summarized in the Investigator’s Brochure (IB). Briefly, the plasmids used in the Master Cell Banks (MCB) were synthesized using human preferred codons as previously described [40]. The plasmids were then transferred to the VPP and their sequences confirmed before use. Each plasmid was used to transform the *Escherichia coli* bacterial host strain, DH5α, in order to produce individual MCB. Each MCB was expanded in culture and inoculated into a

fermentor for production. Bacterial cell growth was dependent upon the cellular expression of the kanamycin resistance protein encoded by a portion of the plasmid DNA. Following growth of bacterial cells harboring the plasmid, the plasmid DNA was purified from cellular components, concentrated, filtered, and stored until formulation of the drug product. The final vaccine product will meet lot release specifications prior to administration.

The Drug Product is manufactured in phosphate buffered saline (PBS). Vials are aseptically filled to a volume of 1.2 mL with 4 mg/mL of plasmids.

2.2 Fluzone® Intramuscular (TIV IM) Administration

Study sites will refer to the package insert for complete product information regarding the TIV products used in this study. Fluzone® for IM administration is manufactured by Sanofi Pasteur, and each 0.5 mL single dose contains 45 mcg total comprised of 15 mcg of influenza virus hemagglutinin of 3 different influenza strains used for the current influenza season.

2.2.1 2012/13 Fluzone® Influenza Strains

The TIV used for the booster injection at Week 14 in Group 2 and for the prime in Group 4 and Group 6 is Fluzone®, a licensed Northern Hemisphere (NH) product approved by the U.S. FDA for administration in the 2012/13 influenza season and composed of the following three influenza strains:

- A/California/07/2009 NYMC X-179A (H1N1)
- A/Victoria/361/2011 IVR-165 (H3N2)
- B/Texas/6/2011 (a B/Wisconsin/1/2010-like virus)

2.2.2 2013/14 Fluzone® Influenza Strains

The TIV used for the last study injection in Group 4 and Group 6 will be Fluzone®, a licensed NH product approved by the U.S. FDA for administration during the 2013-14 influenza season. The composition of 2013/14 TIV will be as follows:

- A/California/7/2009 (H1N1)-like virus,
- A(H3N2) virus antigenically like the cell-propagated prototype virus A/Victoria/361/2011
- B/Massachusetts/2/2012-like virus

2.3 Fluzone® Intradermal (TIV ID) Administration

Study sites will refer to the package insert for complete product information regarding the TIV products used in this study. Fluzone® for ID administration is manufactured by Sanofi Pasteur, and each 0.1 mL single dose contains 27 mcg total comprised of 9 mcg of influenza virus hemagglutinin of 3 different influenza strains used for the current influenza season. Fluzone® ID is supplied as a single-dose prefilled microinjection system. The TIV strains used for ID administration will be the same as used for IM injections as described in Section 2.2.

2.4 Pre-Clinical Studies of HA DNA Vaccines

No preclinical pharmacology, toxicology, pharmacokinetic, or metabolism studies were conducted for the seasonal influenza DNA vaccine VRC-FLUDNA063-00-VP. There is extensive clinical experience with licensed seasonal influenza products, including the antigens present in the 2011/12 seasonal influenza vaccine, as well as human clinical trial experience with

DNA vaccines constructed using the same plasmid backbone and CMV/R promoter, including other seasonal influenza DNA vaccines.

FDA recommendations permit manufacturers with established seasonal flu vaccines to file supplemental applications each year after changing one or more of the three influenza strains in the vaccine. This reflects the large safety experience with similar influenza vaccine products as well as the severe time constraints on design and manufacture of both investigational and licensed vaccines due to the seasonality of influenza infections. Although VRC is evaluating investigational vaccines based on the DNA platform, the influenza gene inserts mimic the antigens found in the yearly seasonal TIV and our approach has been similar in this regard.

Refer to the Investigator's Brochure for more information about preclinical studies.

3 STUDY OBJECTIVES

For the study objectives, an HAI response (seroconversion) is defined as $\geq$1**:**40 for baseline-negative subjects or 4-fold greater than baseline for subjects with pre-existing immunity.

3.1 PRIMARY OBJECTIVES:

- To evaluate the safety and tolerability of each vaccination schedule.
- To evaluate the safety and tolerability of concurrent administration of the HA DNA vaccine and either TIV ID or TIV IM administered in different arms on the same day.
- To compare the frequency and magnitude of the 2012/13 influenza vaccine strain-specific HAI response at 3 weeks after the TIV ID boost in Group 1 to 3 weeks after the TIV ID prime in Group 3, as well as to compare the same responses at 3 weeks after the TIV IM boost in Group 2 to 3 weeks after the TIV IM prime in Group 4.

3.2 SECONDARY OBJECTIVES:

- To evaluate the frequency and magnitude of strain-specific HAI responses at 3 weeks after each study injection for all Groups for the respective 2012/13 and 2013/14 influenza vaccine strains.
- To evaluate the frequency and magnitude of 2012/13 and 2013/14 influenza vaccine strain-specific H1, H3 and B neutralizing antibodies that are 4-fold greater than baseline at 3 weeks after each study injection for all Groups.
- To compare the frequency and magnitude of the 2012/13 influenza vaccine strain-specific HAI responses at 3 weeks after each TIV ID injection in Groups 1, 3, and 5 to the HAI responses after the respective TIV IM injections in Groups 2, 4, and 6.

3.3 EXPLORATORY OBJECTIVES:

- To evaluate the presence of anti-stem antibodies pre- and post-vaccination for all Groups.
- To evaluate the magnitude, frequency, breadth and quality of antibody or T cell responses for all Groups over the time course of the study.

4 STUDY DESIGN

This is a Phase 1b, randomized study in healthy adults to assess the safety, tolerability, and immunogenicity of same season and sequential season vaccination schedules as shown in **Table 4-1** below. The hypotheses are: the HA DNA vaccine will be safe for human administration; the schedules that include an HA DNA vaccination will elicit a higher titer antibody response than the schedules with TIV ID or TIV IM only; and the response will have broader neutralizing activity against antigenically diverse influenza strains.

The schema shows the starting month for Groups 1 and 2 as August 2012, and the boost month for Groups 3, 4, 5 and 6 as starting in August 2013. This timing is premised on the experience in recent years as to when supplies of inactivated vaccine become available for shipping to sites. The actual interval for each subject will be calculated. A notice will be distributed to sites to address any issues related to scheduling visits for administration of vaccinations and to keep the intervals comparable between the groups to the degree possible.

Table 4-1. Study schema

<table>
<tr><th rowspan="2">Group</th><th rowspan="2">Subjects</th><th rowspan="2">Stratification by age</th><th>Prime–Aug/Sep 2012
Day 0</th><th>Boost-Nov/Dec 2012
Week 14</th><th></th></tr>
<tr><th colspan="2">2012/13 strains</th><th>2013/14 strains</th></tr>
<tr><td rowspan="2">1</td><td rowspan="2">55</td><td>1a: 18-50 years</td><td rowspan="2">HA DNA</td><td rowspan="2">TIV ID</td><td rowspan="2">-</td></tr>
<tr><td>1b: 51-64 years</td></tr>
<tr><td rowspan="2">2</td><td rowspan="2">55</td><td>2a: 18-50 years</td><td rowspan="2">HA DNA</td><td rowspan="2">TIV IM</td><td rowspan="2">-</td></tr>
<tr><td>2b: 51-70 years</td></tr>
<tr><th>Group</th><th>Subjects</th><th>Stratification by age</th><th></th><th>Prime-Nov/Dec 2012
Day 0</th><th>Boost-*Aug-Oct 2013</th></tr>
<tr><td rowspan="2">3</td><td rowspan="2">55</td><td>3a: 18-50 years</td><td rowspan="2"></td><td rowspan="2">TIV ID</td><td rowspan="2">TIV ID</td></tr>
<tr><td>3b: 51-64 years</td></tr>
<tr><td rowspan="2">4</td><td rowspan="2">55</td><td>4a: 18-50 years</td><td rowspan="2"></td><td rowspan="2">TIV IM</td><td rowspan="2">TIV IM</td></tr>
<tr><td>4b: 51-70 years</td></tr>
<tr><td rowspan="2">5</td><td rowspan="2">55</td><td>5a: 18-50 years</td><td rowspan="2"></td><td rowspan="2">HA DNA (left arm)
TIV ID (right arm)</td><td rowspan="2">TIV ID</td></tr>
<tr><td>5b: 51-64 years</td></tr>
<tr><td rowspan="2">6</td><td rowspan="2">55</td><td>6a: 18-50 years</td><td rowspan="2"></td><td rowspan="2">HA DNA (left arm)
TIV IM (right arm)</td><td rowspan="2">TIV IM</td></tr>
<tr><td>6b: 51-70 years</td></tr>
<tr><td>Total</td><td>330</td><td colspan="4">* May be delayed pending availability of the licensed TIV products.
All vaccinations are open-label and administered in the deltoid.
HA DNA injections will be administered via Biojector.
TIV injections will be administered using standard needle IM and ID methods.</td></tr>
</table>

The expected duration of time on study per subject is approximately 38 weeks for Groups 1-2 and 68 weeks for Groups 3-6.

4.1 STUDY POPULATION

The study was designed for healthy adults ages 18-70 years, with enrollment stratified by age groups to have an approximately equal representation of healthy younger (18-50 years) and older (51-70 years) adults in the comparison groups. Volunteers ages 65 and older will not enter the

screening process after notice of the amendment plan is distributed to sites given that the protocol is amended to change booster products and the ID formulation is approved for use only through age 64. Volunteers age 64 will only be screened if the study team determines that their 65th birthday will not occur prior to their sequential season booster injection. This will allow for any subject randomized to ID injection next season to still remain within the recommended age range for this licensed product. Volunteers who were already screened at the time of the amendment who are too old to be in a TIV ID group will be randomized only to Groups 3 and 5 and randomization of all such subjects will be permitted only through November 16, 2012.

The following eligibility criteria will be used:

4.1.1 Inclusion Criteria

A subject must meet all of the following criteria:

1. 18 to 70 years old; volunteers who will be older than 64 during the 2013/2014 influenza season will not be enrolled after November 16, 2012.

2. Available for clinical follow-up through Study Week 38 for Groups 1-2 and through Study Week 68 for Groups 3-6

3. Able and willing to complete the informed consent process

4. Willing to donate blood for sample storage to be used for future research

5. Physical examination and laboratory results without clinically significant findings and a Body Mass Index (BMI) ≤40 within the 70 days prior to enrollment

6. Has not yet received the current year (2012/13) influenza vaccine prior to enrollment and agrees to receive seasonal influenza vaccines during study participation only from the study site

Laboratory Criteria within 70 days prior to enrollment:

7. Hemoglobin within institutional normal limits

8. White blood cells either within institutional normal range or accompanied by site physician approval as consistent with healthy adult status

9. Platelets = 125,000 – 500,000/mm^3

10. Alanine aminotransferase (ALT) ≤ 2.5 x upper limit of normal (ULN)

11. Serum creatinine ≤ 1 x ULN based on site institutional normal range

Criteria applicable to women of childbearing potential:

12. Negative human chorionic gonadotropin (β-HCG) pregnancy test (urine or serum) on day of enrollment

13. Agree to use an effective means of birth control from 21 days prior to enrollment through 3 weeks after the second study vaccination

4.1.2 Exclusion Criteria

A subject will be excluded if one or more of the following conditions apply:

Women Specific:

1. Breast-feeding or planning to become pregnant while participating in the study

Subject has received any of the following substances:

2. More than 10 days of systemic immunosuppressive medications or cytotoxic medications within the 12 weeks prior to enrollment or any within the 14 days prior to enrollment

3. Blood products within 16 weeks prior to enrollment

4. Immunoglobulin within 8 weeks prior to enrollment

5. Investigational research agents within 28 days (4 weeks) prior to enrollment or planning to receive investigational products while on the study.

6. Allergy treatment with antigen injections, unless on maintenance schedule and allergy shots could be staggered with the study vaccinations, within 14 days (2 weeks) prior to enrollment

7. Current anti-TB prophylaxis or therapy

Subject has a history of any of the following clinically significant conditions:

8. Contraindication to receiving an FDA-approved seasonal influenza vaccination

9. Serious reactions to vaccines that preclude receipt of study vaccinations, as determined by the site investigator

10. Hereditary angioedema (HAE), acquired angioedema (AAE), or idiopathic forms of angioedema

11. Asthma that is severe, unstable or required emergent care, urgent care, hospitalization or intubation during the previous two years or that is expected to require the use of oral, intravenous or high dose inhaled corticosteroids

12. Diabetes mellitus type I

13. Thyroid disease that is not well-controlled

14. Generalized idiopathic urticaria within the 1 year prior to enrollment

15. Hypertension that is not well controlled

16. Bleeding disorder diagnosed by a doctor (e.g. factor deficiency, coagulopathy, or platelet disorder requiring special precautions), or significant bruising or bleeding difficulties with IM injections or blood draws, or use of blood thinners such as Coumadin or Plavix®

17. Malignancy that is active or treated malignancy for which there is not *reasonable* assurance of sustained cure or malignancy that is likely to recur during the period of the study

18. Seizure disorder other than: 1) febrile seizures, 2) seizures secondary to alcohol withdrawal more than 3 years ago, or 3) seizures for which no treatment has been required within the 3 years prior to enrollment

19. Asplenia, functional asplenia or any condition resulting in the absence or removal of the spleen

20. Guillain-Barré Syndrome

21. Psychiatric condition that precludes compliance with the protocol; past or present psychoses; disorder requiring lithium; or within 5 years prior to enrollment, a history of suicide plan or attempt

22. Any medical, psychiatric, or other condition that, in the judgment of the investigator, is a contraindication to protocol participation or impairs ability to give informed consent

4.2 STUDY SCHEDULES

The study schema is shown in Table 4.1 and the study schedules that apply to study Groups are in **Appendix III**. Schedule A applies to Groups 1 and 2, while Schedule B applies to Groups 3, 4, 5 and 6. After Day 0, deviations from the visit windows in completing study visits are discouraged and will be recorded as protocol deviations, but are permitted, at the discretion of the Investigator of Record (IoR) or designee in the interest of obtaining subject safety and immunogenicity evaluations following study vaccinations. If it is in the best interests of the subject to receive the boost injection earlier than the preferred window due to influenza in the community, the IoR in consultation with the IND Sponsor may choose to do so.

In Groups 3, 4, 5 and 6 the 2013/14 TIV injections for all subjects are expected to be completed in the interval from when the product becomes available to sites in August 2013 through October 31, 2013. In this way the vaccinations will be completed before circulation of influenza in the community is projected to begin for the 2013/14 influenza season. Subjects who are not available

to receive their 2013/14 TIV prior to October 31, 2013 may receive it later and this will not be recorded as a protocol violation. Actual boost intervals will be calculated.

The follow-up visits to the boost should be scheduled relative to the actual date of the vaccination.

During or following any visit, if there is any concern about the well-being of the subject, the clinical study site will conduct appropriate medical evaluations by history, physical, laboratory or other indicated testing.

4.2.1 Pre-enrollment

Potential study subjects who verbally agree to discuss their medical history may complete an interview questionnaire by telephone or in person that covers protocol inclusion and exclusion criteria that are based on key self-reported history information to identify potential study volunteers.

4.2.2 Screening Visit(s)

Screening for this study may be completed through a general screening protocol after signing consent to be screened or screening consent may be incorporated into a VRC 703-specific consent form. A screening segment for the study will be included in data collection to provide information on reasons for non-enrollment. Evaluations will be done according to eligibility criteria and clinical assessment at screening. Screening evaluations for specific eligibility criteria must be completed within the time interval specified prior to enrollment, but may be repeated as needed to confirm eligibility. Storage samples of peripheral blood mononuclear cells (PBMCs) and serum collected during screening may be used for assay validation and site proficiency testing. As part of the screening process, an Assessment of Understanding (AoU) should be completed and incorrect answers will be explained to the subject.

4.2.3 Enrollment Visit

For all groups, enrollment is defined as the day of first study injection and is designated Study Day 0. Pregnancy test results for women of reproductive potential must be confirmed as negative on the day of enrollment prior to the study injection. Day 0 evaluations prior to the first injection are the baseline for subsequent safety and immunogenicity assessments. For the evaluations not performed on Day 0, then the baseline will be the screening evaluation. Previously screened subjects who arrive at the clinic on the scheduled day of enrollment with fever or evidence of an acute illness will not be enrolled, but may be rescheduled to enroll after resolution of the acute illness.

4.2.4 Administration of Injections

All Day 0 vaccinations will be administered open-label according to the randomized assignment. Injections of HA DNA will be administered into deltoid muscle in a 1 mL volume using a Biojector 2000® needle-free injection system (Biojector; Bioject Medical Technologies Inc., Portland, OR). The Biojector will be used as directed by the manufacturer. The Biojector uses sterile, single-use syringes for administration of volume up to 1 mL. The study agent is delivered under pressure by a compressed CO_2 gas cartridge that is stored inside the Biojector. Neither the material being injected nor injection site skin preparation requires deviation from standard procedures. The CO_2 does not come in contact with the injectate and the syringe design prevents any back splatter or contamination of the device by tissue from the subject.

The TIV injections will be administered ID or IM (according to the schedule assigned) as directed by the manufacturer's package insert.

For prime injections in Groups 5 and 6, the two injections administered on Day 0 will be administered in different arms. To assist with data monitoring, HA DNA will be administered in the left arm and the TIV (as indicated by the assigned schedule) will be administered in the right arm. In the case of skin changes (scars, tattoos, etc.) in the left deltoid region, at PI discretion, HA DNA may be administered in the right arm and TIV administered in the left arm.

Otherwise, when only a single injection is administered on a given day, it is recommended, but not required, that study injections be administered into the non-dominant arm. When choosing an arm for the injection, clinicians should consider whether there is an arm injury, local skin problem or significant tattoo that precludes administering the injection or will interfere with evaluating the arm after injection.

Targeted physical exam is performed on the day of boost vaccinations. A subject who arrives at the clinic with fever or evidence of an acute illness, which precludes administration of the vaccination, may be rescheduled within the allowed visit window.

Following study injection(s), subjects will be observed for a minimum of 30 minutes after injections of the investigational HA DNA vaccine and for a minimum of 15 minutes after any licensed TIV product injection. Blood pressure and pulse will be taken between 15 and 60 minutes post-injection. The injection site will be inspected for evidence of local reaction. Acute medical care will be provided to subjects for any immediate allergic reactions or other injury resulting from participation in this research study.

7-Day Diary Card and Follow-up: Subjects will be given a "Diary Card", to use as a memory aid, on which to record temperature and symptoms daily for 7 days following each injection. The site may use the electronic or written (paper) diary card as a source document or clinician notes obtained by telephone interview as the source of reactogenicity information recorded in the study database.

The solicited signs and symptoms on the diary card will include the parameters: unusually tired/feeling unwell, muscles aches (other than at injection site), headache, chills, nausea, and pain/tenderness at injection site(s). Subjects will also record the day's highest measured temperature and measurement of largest diameter for redness and swelling at injection site(s). For subjects who receive concurrent administration of DNA and a TIV product, pain/tenderness, redness, and swelling will be assessed separately for each injection site.

Subjects will be asked to contact the clinic if they have any concerning signs or symptoms. A clinic visit will be scheduled, if indicated, for the following: rash, urticaria, fever of 38.5˚C (Grade 2) or higher lasting greater than 24 hours, or significant impairment in the activities of daily living (ADL). Additionally, other clinical concerns may prompt a study visit based on the judgment of a study clinician.

4.2.5 Blood Sample Collection

At intervals throughout the study, blood will be drawn for safety and immunologic assays. Blood will be drawn from the arm veins of subjects by standard phlebotomy procedures.

4.2.6 Concomitant Medications and Procedures

Current concomitant medications are recorded in the study database at enrollment. Concomitant medications will be updated in the study database if there is an occurrence of an adverse event that requires expedited reporting. Whether or not any medications were taken during the 7-day period following vaccination will be documented. Treatment for influenza with antiviral drugs will be recorded on an Influenza Endpoint Case Report Form. Sites should work with study subjects with regard to the timing of FDA-approved vaccines such that, when possible, they should be scheduled at least 14 days before or at least 28 days after a study vaccination. Receipt of a licensed vaccine at any time is not a protocol violation; receipt of seasonal influenza vaccine outside the study will be discouraged and recorded as a protocol violation. Vaccinations received during study participation will be recorded in the study database. Otherwise, a record of concomitant medication changes throughout the study will not be recorded in the study database.

4.3 Criteria for Beginning Randomization into Groups 3-6

The unexpected amendment of the protocol in October 2012 due to uncertainty about the availability of the originally planned Novartis AGRIFLU and FLUAD products requires that each participating site receive an IRB approval before beginning enrollment into these groups. Sites are encouraged to seek an expedited approval given that the Sanofi Pasteur TIV products to be used are commercially available and licensed for use in adults in the U.S. It is in keeping with good public health recommendations to administer these influenza vaccine products to adults who have not yet been vaccinated.

4.4 Criteria for Discontinuing Study Injections or Protocol Participation

In general, subjects who receive the Day 0 study injection will continue to be followed, whenever possible, as indicated on the schedule of evaluations.

4.4.1 Discontinuation of Study Injections

For those who begin a schedule, discontinuation of the injection schedule may occur for the following reasons:

1. Subject declines to receive the second study injection.
2. Circumstances have arisen that constitute a contraindication to administering the vaccine as indicated by the package insert for licensed TIV or as based on the clinical judgment of the IoR or Protocol Chair.
3. The Site IoR assesses that it is not in the best interest of the subject to continue on the vaccination schedule.

Pregnancy is not a contraindication to the licensed TIV, but research blood draws will be adjusted as indicated in **Appendix III**.

The TIV injection can be offered by the site to a subject who becomes pregnant as a standard of care.

4.4.2 Discontinuation from Protocol Participation

A subject may be discontinued from protocol participation for the following reasons:

1. Subject decides to discontinue participation.
2. Subject develops a medical condition that is a contraindication to continuing study participation.
3. The Sponsor or regulatory authority stops the protocol.
4. The Site Investigator of Record (IoR) assesses that it is not in the best interest of the subject to continue participation in the study or that the subject's compliance with the study is not sufficient.

4.5 Criteria for Pausing the Study

The Protocol Chair and UNI-CPSC Medical Monitor will closely monitor and analyze study data as they become available. The VRC Medical Officer will provide an independent review of adverse events on a regular basis. The administration of study injections and new enrollments will be paused according to the following criteria:

- One (or more) subject experience a Serious Adverse Event (SAE) assessed as related to study vaccination.
- One (or more) subject experiences a Grade 4 or Grade 5 adverse event that is assessed as related to a study vaccine.
- Six (or more) subjects in any treatment group experience a similar Grade 3 adverse event assessed as related to study vaccination.

Investigator attributions of relationship to vaccination are not collected for the 7-day solicited reactogenicity parameters, however, any Grade 3 reactogenicity reported in the 7 days after vaccination will be assumed to have some possible relationship to the vaccination and will count towards a pause.

The study injections and enrollments would resume only if review of the adverse event(s) that caused the pause resulted in a recommendation to permit further study injections and study enrollments. The Protocol Safety Review Team (PSRT) will conduct the review and make a decision to resume or close the study. Changes in study status will be communicated to the study sites promptly by the UNI-CPSC.

5 SAFETY AND ADVERSE EVENTS

5.1 Adverse Events

5.1.1 Adverse Event (AE) Definition

An AE is any untoward or unfavorable medical occurrence in a human subject, including any abnormal sign (e.g., abnormal physical exam or laboratory finding), symptom, or disease temporally associated with the use of study treatment, whether or not considered related to the study treatment.

5.1.2 Adverse Event Reporting in the Study Database

The following guidelines will be used to determine whether or not an adverse event is recorded in the study database.

Each adverse event will be graded according to the table for grading severity of adverse events (see **Appendix IV**).

Solicited adverse events will be recorded in the study database separately with data collection for 7 days after both the first vaccination and the second injection as detailed in Section 4.2.4; without the collection of attribution assessments. All unsolicited AEs will be recorded in the study database from receipt of first study injection through 28 days after each study injection. At other time periods between injections and after the second injection, only SAEs (as detailed in **Section 5.2**), new chronic medical conditions and influenza or influenza-like illness will be recorded through the last study visit. However, cases of influenza or influenza-like illness will be recorded on an influenza endpoints form rather than on an adverse events form.

Any adverse events associated with licensed TIV that meet the criteria for reporting under the Vaccine Adverse Events Reporting System (VAERS) system, are the responsibility of the IoR to report in accordance with the guidance on reportable events available at the website http://vaers.hhs.gov/professionals/index). Refer to the brochure with description and guidance to the VAERS system, which is co-sponsored by the Centers for Disease Control and Prevention (CDC) and the Food and Drug Administration (FDA): http://vaers.hhs.gov/resources/VAERS_Brochure.pdf.

5.2 Serious Adverse Events

5.2.1 Serious Adverse Event Definition

The term "Serious Adverse Event" (SAE) is defined in the 21 CFR 312.32 in terms of the following outcomes: Death, a life-threatening adverse event, inpatient hospitalization or prolongation of existing hospitalization, a persistent or significant incapacity or substantial disruption of the ability to conduct normal life functions, or a congenital anomaly/birth defect. Important medical events that may not result in death, be life-threatening, or require hospitalization may be considered serious when, based upon appropriate medical judgment, they may jeopardize the patient or subject and may require medical or surgical intervention to prevent one of the outcomes listed in this definition. Examples of such medical events include allergic bronchospasm requiring intensive treatment in an emergency room or at home, blood dyscrasias or convulsions that do not result in inpatient hospitalization, or the development of drug dependency or drug abuse."

An SAE will be considered "life-threatening" if, in the view of either the investigator or sponsor, its occurrence places the patient or subject at immediate risk of death. It does not include an adverse event or suspected adverse reaction that, had it occurred in a more severe form, might have caused death.

5.2.2 Reporting Serious Adverse Events to the IND Sponsor

Adverse events that meet Serious Adverse Event (SAE) Reporting Requirements must be reported and submitted by the clinical site on an expedited basis to the IND sponsor, VRC/NIAID/NIH, according to sponsor guidelines as follows:

- death
- life-threatening
- results in persistent or significant disability/incapacity

- requires unplanned inpatient hospitalization or prolongation of existing hospitalization
- is a congenital anomaly/birth defect in the offspring of a study subject
- is an important medical event that may jeopardize the patient or may require intervention to prevent one of the other outcomes listed above.

In addition, any event, regardless of severity, which in the judgment of a site investigator represents a serious adverse event, may be reported on an expedited basis.

A site investigator will communicate an initial SAE report within 24 hours of site awareness of occurrence to VRC (IND Sponsor) through the communication methods provided by the Data Coordinating Center, EMMES Corporation (Rockville, MD).

Any SAE entered into the study database will generate automatic email notification to the UNI-CPSC Medical Monitor and VRC Medical Officer. This or a written report by the study site sent to the attention of the UNI-CPSC Medical Monitor (Email: uniflu@emmes.com or Fax: 301-576-3558) must be submitted within 3 working days in order for the sponsor to comply with regulations mandating sponsor notification of specified SAEs to the FDA within 7 calendar days.

The investigator must submit additional information as it becomes available.

5.2.3 IND Sponsor Reporting to the FDA

It is the responsibility of the IND Sponsor to make the determination of which SAEs are "serious and unexpected suspected adverse reactions" (SUSARs) as defined in 21 CFR 312.32.

- *Suspected adverse reaction* means any adverse event for which there is a reasonable possibility that the drug caused the adverse event.
- *Unexpected Adverse Event* means an AE that is not listed in the Investigator's Brochure or is not listed at the specificity or severity that has been observed.

All SUSARs, as determined by the IND Sponsor, will be reported to the FDA as IND Safety Reports and IND Safety Reports will be provided to all participating Investigators by the UNI-CPSC.

The IND Sponsor will also submit an IND Annual Report of the progress of the investigation to the FDA as defined in 21 CFR 312.33.

5.3 Reporting to Site IRBs

Each site IoR is responsible for reporting adverse events to the site IRB in accordance with their IRB's requirements for expedited reporting and continuing review reporting.

Site-specific data reports will be made available to facilitate this continuing review reporting. If there is an IND Safety Report, these will be provided to all sites with instruction as to whether or not any actions need to be taken, such as amendment of consent. Investigators must maintain documentation of compliance with actions required for IND safety reports.

5.4 Safety Monitoring

The Protocol Safety Review Team (PSRT) (see **Section 8.8**), will have the primary responsibility for the real-time oversight of safety data, SAE reviews and study pause reviews.

6 STATISTICAL CONSIDERATIONS AND SAMPLE ANALYSIS

6.1 OVERVIEW

This study is a multi-center trial to assess the safety and immunogenicity of prime-boost vaccination regimens with the investigational plasmid HA DNA vaccine directed towards the 2012/13 influenza vaccine strains as a prime followed 14 weeks later by 2012/13 influenza TIV ID or TIV IM (Fluzone® brand) as compared to the respective single injection of TIV ID or TIV IM. The study will also evaluate the safety and immunogenicity of vaccination regimens with concurrent administration of 2012/13 HA DNA vaccine and TIV ID or TIV IM followed by administration of the subsequent season (2013/14) TIV ID or TIV IM 40 weeks later.

6.2 OBJECTIVES

The primary safety objectives relate to the safety and tolerability of each vaccination regimen. The primary immunogenicity objective is to compare the HAI response from the same season prime-boost regimens (2012/13 HA DNA prime and TIV ID or TIV IM boost with a 14 week interval) measured 3 weeks following the boost injection, with the HAI response at three weeks following TIV ID or TIV IM alone. The secondary immunogenicity objectives are to evaluate the HAI and neutralizing antibodies for all vaccine regimens three weeks after each injection.

6.3 ENDPOINTS

6.3.1 Primary Endpoints: Safety

Assessment of product safety will include clinical observation and monitoring of hematological parameters. Safety will be closely monitored after each injection and evaluated by clinical visits through 24 weeks after the boost injection on each schedule. The following safety endpoints will be assessed for all Study Groups:

- Occurrence of solicited local reactogenicity signs and symptoms for 7 days following each vaccination
- Occurrence of solicited systemic reactogenicity signs and symptoms for 7 days following each vaccination
- Mean change from baseline for safety laboratory measures at week 3
- Occurrence of adverse events of all severities through 28 days after the prime injection and through 28 days after TIV ID or TIV IM boost
- Occurrence of serious adverse events through last study visit
- Occurrence of Influenza or influenza-like illness events through last study visit

6.3.2 Primary Endpoint: Immune Response

For all immunogenicity endpoints, seroconversion is defined as the proportion of subjects with either a pre-vaccination strain-specific HAI titer <1:10 and a post-vaccination HAI titer ≥1:40 or a pre-vaccination HAI titer ≥1:40 and a minimum four-fold rise in post-vaccination HAI antibody titer.

- Seroconversion for each of the 2012/13 influenza vaccine strains at 3 weeks after completion of the HA DNA prime-boost regimens (Group 1 and Group 2), or 3 weeks after the TIV ID or TIV IM prime (Group 3 and Group 4)

- Geometric Mean HAI Titer for each of the 2012/13 influenza vaccine strains at 3 weeks after completion of the HA DNA prime-boost regimens (Group 1 and Group 2), or 3 weeks after the TIV ID or TIV IM prime (Group 3 and Group 4)

6.3.3 Secondary Endpoints: Immune Responses

- Seroconversion for each of the 2012/13 and 2013/14 influenza vaccine strains at three weeks after each study injection, for all study groups.

- Geometric Mean HAI Titer for each of the 2012/13 and 2013/14 influenza vaccine strains before each injection and at 3 weeks after each study injection, for all study groups.

- Proportion of subjects with four-fold rise in 2012/13 and 2013/14 influenza vaccine strain-specific H1, H3, and B neutralizing antibodies before the boost injection and at 3 weeks after each study injection, for all study groups.

- Geometric Mean neutralization titer of 2012/13 and 2013/14 influenza vaccine strain-specific H1, H3, and B neutralizing antibodies before vaccination and at 3 weeks after each study injection, for all study groups.

6.3.4 Exploratory Endpoints: Immune Responses

- Proportion of subjects with anti-stem antibodies pre-vaccination and at 3 weeks and 24 weeks after the boost vaccination for each schedule.

- Proportion of subjects with positive HA-specific T-cell responses (as measured by ICS assay or ELISpot assay) before each vaccination, 3 weeks after each vaccination and 24 weeks after the boost for all study groups.

Other exploratory assays to evaluate antibody or T cell responses over the time course of the study may also be performed.

6.4 SAMPLE SIZE AND ACCRUAL

The study is planned to enroll a total of 330 healthy adults stratified by age groups (18-50 and 51-70) to receive one of six vaccine regimens defined in **Table 4-1**. The first 110 subjects enrolled will be randomized with equal allocation to receive 2012/13 HA DNA prime with 2012/13 TIV ID boost (Group 1) or 2012/13 HA DNA prime with 2012/13 TIV IM boost (Group 2). The remaining 220 subjects will be randomized with equal allocation to receive 2012/13 TIV ID with 2013/14 TIV ID boost (Group 3); 2012/13 TIV IM with 2013/14 TIV IM boost (Group 4); concurrent administration of 2012/13 HA DNA and TIV ID with 2013/14 TIV ID boost (Group 5); or concurrent administration of 2012/13 HA DNA and TIV IM with 2013/14 TIV IM boost (Group 6).

Enrollments may occur rapidly at more than one site. The EMMES Corporation will carefully monitor study enrollment and notify all sites and the VRC Protocol Chair when the completion of enrollment is near in order to end recruitment and to plan how to fairly accommodate, to the degree possible, the enrollment of eligible volunteers that have already been recruited. The decision to stop study enrollments will be made by the VRC Protocol Chair.

This study is not designed to test a formal null hypothesis for superiority or non-inferiority. This section does provide an indication of the power available to observe specific events (e.g., particular adverse events) assuming a range of underlying "true" rates, the precision available in estimating immune response rates, and the power to detect differences in response rates between Study Groups. While drop-out is expected to be low, power calculations assuming approximately 10% drop-out (N=50/per group) are also presented.

6.4.1 Power Calculations for Evaluation of Safety

Sample size calculations for primary safety objectives are expressed in terms of the ability to detect safety or reactogenicity events in each study group (N = 55). The ability of the study to identify safety events will be expressed in terms of the probability of observing a certain number of events. Useful values are the minimum true event rate such that the probability of observing at least one event is at least 90% and the maximum true event rate such that the probability of not observing any event is at least 90%. Within each Study Group of 55 subjects, there is over 90% chance to observe at least 1 event if the true rate is at least 0.05 (1 of 20) and over 90% chance to observe no events if the true rate is less than 0.002 (1 of 500).

Probabilities of not observing any events or observing more than 1 event are presented in **Table 6-1** for a range of possible true event rates. These calculations provide a more complete picture of the sensitivity of this study design to identify potential safety concerns with the vaccine.

Table 6-1: Probability of Observing Events for Different Safety and Immunogenicity Scenarios within a Study Group (n = 55)

	**N=55**		**N=50**	
**True Event Rate**	**Pr (0 events)**	**Pr (>1 event)**	**Pr (0 events)**	**Pr (>1 event)**
0.003<br>(1 in 300)	0.85	0.01	0.86	0.01
0.01<br>(1 in 100)	0.58	0.11	0.61	0.091
0.05<br>(1 in 20)	0.06	0.77	0.08	0.72
0.1<br>(1 in 10)	0	0.98	0.01	0.97
0.2<br>(1 in 5)	0	1	0	1

Table 6-2 gives the upper and lower bounds for 95% exact (Clopper-Pearson) binomial confidence intervals of the true event rates for a range of possible numbers of observed events. If none of the 55 subjects in a Study Group experience the event of interest, the 95% exact 2-sided upper confidence bound for the event rate is 0.07.

Table 6-2: 95% Exact Confidence Intervals for the True Rate for a Range of Possible Observed Rates within a Study Group (N=55)

Observed Rate	N=55		N=50	
	Lower bound	Upper Bound	Lower bound	Upper bound
0	0	0.07	0	0.07
0.10	0.04	0.21	0.03	0.22
0.20	0.10	0.33	0.10	0.34
0.30	0.18	0.44	0.18	0.45
0.40	0.27	0.54	0.26	0.55
0.50	0.36	0.64	0.36	0.65
0.60	0.46	0.73	0.45	0.74
0.70	0.56	0.82	0.55	0.82
0.80	0.67	0.67	0.66	0.90
0.90	0.78	0.79	0.78	0.97
1	0.94	1.0	0.93	1.00

6.4.2 Power Calculations for Evaluation of Immune Responses

The tables presented in the previous section for evaluation of safety response, are also applicable to the evaluation of immune response (e.g., seroconversion). **Table 6-1** gives the probabilities of observing no subjects with immune response or at least 2 subjects with immune response over a range of underlying response rates. **Table 6-2** gives the exact 95% confidence intervals over a range of observed response rates for each Study Group.

6.4.3 Power Calculations for Primary Immunogenicity Comparisons

The primary immunogenicity objective is to compare the frequency and magnitude of the 2012/13 influenza vaccine strain-specific HAI response at 3 weeks after the TIV ID boost in Group 1 to 3 weeks after the TIV ID prime in Group 3, as well as to compare the same responses at 3 weeks after the TIV IM boost in Group 2 to 3 weeks after the TIV IM prime in Group 4. Each of these comparisons will be made for the 3 strains in the 2012/13 vaccines, so a total of six comparisons will be made for the two primary immunogenicity endpoints, seroconversion and GMT.

Table 6-3 gives the power to detect a difference in the seroconversion using Fisher's exact test with Type I error of 5%over a range of possible seroconversion rates. **Table 6-4** presents the minimum difference in seroconversion rates that can be detected with 55 or 50 subjects in each Study Group using Fisher's exact tests with 80% power and 5% Type I error rate.

Table 6-3: Power to Detect Difference in Response Rates between Two Study Groups by Fisher's Exact Test

**Seroconversion Rate**		**Power (%)**	
**Comparator Group**	**Prime-Boost Group**	N=55	N=50
0.4	0.6	46	46
	0.7	85	83
	0.8	99	98
	0.9	>99	>99
0.5	0.7	49	46
	0.8	89	85
	0.9	>99	99
0.6	0.8	56	51
	0.9	95	93
0.7	0.9	68	64

Table 6-4: Minimum Detectable Difference in Response Rates with 80% Power

**Comparator Group**	**Minimum Detectable Difference**	
	**N=55**	**N=50**
0.40	0.28	0.29
0.50	0.27	0.28
0.60	0.25	0.26
0.70	0.22	0.23
0.80	0.18	0.19

The calculations in this section are presented for the primary immunogenicity comparisons, but are also applicable to comparisons of seroconversion between any two Study Groups, as well as comparisons of proportion of subjects with four-fold increase in neutralizing antibodies, positive anti-stem antibodies, or positive HA specific T-Cell response.

6.5 Statistical Analysis

Study enrollment is defined in this protocol as being randomized and receiving the first study vaccination. All enrolled subjects will receive at least one vaccination and therefore will provide some safety data, and be included in the safety analysis.

All statistical analyses will be performed using Statistical Analysis System (SAS) or R statistical software.

A significance level of alpha = 0.05 will be used for all analyses; no formal multiple comparison adjustments will be employed for safety endpoints or secondary endpoints.

6.5.1 Analysis Variables

The analysis variables consist of baseline characteristics, safety, reactogenicity, and immunogenicity variables.

6.5.2 Baseline Characteristics

Baseline characteristics including demographics and laboratory measurements will be summarized using descriptive statistics.

6.5.3 Safety Analysis

Reactogenicity: The number and percentage of subjects experiencing each type of reactogenicity sign or symptom will be tabulated by severity. For a given sign or symptom, each subject's reactogenicity will be counted once under the maximum severity for all assessments.

Adverse Events: Adverse Events (AEs) will be coded into Medical Dictionary for Regulatory Activities (MedDRA) preferred terms. The number and percentage of subjects experiencing each specific adverse event will be tabulated by severity and relationship to study product. For the calculations in these tables, each subject's adverse event will be counted once under the maximum severity or strongest recorded causal relationship to study product.

A complete listing of AEs for each subject will provide details including severity, relationship to study product, onset, duration and outcome.

Safety Laboratory Values: Safety laboratory values will be summarized as the mean change from baseline along with 95% confidence interval at each timepoint measured in the study. Boxplots of safety laboratory values will be generated for baseline values and for values measured during the course of the study.

6.5.4 Analysis of Immune Responses

For the primary analysis for immunogenicity all data from enrolled subjects will be used and subjects will be analyzed according to the study product(s) received.

The frequency of immune response will be analyzed by tabulating the seroconversion (proportion of subjects with either a pre-vaccination strain-specific HAI titer <1:10 and a post-vaccination HAI titer ≥1:40 or a pre-vaccination HAI titer ≥1:40 and a minimum four-fold rise in post-vaccination HAI antibody titer) and exact 95% confidence intervals for each study group. The magnitude of immune response will be analyzed by tabulating the geometric mean titer for

each study group. Neutralizing antibodies will be summarized by calculating GMTs and proportion of subjects with a four-fold rise in titer. For immune response at time points after the boost injection seroconversion and fold-rise will be calculated relative to baseline (Day 0), and relative level on the day of the boost (Visit 5). The immune response may also be summarized separately for each age subgroup. Fisher's exact tests will be used to compare seroconversion between any two study groups.

Missing responses will be assumed to be missing at random, i.e., conditional on the observed data the missingness is independent of the unobserved responses, no imputations for missing data will be made.

6.5.5 Interim Analyses

Safety Reviews: The protocol safety review team will review safety data routinely throughout the study. The study will utilize both electronic database features and reviews by designated safety review personnel to identify in a timely manner if any of the safety pause rules of the study are met.

Immunogenicity Review: The analyses of immunogenicity may be performed when the HAI assays of samples collected at 3 weeks after the TIV ID or TIV IM injections in Groups 1 and 2 and the HAI assays of samples collected at 3 weeks after the prime TIV ID or TIV IM injections in Groups 3-6 have been completed for the study. This may occur prior to completion of safety follow-up visits or collection of data for secondary and exploratory immunogenicity endpoints. Such an analysis would constitute the final analysis for the primary immunogenicity endpoint, so sample size adjustments are not required. Reports providing results by study group may be provided to VRC solely for the purpose of informing decisions related to future trials in a timely manner. The results should in no way influence the conduct of the VRC 703 trial in terms of early termination or later safety or immunogenicity endpoint assessments. Analyses secondary and exploratory immunogenicity assays may also be performed as data are available.

6.5.6 Randomization of Treatment Assignments

Enrollment and randomization into Group 1 and Group 2 are targeted to begin in August 2012, enrollment and randomization into Groups 3, 4, 5 and 6 are targeted to begin in November 2012. Enrollment and randomizations will be done online using the enrollment module of The EMMES Corporations Internet Data Entry System (IDES). The randomization code will be prepared by statisticians at The EMMES Corporation and included in the enrollment module for the trial. To decrease the potential for subject dropouts during the period between randomization and initial vaccination, the electronic data system will assign each subject a randomized study group assignment after the eligibility to begin Day 0 study injections has been entered into the system.

The first 110 subjects enrolled will be randomized with equal allocation to Group 1 or Group 2 stratified by site and age (18-50, 51-70). All subjects ages 65-70 years enrolled prior to the protocol amendment will receive TIV IM boosts, regardless of the Group to which they were randomized. The subsequent 220 subjects enrolled will be randomized to Groups 3-6. Randomization will be stratified by clinical site and age. Within the 18-50 and 51-64 age strata subjects will be randomized with equal allocation to Groups 3- 6, and within the 65-70 age strata subjects will be randomized with equal allocation to Group 4 or Group 6 only through November

16 to accommodate the already screened volunteers; thereafter, enrollments will be limited to subjects who will be no older than 64 years at their 2013/14 vaccination timepoint. Manual back-up procedures and instructions will be provided for use in the event that the site temporarily loses access to the Internet or the online enrollment system is unavailable to a study site.

7 PHARMACY AND VACCINE ADMINISTRATION PROCEDURES

The vaccination schedules are shown in **Table 4.1 in Section 4.0**.

7.1 STUDY AGENTS

This study includes investigational vaccines and licensed seasonal TIV vaccine as follows:

- VRC-FLUDNA063-00-VP 4 mg/mL (2012/13 HA DNA vaccine)
- 2012/13 TIV ID (Fluzone®)
- 2013/14 TIV ID (Fluzone®)
- Licensed seasonal 2012/13 TIV IM (Fluzone®)
- Licensed seasonal 2013/14 TIV IM (Fluzone®)

The HA DNA vaccine is manufactured by VRC/NIAID/Vaccine Pilot Plant (VPP) in Frederick, Maryland. The TIV ID and TIV IM are manufactured by Sanofi Pasteur and will be obtained from commercially available supplies of licensed vaccine. The VPP will serve as the repository for study investigational vaccine supplies prior to their distribution to study sites. At each site, the study vaccines will be maintained in a controlled and secure location in a manner appropriate for investigational products.

7.2 STUDY AGENT PRESENTATION AND STORAGE

7.2.1 Study Agent Labels

At the time of delivery of the study agents to the pharmacy, the labels for VRC-FLUDNA063-00-VP (DNA vaccine) will have specific product information (e.g., part number, lot number, fill volume, storage temperature) included on the product vial labels. The labels will contain an Investigational Use Statement ("Caution: New Drug – Limited by Federal Law to Investigational Use") and manufacturer information.

7.2.2 Study Agent Storage

Temperature excursions that are outside of the normal allowance for the storage device in which each type of product is kept will be reported to the study sponsor via the study coordinating center (The EMMES Corporation). The excursion must be evaluated and investigated and action must be taken to restore and maintain the desired temperature limits. The site IoR is ultimately responsible for notification of the sponsor, but may delegate this responsibility to a qualified staff member. Pending the outcome of the investigation, the site will be informed if continued clinical use of the product is acceptable.

VRC-FLUDNA063-00-VP: Upon release by VRC/NIAID/NIH, the DNA vaccine vials will be shipped within the recommended temperature range using appropriate shipping configurations, to the study pharmacist, and will be stored until use at -45°C to -10°C in a qualified, continuously monitored, temperature-controlled freezer.

TIV (Fluzone®): The licensed TIV for IM and ID administration will be stored according to the package insert instructions and released by the pharmacist to the designated clinical staff for administration to study subjects.

7.3 PREPARATION OF STUDY AGENT FOR INJECTION

This section describes how the site pharmacist or designee will prepare the DNA vaccine and TIV injections. Clinician instructions on how to select an arm and administer the injection are in **Section 4.2.4**.

7.3.1 Preparation of VRC-FLUDNA063-00-VP

The DNA vaccine is supplied as a 2 mL glass vial containing a clear colorless isotonic sterile solution. Each vial contains 20% over the amount to be injected in cGMP grade phosphate-buffered saline. Vials are intended for single use only, and thus do not contain a preservative. They should not be refrozen after thawing. Each vial (4 mg/mL) contains a volume of 1.2 mL (4.8 mg).

Refer to the group assignment for the study subject. For subjects to whom the DNA vaccine is to be administered, remove a vial of the DNA vaccine 4 mg/mL from the freezer. Allow the vial to equilibrate to room temperature (15 to 30º C). Swirl the contents gently. Using aseptic technique, withdraw 1 mL of the DNA vaccine from the vial into the Biojector syringe, remove air bubbles and cap the syringe. The pharmacy will label the syringe prior to delivery to the clinic with the subject identifier and the date and time allowance for administration; the label will include the product type information.

One 1 mL injection of the 4 mg/mL preparation will be administered for each 4 mg dose of DNA vaccine. A dose of vaccine will be prepared in the pharmacy at the site, and the prepared Biojector syringe labeled with the subject identifier will be delivered to the clinic for administration. The pharmacy/designated site personnel will also label with information about date and time after which the preparation may not be used. The injection must be administered within 8 hours after removing the vial from the freezer.

7.3.2 Preparation of TIV ID and TIV IM

The TIV ID and TIV IM are administered in accordance with the package insert for each product, respectively.

7.4 STUDY AGENT ACCOUNTABILITY

7.4.1 Documentation

Each study site will be responsible for maintaining an accurate record of the codes, inventory, and an accountability record of all vaccine supplies for this study at their site.

7.4.2 Disposition

The empty syringes, vials (HA DNA) and the unused portion of a vial will be discarded in a biohazard containment bag that will be incinerated or autoclaved. Any unopened vials and syringes that remain at the end of the study will be discarded at the discretion of the VRC in accordance with policies that apply to investigational agents. Partially used vials or expired

prepared doses cannot be administered to other subjects nor used for *in vitro* experimental studies and will be discarded as indicated above.

8 HUMAN SUBJECTS PROTECTION

8.1 Institutional Review Board

A copy of the protocol, proposed informed consent and any proposed advertising material will be submitted to the site IRB for review and approval.

The Site IoR will submit and, where necessary, obtain approval from the IRB for subsequent protocol amendments and changes to the informed consent document. The Site IoR is responsible for ensuring proper IRB notification of deviations from the protocol or serious adverse events occurring at the site and other adverse event reports received from the VRC, NIAID, in accordance with the protocol and local IRB policies. The IoR will be responsible for obtaining annual IRB approval/renewal throughout the duration of the protocol. Documentation of the IRB approval and FWA number will be provided for the Sponsor's records.

8.2 Subject Recruitment and Enrollment

Subjects for this study will be recruited by the sites in accordance their site IRB standard for recruitment practices. Effort will be made to include women and minorities in proportions similar to that of the community from which they are recruited.

8.3 Informed Consent

The provided template informed consent (**Appendix I)** will be used to guide development of the site-specific consent forms. Only an IRB-approved consent form will be used to consent subjects for participation in the study. Changes in the informed consent template by the site should be approved by a UNI-CPSC Regulatory Reviewer before submission to the site IRB. The written informed consent documents will be prepared in the language(s) of the potential subject population. Before a subject's participation in the protocol, it is the investigator's responsibility to ensure that written informed consent is obtained from the subject after adequate explanation of the aims, methods, anticipated benefits, and potential hazards of the protocol.

The acquisition of informed consent should be documented in the subject's records, as required by 45 CFR 46.117, and the informed consent form should be signed and personally dated by the subject and by the person who conducted the informed consent discussion. An original signed informed consent form should be retained by the site and a signed copy of the consent form should be provided to the subject.

8.4 Subject Confidentiality

The investigators at each site must ensure that the subject's anonymity is maintained. Subjects will not be identified in any reports of this study. All records will be kept confidential to the extent provided by federal, state and local law. Medical records will be made available for review when required by authorized agencies and regulatory authorities only under the guidelines set by the Federal Privacy Act. Direct access includes examining, analyzing, verifying, and reproducing any records and reports that are important to the evaluation of the

study. The above named representatives will review study-related records without violating the confidentiality of the subjects. Stored study research samples will be labeled by a code (such as a number) that only the site clinical study team can link to the subject. The requirement to maintain subject confidentiality and inform subjects about review of study-related records is included in the study informed consent documents.

8.5 RISKS AND BENEFITS

8.5.1 Risks of Blood Collections:

The blood collection procedures are common in routine medical practice. The risks of blood sample collection are minimal and consist of mild discomfort at the sample collection site. The procedure may cause mild pain, bruising, fainting, and, rarely, infection at the site where the blood is taken.

8.5.2 Risks of the DNA Vaccine:

This is one of the two studies in humans of the trivalent seasonal HA DNA vaccine, VRC-FLUDNA063-00-VP. The H1 DNA plasmid previously has been administered alone and as part of another HA DNA vaccine. The risks noted are based on risks from the earlier DNA vaccine studies of similar influenza DNA vaccines, as well as risks of vaccines in general and results of previous studies with other investigational DNA vaccines.

Subjects may exhibit general signs and symptoms associated with administration of a vaccine injection, including fever, chills, rash, aches and pains, nausea, headache, dizziness and fatigue. These side effects, when they occur, are generally short term, mild to moderate severity, and usually do not require treatment.

In previous VRC DNA vaccine studies, placebo and vaccine recipients were noted to have occasional asymptomatic and self-limited changes in the laboratory tests routinely followed during the clinical trial. Urticaria has been reported as an infrequent adverse event possibly related to DNA vaccines.

Investigational DNA vaccines administered via Biojector have been associated with mild skin lesions (0.5-1.0 cm diameter) at the vaccination site. In these cases, a small scab formed within 1-2 weeks after immunization and came off after a few days. The skin healed without treatment within a few weeks. One skin biopsy was obtained on Day 6 post vaccination. It showed subcutaneous and dermal perivascular lymphocytic inflammation. There were rare eosinophils and rare giant cells noted, and the infiltrate was composed entirely of CD3 positive cells. It included both $CD4^+$ and $CD8^+$ T cells. The process appears to be primarily a subcutaneous inflammatory response to vaccination with cutaneous manifestations.

There may be other unknown side effects.

8.5.3 Risks of the Seasonal TIV:

Occasionally, adult recipients of seasonal influenza TIV may develop influenza-like reactions such as fever, hoarseness, sore, red or itchy eyes, cough, body aches, headache, itching, malaise, myalgia, and/or nausea. These reactions are usually greatest within the first 24 hours after vaccination and may last for 1 to 2 days. Some subjects may develop reactions at the site of vaccination (redness, swelling, pain, or tenderness). Analgesics (e.g., ibuprofen and acetaminophen) and rest will generally relieve or moderate these symptoms. These reactions

should go away in 1 to 4 days and not require additional treatment. The most common reactions reported for Fluzone were pain at the injection site (>50%), and headache and myalgia (>30%), and these reactions were generally mild and of limited duration.

Acute and potentially life-threatening allergic reactions are also possible. Since the vaccine may contain limited quantity of egg protein, this protein can induce immediate hypersensitivity reactions among person who have severe allergy. Allergic reactions include hives, angioedema, allergic asthma, and systemic anaphylaxis.

During the swine influenza vaccine campaign of 1976, about 1 per 100,000 vaccine recipients developed a paralytic illness called Guillain-Barré Syndrome. This has not been seen consistently with other influenza vaccines. Most persons who develop Guillain-Barré Syndrome recover completely.

There may be other unknown side effects.

8.5.4 Risks of the Seasonal TIV administered ID:

The risks of Fluzone® as described in Section 8.5.3 are the same for ID administration. The package insert for Fluzone® Intradermal notes that the most common reactions reported in clinical studies of TIV ID were erythema (>75%), swelling (>50%), pain (>50%), and pruritus (>40%). There is a higher frequency of local reactions with ID than with IM administration. The most common solicited reactions were headache, myalgia and malaise (>20%).

The TIV ID product was first approved by the FDA in May 2011. There may be other unknown side effects. Sites will provide the Vaccine Information Statement (VIS) for 2012/13 or 2013/14 Influenza Vaccine injections, respectively; the VIS includes a statement of risks to participating subjects.

8.5.5 Other Risks:

Women who are pregnant or nursing will be excluded from enrollment into the study. The effect of the investigational DNA vaccine on a fetus or nursing baby is unknown. The licensed TIV IM product is approved for administration during pregnancy, but the use of the TIV ID has not been established for administration in the U.S.

Women of reproductive potential will be required to agree to use birth control for sexual intercourse beginning 21 days prior to enrollment and continue through 3 weeks after the last study injection. Because this is a research study, women of reproductive potential will be asked to notify the site immediately upon learning of a pregnancy during this study and will be tested for pregnancy prior to administration of each study injection. The amount of blood drawn will be reduced. The subject will be contacted to ask about the outcome of a pregnancy that begins during the study.

It is possible that the standard medical tests performed as part of this research protocol will result in new diagnoses. Depending upon the medical findings and consequences of being provided with the new medical information about health status, the study subject may view this aspect of study participation as either a risk or a benefit. Any such information will be shared and discussed with the subject and, if requested by the subject, will be forwarded to the subject's primary health care provider for further workup and management.

8.5.6 Benefits:

Study participants may have no direct benefit from participation in this study. This protocol is not designed to provide treatment for any condition. Receiving TIV ID or TIV IM may prevent influenza. The DNA vaccine alone is not expected to provide protection from influenza. Receiving the DNA vaccine may change the overall response to the TIV vaccines.

8.6 Plan for Use and Storage of Biological Samples

To be eligible for this protocol, subjects must be willing to allow stored specimens to be used in the future for studying infectious diseases, immune function, vaccine responses and other medical conditions, and must also be willing to have genetic tests, including HLA typing performed. If tests performed at a study site show evidence of any acute or chronic condition, subjects will be informed of the results and advised to seek appropriate medical care for the condition. In general, testing performed at a research laboratory is not for diagnostic purposes and results will not be available to the study site or study subject.

Intended Use of the Samples/Specimens/Data:

Samples, specimens and data collected under this protocol may be used to study infectious diseases such as influenza, immune function, vaccine responses, genetic factors in immune responses, other medical conditions and for research assay validation.

How Samples, Specimens and Data from Sample Use Will Be Stored:

All of the stored study research samples will be labeled by a code (such as a number) that only the study site can link to the subject. Samples will be stored in secure facilities with controlled access at the sites, a central repository maintained by NIH or at central laboratories associated with the study. Samples collected for research may be transferred for testing to the approved collaborators. Data will be kept secure. Only approved investigators or their designees will have access to samples and data. The NIAID Vaccine Immune T-Cell and Antibody Laboratory (NVITAL) in Gaithersburg, MD, under the direction of the VRC, NIAID, NIH (Bethesda, MD) and research labs at or contracted to the VRC or The EMMES Corporation will be the involved in conducting assays with stored samples.

In the future, other investigators (both at NIH and outside) may wish to study these samples and/or data. Regulatory approval through the proper human subjects protection agency will be sought prior to any sharing of samples that constitutes human subject research. The research use of stored, unlinked or unidentified samples may be exempt from the need for IRB review and approval. When appropriate, exemption may be obtained through the proper regulatory procedures.

8.7 Compensation

Compensation for study visits and procedures will be provided to offset the time and inconvenience of participation. Subjects will be compensated in accordance with the site-specific IRB approval.

8.8 Safety Monitoring

8.8.1 Protocol Safety Review Team

Each site IoR is responsible for ensuring daily review of the site's clinical safety data as it

becomes available. The Protocol Safety Review Team (PSRT) includes the Protocol Chair and/or Protocol Co-Chair, IND Sponsor Medical Officer, the UNI-CPSC Medical Monitor and each site IoR or designee. The PSRT will review the summary study safety data reports weekly until four weeks after the first study injections are administered to all subjects, and then monthly until 4 weeks after all subjects have completed the second study injections. The PSRT will be notified and convened to review any study pauses. The Protocol Chair, IND Sponsor Medical Officer, the UNI-CPSC Medical Monitor will continue to monitor the cumulative study safety data reports on at least a monthly basis through completion of the last study visit.

9 ADMINISTRATION AND LEGAL OBLIGATIONS

9.1 PROTOCOL INITIATION, AMENDMENTS AND TERMINATION

Each site must receive IRB approval and approval of The EMMES Corporation before initiating the study at the site. All amendments will also be submitted to the site IRBs for approval. The VRC, NIAID, NIH reserves the right to terminate the study. Each IoR will notify the respective site IRB of the study termination in writing and provide documentation to The EMMES Corporation.

9.2 STUDY DOCUMENTATION AND STUDY RECORDS RETENTION

The site IoR will maintain a list of appropriately qualified persons to whom trial duties have been delegated. The site IoR is responsible for ensuring that staff maintains a comprehensive and centralized filing system of all study-related (essential) documentation, suitable for inspection at any time by representatives from the VRC, IRB, The EMMES Corporation and/or applicable regulatory authorities. Elements include but are not limited to:

- Subject files containing completed informed consent forms and supporting copies of source documentation
- Study files containing the protocol with all amendments and copies of all correspondence with the IRB

In addition, all original source documentation must be maintained and readily available.

The EMMES Corporation is responsible for ensuring that records and documents pertaining to the conduct of this study, including CRFs, source documents, consent forms, laboratory test results, and medication inventory records, are retained by the investigator for at least 2 years following approval of a Biologics License Application or until VRC, NIAID authorizes transfer or destruction of study records. No study records will be destroyed without prior authorization from NIAID.

9.3 DATA COLLECTION AND PROTOCOL MONITORING

9.3.1 Data Capture Methods

Clinical research data will be collected and recorded by the study sites in a timely fashion in a secure electronic web-based clinical data management system (CDMS) provided by The EMMES Corporation as defined by the contract. Immunological testing on collected, coded blood samples may be performed in batches at central laboratories. Extracted data without subject identifiers will be sent to the statisticians for statistical analysis as needed. The final

study database and statistical evaluations will be transferred to the VRC, NIAID at the study completion.

9.3.2 Source Documents and Access to Source Data/Documents

Each participating site will maintain appropriate medical and research records for this trial, in compliance with ICH-GCP, regulatory and institutional requirements for the protection of confidentiality of subjects. As part of participating in the NIAID-sponsored study, each site will permit authorized representatives of the VRC, NIAID, and regulatory agencies to examine (and when required by applicable law, to copy) clinical records for the purposes of quality assurance reviews, audits and evaluation of the study safety and progress.

Source data are all information, original records of clinical findings, observations, or other activities in a clinical trial necessary for the reconstruction and evaluation of the trial. Examples of these original documents and data records include, but are not limited to, medical records, laboratory reports, pharmacy records and other research records maintained for the clinical trial.

9.3.3 Protocol Monitoring

The study data integrity and compliance with the protocol will be assured by the monitoring of the study documentation and study conduct at the sites by The EMMES Corporation. Routine data monitoring and protocol compliance will be performed by the site investigators and study coordinator on an ongoing basis. The study clinical monitoring plan and the data quality monitoring plan will be developed and followed by The EMMES Corporation in consultation with the VRC Program Officer.

9.4 LANGUAGE

All written information and other material to be used by subjects and investigative staff must use vocabulary and language that are readily understood.

9.5 POLICY REGARDING RESEARCH-RELATED INJURIES

The study site will provide immediate medical care for any injury resulting from participation in this research. In general, the VRC, the NIH, or the Federal Government will not provide long-term medical care or financial compensation for research-related injuries.

10 REFERENCES

1. CDC, *Serum cross-reactive antibody response to a novel influenza A (H1N1) virus after vaccination with seasonal influenza vaccine.* MMWR - Morbidity & Mortality Weekly Report, 2009. **58**(19): p. 521-4.
2. Wei, C.-J., et al., *Cross-Neutralization of 1918 and 2009 Influenza Viruses: Role of Glycans in Viral Evolution and Vaccine Design.* Science Translational Medicine, 2010. **2**(24): p. 24ra21.
3. Wei, C.J., et al., *Induction of broadly neutralizing H1N1 influenza antibodies by vaccination.* Science, 2010. **329**(5995): p. 1060-4.
4. Ledgerwood, J.E., et al., *DNA priming and influenza vaccine immunogenicity: two phase 1 open label randomised clinical trials.* Lancet Infect Dis, 2011. **11**(12): p. 916-924.
5. Subbarao, K., B.R. Murphy, and A.S. Fauci, *Development of effective vaccines against pandemic influenza.* Immunity, 2006. **24**(1): p. 5-9.
6. Luke, C. and K. Subbarao, *Vaccines for pandemic influenza.* Emerging Infectious Diseases, 2006. **12**(1): p. 66-72.
7. WHO, *Influenza (Seasonal) Fact Sheet*. 2009: Geneva, Switzerland.
8. Kilbourne, E., *Influenza pandemics of the 20th century.* Emerg Infect Dis [serial on the Internet]. 2006. **available from http://www.cdc.gov/ncidod/EID/vol12no01/05-1254.htm**.
9. CDC, *Prevention and Control of Influenza with Vaccines: Interim Recommendations of the Advisory Committee on Immunization Practices (ACIP), 2013.* MMWR Morb Mortal Wkly Rep, 2013. **62**(18): p. 356.
10. Fiore, A.E., et al., *Prevention and control of seasonal influenza with vaccines: recommendations of the Advisory Committee on Immunization Practices (ACIP), 2009.* MMWR Recomm Rep, 2009. **58**(RR-8): p. 1-52.
11. Targonski, P.V., R.M. Jacobson, and G.A. Poland, *Immunosenescence: role and measurement in influenza vaccine response among the elderly.* Vaccine, 2007. **25**(16): p. 3066-9.
12. Harper, S.A., et al., *Prevention and control of influenza: recommendations of the Advisory Committee on Immunization Practices (ACIP).* MMWR Recomm Rep, 2004. **53**(RR-6): p. 1-40.
13. Novel Swine-Origin Influenza, A.V.I.T., *Emergence of a Novel Swine-Origin Influenza A (H1N1) Virus in Humans.* N Engl J Med, 2009. **360**(25): p. 2605-2615.
14. Alexander, J., et al., *Identification of broad binding class I HLA supertype epitopes to provide universal coverage of influenza A virus.* Human Immunology, 2010. **71**(5): p. 468-474.
15. Wang, T.T., et al., *Broadly Protective Monoclonal Antibodies against H3 Influenza Viruses following Sequential Immunization with Different Hemagglutinins.* PLoS Pathog, 2010. **6**(2): p. e1000796.
16. Thomas, P.G., et al., *Cell-mediated protection in influenza infection.* Emerging Infectious Diseases, 2006. **12**(1): p. 48-54.
17. Doherty, P.C., *Toward a broadly protective influenza vaccine.* The Journal of Clinical Investigation, 2008. **118**(10): p. 3273-3275.
18. Jameson, J., J. Cruz, and F.A. Ennis, *Human Cytotoxic T-Lymphocyte Repertoire to Influenza A Viruses.* The Journal of Virology, 1998. **72**(11): p. 8682-8689.

19. McElhaney, J.E. and J.P. Dutz, *Better influenza vaccines for older people: what will it take?* J Infect Dis, 2008. **198**(5): p. 632-4.
20. McElhaney, J.E., et al., *T Cell Responses Are Better Correlates of Vaccine Protection in the Elderly.* J Immunol, 2006. **176**(10): p. 6333-6339.
21. Frenck, R.W., Jr., et al., *Comparison of the immunogenicity and safety of a split-virion, inactivated, trivalent influenza vaccine (Fluzone(R)) administered by intradermal and intramuscular route in healthy adults.* Vaccine, 2011. **29**(34): p. 5666-74.
22. Belshe, R.B., et al., *Serum antibody responses after intradermal vaccination against influenza.* N Engl J Med, 2004. **351**(22): p. 2286-94.
23. Ansaldi, F., et al., *Fluzone((R)) Intradermal vaccine: a promising new chance to increase the acceptability of influenza vaccination in adults.* Expert review of vaccines, 2012. **11**(1): p. 17-25.
24. Tavel, J.A., et al., *Safety and immunogenicity of a Gag-Pol candidate HIV-1 DNA vaccine administered by a needle-free device in HIV-1-seronegative subjects.* J Acquir Immune Defic Syndr, 2007. **44**(5): p. 601-5.
25. Graham, B.S., et al., *Phase 1 safety and immunogenicity evaluation of a multiclade HIV-1 DNA candidate vaccine.* J Infect Dis, 2006. **194**(12): p. 1650-1660.
26. Catanzaro, A.T., et al., *Phase I clinical evaluation of a six-plasmid multiclade HIV-1 DNA candidate vaccine.* Vaccine, 2007. **25**(20): p. 4085-92.
27. Koup, R.A., et al., *Priming Immunization with DNA Augments Immunogenicity of Recombinant Adenoviral Vectors for Both HIV-1 Specific Antibody and T-Cell Responses.* PLoS One, 2010. **5**(2): p. e9015.
28. Kibuuka, H., et al., *A phase 1/2 study of a multiclade HIV-1 DNA plasmid prime and recombinant adenovirus serotype 5 boost vaccine in HIV-Uninfected East Africans (RV 172).* J Infect Dis, 2010. **201**(4): p. 600-7.
29. Jaoko, W., et al., *Safety and immunogenicity study of Multiclade HIV-1 adenoviral vector vaccine alone or as boost following a multiclade HIV-1 DNA vaccine in Africa.* PLoS One, 2010. **5**(9): p. e12873.
30. Churchyard, G.J., et al., *A Phase IIA Randomized Clinical Trial of a Multiclade HIV-1 DNA Prime Followed by a Multiclade rAd5 HIV-1 Vaccine Boost in Healthy Adults (HVTN204).* PLoS One, 2011. **6**(8): p. e21225.
31. Koblin, B.A., et al., *Safety and immunogenicity of an HIV adenoviral vector boost after DNA plasmid vaccine prime by route of administration: a randomized clinical trial.* PLoS One, 2011. **6**(9): p. e24517.
32. Martin, J.E., et al., *A DNA vaccine for Ebola virus is safe and immunogenic in a phase I clinical trial.* Clin Vaccine Immunol, 2006. **13**(11): p. 1267-77.
33. Martin, J.E., et al., *A SARS DNA vaccine induces neutralizing antibody and cellular immune responses in healthy adults in a Phase I clinical trial.* Vaccine, 2008. **26**(50): p. 6338-43.
34. Martin, J.E., et al., *A West Nile virus DNA vaccine induces neutralizing antibody in healthy adults during a phase 1 clinical trial.* J Infect Dis, 2007. **196**(12): p. 1732-40.
35. Ledgerwood, J.E., et al., *A West Nile virus DNA vaccine utilizing a modified promoter induces neutralizing antibody in younger and older healthy adults in a phase I clinical trial.* J Infect Dis, 2011. **203**(10): p. 1396-404.

36. Graham, B.S., et al. *Evaluation of candidate DNA HIV-1 vaccine delivery by Biojector or needle and syringe in healthy adults (VRC 008)*. in *AIDS Vaccine 2007*. 2007. Seattle, WA.
37. Betts, M.R., J.P. Casazza, and R.A. Koup, *Monitoring HIV-specific CD8+ T cell responses by intracellular cytokine production.* Immunol Lett, 2001. **79**(1-2): p. 117-25.
38. Helms, T., et al., *Direct visualization of cytokine-producing recall antigen-specific CD4 memory T cells in healthy individuals and HIV patients.* J Immunol, 2000. **164**(7): p. 3723-32.
39. Barouch, D.H., et al., *A human T-cell leukemia virus type 1 regulatory element enhances the immunogenicity of human immunodeficiency virus type 1 DNA vaccines in mice and nonhuman primates.* J Virol, 2005. **79**(14): p. 8828-34.
40. Yang, Z.Y., et al., *A DNA vaccine induces SARS coronavirus neutralization and protective immunity in mice.* Nature, 2004. **428**(6982): p. 561-4.

Appendix I: Template Informed Consent Form

The sample informed consent forms are provided to guide development of a site-specific consent form. Only an IRB-approved consent forms will be used to consent subjects for participation in the study.

Study Consent, Template Version 4.0

Template Informed Consent Form for Study Participation

STUDY TITLE: VRC 703: An Open-Label, Randomized Phase 1b Study of the Safety and Immunogenicity of Investigational Seasonal Influenza DNA Vaccine (HA DNA) Followed by Trivalent Inactivated Vaccine (TIV) Administered Intradermally (ID) or Intramuscularly (IM) in Healthy Adults Ages 18-70 Years

INTRODUCTION

We invite you to take part in a research study at the ___________________________.

The study is sponsored by the National Institutes of Health (NIH). It is up to you to decide if you want to be a part of this study. This is no penalty or loss of benefits for choosing to not participate. Please ask questions and discuss this study with anyone you want. Take as much time as you need to decide.

[SCREENING (delete this section if site has separate screening protocol or separate screening consent)]

Before you can enroll in this vaccine research study, you will be screened for eligibility. You will need to sign this consent form before we can do the screening.

Screening involves a physical exam and blood tests to check your general health status. If you are a woman, you will be asked about your health related to the possibility of becoming pregnant, birth control use and tested for pregnancy if applicable. During screening, we may collect some blood to store for research. We will ask you about your general health history and influenza history. We will ask you about medications you are taking and recent vaccinations.

We will review the screening results with you and tell you if the screening results may show you are not eligible to join the study. If you enroll in this study, you cannot be in another research study at the same time where you receive a study product or have blood drawn for research.

PURPOSE OF THE VACCINE STUDY

This research is about vaccines for seasonal influenza (“flu”). Three different types of flu vaccines are being studied. Two are regular seasonal flu vaccines that are approved by the Food and Drug Administration (FDA) for use in the influenza season. The other vaccine is experimental. The experimental vaccine is not approved by the FDA for preventing “flu” infection. Six different vaccination schedules, involving one or two of the vaccine types are being studied.

One main purpose of this study is to see if the experimental vaccine is safe and if there are any side effects. The other main purpose is to see which vaccination schedules result in the best immune responses. The main “immune response” of interest is “antibodies” to influenza. This is studied using blood samples in research laboratories.

You are eligible to participate in this study because:

- you have completed the screening process,
- you are at least 18 years old and meet the upper age limit that applies at the time of your enrollment (70 years prior to 11/16/2012 or will not be older than 64 years at the time of next year's vaccination if enrolled after 11/16/2012).
- you have physical exam and blood test results that meet eligibility requirements, and
- you do not have any significant medical problems as determined during your screening.

The study plan is to enroll 330 people in this study at several sites through the U.S. While in the study, you will be monitored for vaccine-related side effects. In this study, the study injections (shots) will be given to people in the upper arm(s).

STUDY INJECTIONS

Vaccines are substances used to try to create resistance (or immunity) to a disease. You cannot get flu infection from study injections because none of the injections contain influenza virus.

TIV: Both of the licensed vaccines for the prevention of seasonal flu in this study are "trivalent inactivated vaccine." It is referred to in this consent as "TIV." One of them is made to be given in the skin (intradermal or "ID"). The other one is made to be given in muscle (intramuscular or "IM"). TIV is made from influenza virus grown in hens' eggs, which is then inactivated, purified and prepared in a sterile preparation in a salt water solution. Every year a decision is made by the FDA about which influenza strains are likely to cause illness and the new season's TIV is based on these 3 strains of influenza. TIV has proteins from the three strains of the flu virus in it. The TIV used in this study is manufactured by Sanofi Pasteur under the brand name "Fluzone®". The TIV vaccines used in this study have been purchased from a commercial supplier; Sanofi Pasteur is not involved in the design or conduct of the study.

DNA vaccine: The experimental vaccine is a "DNA" vaccine. DNA is used by the body as instructions (a code) for making proteins. The DNA vaccine is a sterile preparation in a salt water solution. After the DNA vaccine is injected, this code is used by your body to make proteins based on three strains of flu included in the vaccine. DNA vaccines made by NIH have been tested against a variety of infections. Between 1 and 4 injections of a DNA vaccine have been given to more than 2300 adults over the past 10 years.

STUDY PROCEDURES

Six groups of adults will be enrolled in the study. Enrollments into Group 1 and 2 will occur first. Then, enrollments into Groups 3, 4, 5 and 6 will start. If you agree to take part in the study, you will be randomly assigned (by chance, like flipping a coin) to a study group. You will not know what group you will get before you enroll, but will find out right after enrolling into the study. You will know what vaccine type is in each injection before it is given.

All injections in the study will be given in the upper arm muscle. Each group has a schedule of study visits that includes two injection days. The first injection day is called "Day 0". The second injection day varies by schedule and is referred to by "Study Week" in this consent.

Group 1 will get DNA vaccine on Day 0 and TIV ID at Study Week 14.

Group 2 will get DNA vaccine on Day 0 and TIV IM at Study Week 14.

The protocol was amended between the enrollment of Groups 1 and 2 and the initiation of Groups 3 to 6. Because this involved a change in TIV products to be used and because TIV ID is not approved for adults older than 64, any Group 1 subject who will be 65 or older at the time of their second scheduled vaccination will be given TIV IM rather than TIV ID.

Group 3 will get TIV ID on Day 0 and TIV ID at Study Week 44.

Group 4 will get TIV IM on Day 0 and TIV IM at Study Week 44.

Group 5 will get both a DNA vaccine in the left arm AND TIV ID in the right arm on Day 0, but only TIV ID at Study Week 44.

Group 6 will get both a DNA vaccine in the left arm AND TIV IM in the right arm on Day 0, but only TIV IM at Study Week 44.

Both vaccinations given to Group 1 and Group 2 are based on flu strains included in the flu season called “2012/13”. For Groups 3, 4, 5 and 6 the Day 0 injections are based on 2012/13 flu season, while the Study Week 44 injections will be based on the next flu season called “2013/14.”

DNA vaccine injections will be given using a needleless system called the Biojector 2000®. This device delivers the vaccine through the skin without the use of a needle. It uses the pressure of carbon dioxide (CO_2) instead of a needle to inject the vaccine through your skin and into the muscle. This system has FDA clearance for delivering vaccine injections into muscles. The TIV vaccine injections will be given as usual with a needle and syringe.

The study schedules from screening through completion include 6 planned clinic visits and a telephone contact after each vaccination. Clinic visits occur over 38 weeks for Group 1 and Group 2 and over 68 weeks for Groups 3, 4, 5 and 6. Research studies require following a set schedule for injections and follow-up visits in order to answer the study research questions. Some flexibility in scheduling is permitted, but it is important that you work with the staff to stay on schedule. The clinic staff will observe you for 15-30 minutes after each vaccination. One to two days after each injection, you will talk to a clinic staff member to report on how you are doing. You will also complete a diary card for 7 days at home. This will require that you record your temperature and symptoms and look at the injection site on your arm each day. The clinic staff is available to you by phone 24 hours a day to report any unexpected side effects.

You may record your symptoms on a paper diary card or enter them into a secure electronic form using the internet. If you choose to report your symptoms through the internet you will be trained by the clinic staff and given a username and password. If you have any symptoms that interfere with your usual activities, it may be necessary to come to the study clinic for an examination before your next scheduled visit. It is very important that you follow the instructions given to you by the clinic staff.

At each visit, you will be checked for any changes in your health or problems since your last visit. You will be asked how you are feeling and if you have taken any medications. Blood will

be drawn at scheduled study visits to check on your health and to study your immune response to the vaccine. You will be told promptly if any of your test results show a health problem. Results of immune response tests are not tests used to check on your health and will not be given to you during the study. The study staff will show you the visit schedule plan before you enroll in the study.

The amount of blood drawn each visit will vary from about 4 tablespoons (60 mL) to about 7 tablespoons (105 mL), depending on the visit. You might also be asked to have laboratory tests between regular visits if needed to evaluate a change in your health.

MONITORING OF THE STUDY

This study will be monitored by a group of physicians and scientists associated with the National Institutes of Health. This group will review the information from the study and will pay close attention to harmful reactions.

GENETIC TESTING

Some of the blood drawn from you as part of this study will be used for genetic tests. There are many different types of genetic tests. In vaccine research, some genetic tests are done to see if different types of immune responses to a vaccine seem to be related to genetic differences in people. These tests will be done in a research lab using your stored samples. Genetic tests done in a research lab will **not** be in your medical record. Tests that are done in a research lab will **not** have your name on the sample given to the research lab. In the future, genetic research tests to help understand how vaccines work may be done on your DNA using stored samples.

STORED SAMPLES

You may not participate in this study if you are not willing to have your blood samples stored for future research purposes.

During your participation on this study blood samples will be collected from you. We will store these samples for future research to learn more about influenza virus, vaccines, the immune system, and/or other medical conditions.

The results from the research done with your stored samples will not be given to your health care provider and will not be put in your medical record. This is because the research test results, unlike routine medical testing, will not be for evaluating your health.

Labeling of Stored Samples

Your stored samples will be labeled by a code (such as a number) that only the study team can link to you. Any identifying information about you will be kept confidential to the extent permitted by law. Despite protections, there is a small chance that information identifying you will be given to someone who should not get it.

Future Studies

In the future, other investigators at NIH or outside of NIH may wish to study your stored samples. When the study team shares your stored samples, they will be marked with a code, but will not have any identifying information on them. Some information about you, such as your

gender, age, health history, or ethnicity may also be shared with other investigators. Any future research studies completed using your samples will be reviewed by the investigator's Institutional Review Board (IRB), a special committee that oversees medical research studies to protect the rights and welfare of human subjects.

Your stored materials will be used only for research and will not be sold. The research done with your materials may be used to develop new products in the future but you will not receive payment for such products.

POSSIBLE STUDY RISKS

***Possible* risks from the injections:** temporary stinging, pain, redness, soreness, itchiness, swelling, bruising, or a cut in the arm. There is a very small chance of infection.

***Possible* risks of blood drawing:** pain, bleeding, bruising, feeling lightheaded, or fainting.

***Possible* risks from genetic testing:** unintended release of information that could be used by insurers or employers; discovering a gene or HLA type that suggests risk of disease for you or your family; discovering undisclosed family relationships.

***Possible* risks from any vaccine:** fever, chills, rash, aches and pains, nausea, headache, dizziness, and fatigue. Some people have allergic reactions to vaccines. These types of reactions are usually greatest within the first 24 hours after vaccination and typically last 1 to 3 days. Over-the-counter medicine, such as acetaminophen, will generally help relieve symptoms from vaccination and may be used.

***Possible* risks from DNA vaccine:** temporary drop in white blood cell count, sore arm, skin rash or hives, some people get a small red bump and then a scab for a few days where the shot is given.

***Possible* risks from the seasonal influenza TIV IM and TIV ID:** fever, muscle and general body aches, headache, fatigue, hoarseness, sore, red or itchy eyes, cough, itching, and nausea. A severe allergic reaction can occur especially in people that are allergic to eggs. In 1976, a small number of people who got an inactivated swine flu vaccine developed a severe nerve weakness called Guillain-Barré syndrome. Local reactions of redness, swelling and itchiness occur more frequently after ID than after IM injection of TIV. The "Vaccine Information Statement" that will be provided for 2012-2013 and 2013-2014 influenza vaccinations have more information.

***Unknown* safety risks:** There may be side effects from the study vaccines - even serious or life threatening ones- that we do not yet know about. Please tell the study staff about any side effect you think you are having. This is important for your safety.

***Possible* risks from Pregnancy:** TIV is generally recommended for use in pregnant women in the U.S. We do not know the effects of the experimental DNA vaccine on a fetus or nursing infant. In this study, women who can have children must not breast-feed and must use effective birth control starting at least 21 days before getting the first study injection until 3 weeks after the last study injection. Effective birth control includes not having sex, or using condoms, a diaphragm or cervical cap with a spermicidal gel or foam, an intrauterine device, a male partner who had a vasectomy or birth control pills, patches or other prescription methods. We

will ask about the outcome of any pregnancy that begins during study participation.

***Other* Risks:** It is unknown if the study vaccine may alter your response to any future infections you may have with influenza viruses.

You will be made aware of significant health effects of the vaccine and serious side effects if they would occur in other subjects, and will be updated during the trial as needed.

You may not donate blood at a blood bank while participating in this research study or for one year after the date of the last experimental vaccine injection. If you receive only TIV while in the study you may donate blood immediately after completion of the study.

POSSIBLE BENEFITS

This study is not designed to benefit you. Receiving the seasonal influenza TIV ID or TIV IM may protect you against influenza, but these vaccines do not always work to prevent influenza. The DNA vaccine alone is not expected to provide protection from influenza. Receiving the DNA vaccine may change the immune response to the TIV vaccine given. You and others may benefit in the future from the information that will be learned from the study.

COSTS OF PARTICIPATION

There are no costs to you for participating in this study. All medical costs for the care you receive outside this study will be paid by you or your health insurance carrier (if you have insurance). It is possible that you may have some expenses that are not covered by the study compensation provided.

COMPENSATION TO YOU FOR YOUR PARTICIPATION

You will be compensated [insert site IRB-approved amount] for each visit that does not include an injection but does include a blood draw and [insert] for each injection visit. For visits that do not include an injection or a blood draw, you will be compensated [insert]. The approximate total compensation is from [insert]. This will be based on the number of study visits you attend and study injections you receive. You will be paid throughout the study after each visit.

REASONS FOR REMOVING YOU FROM THE STUDY WITHOUT YOUR CONSENT

The study doctor can take you out of this study without your permission *if:*

- continuing in the study could harm you,
- you do not follow study instructions or keep appointments, or
- the study is stopped by the NIH, regulatory boards or the FDA.

If you agree to take part in this study, it is important for you to keep all your appointments. However, if you don't want to stay in the study, you can leave at any time. You will not lose any benefits that you would have had if you had not joined the study.

If you receive the first study injection but not the following injections for any reason, you will be asked to continue with follow-up visits until the end of the study. It is important to continue to monitor your health even if you do not receive all study injections.

ALTERNATIVES

This study is not designed to treat any disease. You may choose to not participate.

CONFIDENTIALITY

A description of this clinical trial will be available on *http://www.ClinicalTrials.gov*, as required by U.S. Law. This Web site will not include information that can identify you. At most, the Web site will include a summary of the results. You can search this Web site at any time.

Results of NIH-supported research study may also be reported in medical journals, on internet or at scientific meetings. These reports will not include information that can identify you.

In most cases, the NIH will not release any information about your research involvement without your written permission. However, if you sign a release of information form, for example, for an insurance company, the insurance company receives information from your medical record. This information might affect (either favorably or unfavorably) the willingness of the insurance company to sell you insurance.

The Federal Privacy Act protects the confidentiality of your medical records. However, you should know that the Act allows release of some information from your medical record without your permission, for example, if it is required by regulatory agencies that oversee the study, law enforcement officials, or other authorized people.

POLICY REGARDING RESEARCH-RELATED INJURIES

The study site will provide immediate medical care for any injury resulting from your participation in research here. In general, no long-term medical care or financial compensation for research-related injuries will be provided by the National Institutes of Health or the Federal Government. However, you have the right to pursue legal remedy if you believe that your injury justifies such action.

Problems or Questions. If you have any problems or questions about this study, or about your rights as a research participant, or about any research-related injury, contact the investigator of record,_______________. Others you may call are the Study Coordinator _____________at________. You may also call the __________ Patient Representative at ______________.

Please keep a copy of this document in case you want to read it again.

Adult Participant Consent:

I have read the explanation about this study and have been given the opportunity to discuss it and ask questions. I consent to take part in this study.

[**Signature Section Consistent with Site IRB Template]**

Appendix II: Contact Information

VRC Scientific/Medical Researchers:

Protocol Chair
Julie Ledgerwood, D.O.
301-594-8502
Deputy Chief, Clinical Trials Core
Vaccine Research Center, NIAID, NIH
Bethesda, MD 20892

Protocol Co-Chair
Uzma Sarwar, M.D.
301-402-9043
Vaccine Research Center, NIAID, NIH
Bethesda, MD 20892

IND Sponsor Medical Officers:
Barney Graham, M.D., Ph.D.
301-594-8468
Chief, Clinical Trials Core
Vaccine Research Center, NIAID, NIH
Bethesda, MD 20892-3017

Joseph Casazza, M.D., Ph.D.
301-594-8627
Vaccine Research Center, NIAID, NIH
Bethesda, MD 20892

Contracting Officers' Technical Representative (COTR):
Brenda Larkin, RN, BSN, CCRC
301-594-8542
Vaccine Research Center, NIAID, NIH
Bethesda, MD 20892

Protocol Statistician:
Abbie Bellamy, Ph.D.
Kelly Schlessman, M.S.
The EMMES Corporation; 301-251-1161

VRC Protocol Operations Manager:
Mary E. Enama, M.A., PA-C, 301-594-8501
Vaccine Research Center, NIAID, NIH
Bethesda, MD 20892

VRC Protocol Specialist:
Galina Yamshchikov, M.S., 301-594-1064
Vaccine Research Center, NIAID, NIH
Bethesda, MD 20892

CRO Authorized to Conduct the Study on Behalf of the VRC/NIAID/NIH:
The EMMES Corporation
401 N. Washington Street, Suite 700
Rockville, MD 20850

Scientific and Laboratory Collaborators:
Vaccine Research Center, NIAID, NIH
40 Convent Drive, Bethesda, MD 20892
Robert Bailer, Ph.D., 301-594-8481
Richard Koup, M.D., 301-594-8585
John Mascola, M.D., 301-594-8490
Mario Roederer, Ph.D., 301-594-8491
Daniel Douek, M.D., Ph.D., 301-594-8484
Robert Seder, M.D., 301-594-8483

Research Immunology Central Laboratory:
NVITAL (NIAID Vaccine Immune T-Cell and Antibody Laboratory)
9 West Watkins Mill Road, Suite 150
Gaithersburg, MD 20878

VRC Vaccine Production Program
Richard Schwartz, Ph.D. 301-594-8485
Vaccine Research Center, NIAID, NIH
Bethesda, MD 20892

Other Immunology Testing Labs:
Bioqual, Inc.
9600 Medical Center Dr., Rockville, MD 20850

UNI-CPSC Operations Management Center
Thad Zajdowicz, M.D., MPH, Project Director and Medical Monitor
Alternate Med. Monitor: Robert Lindblad, M.D.
Phyllis Renehan, Project Manager
The EMMES Corporation, 301-251-1161
401 N. Washington Street, Suite 700
Rockville, MD 20850

IND Sponsor Regulatory Affairs
Michelle Conan-Cibotti, Ph.D. 301-451-2740
Florence Kaltovich, MS, MHS; 301-402-2402
Vaccine Research Center, NIAID, NIH
Bethesda, MD 20892

IND Sponsor Authorized Representative
Jamie Winestone 301-251-1161 ext. 230
UNI-CPSC Regulatory Director
The EMMES Corporation

Serious Adverse Event Reporting:
Email: uniflu@emmes.com
Fax: 301-576-3558

VRC 703 Study Sites	Site Investigators of Record
Baylor College of Medicine<br>Houston, TX	Shital Patel, MD
University of Iowa<br>Iowa City, IA	Patricia Winokur, MD
Saint Louis University<br>St. Louis, MO	Robert Belshe, MD
Stanford University Medical Center<br>Stanford, CA	Cornelia Dekker, MD

Appendix III: Schedule of Evaluations

SCHEDULE A									
		**Screen**	**VRC 703 Schedule of Evaluations for Group 1 and Group 2**						
**Visit**		01	02	02A	03	05	05A	06	07
**Week of Study**		-10 to 0	W 0	W1	W 3	W 14	W 15	W17	W 38
[1]**Day of Study**		-70 to 0	D 0	D 7	D 21	D 98	D 105	D 119	D 266
**Clinical Evaluations**	**Tube**								
[1]Informed Consent		X	X						
Physical exam for eligibility at screen; BP, pulse, temp, wt other visits; targeted exam.		X	X		X	X		X	X
Medical history targeted to eligibility at screen; interim history for AEs other visits		X	X		X	X		X	X
[2] Study Vaccinations			X			X			
Begin 7-Day Diary Card			X			X			
Telephone contact; clinic visit if indicated				X			X		
Counseling on pregnancy prevention			X		X				
CBC, platelets	EDTA	3	3		3				
[3] Pregnancy test: urine (or serum)		X	X		X	X			
Creatinine and ALT	SST	4							
**Research Immunology**									
Antibody assays and serum storage	SST	16[4]	24		32	32		32	32
PBMC and plasma for storage	EDTA	40[4]	80		60	60		60	60
**Daily Volume (mL)**		**63**	**107**	**-**	**95**	**92**	**-**	**92**	**92**
**Max. Cumulative Volume (mL)**		**63**	**170**	**-**	**265**	**357**	**-**	**449**	**541**

[1] Screening informed consent may be signed more than 8 weeks prior to study enrollment; screening evaluations may be repeated, if needed.
The screening blood draw for research does not need to be repeated if it was collected more than 70 days prior to enrollment. Day 0=day of enrollment and HA DNA vaccine injection. Day 0 evaluations prior to first injection are the baseline for assessing adverse events subsequently.

[2] Complete post vaccination evaluations (BP, pulse and injection site assessment) at 15-60 minutes after study injections.

[3] Negative pregnancy test results must be confirmed for women of reproductive potential prior to administering each injection. TIV is not contraindicated for pregnant women, but the woman must be informed of result prior to administering TIV as part of this research protocol. If pregnant, research blood draws will be one-fourth of shown volume or eliminated if the pregnant participant is anemic.

Visit windows: Schedule Visits 02A through 05 with respect to Day 0; the following visit windows apply: 02A(+4 days); Visits 03(+7 days); Visit 05 (±14 days). Schedule Visits 05A through 07 with respect to actual Visit 05 date; the following windows apply: 05A(+4 days); Visits 06 (+7 days), visit 07 (±7 day window). The schedule for Groups 1 and 2 does not include a “visit 04”.

[4]Storage samples of peripheral blood mononuclear cells (PBMCs) and serum collected during screening may be used for assay validation and site proficiency testing.

SCHEDULE B										
		**Screen**	**VRC 703 Schedule of Evaluations, Groups 3-6**							
**Visit**		01	02	02A	03	05	05A	06	07
**Week of Study**		-10 to 0	W 0	W1	W 3	W 44	W 45	W47	W 68
**[1]Day of Study**		-70 to 0	D 0	D7	D 21	D 308	D 315	D 329	D 476
**Clinical Evaluations**	**Tube**								
[1]Informed Consent		X	X						
Physical exam for eligibility at screen; BP, pulse, temp, wt other visits; targeted exam.		X	X		X	X		X	X
Medical history targeted to eligibility at screen; interim history for AEs other visits		X	X		X	X		X	X
[2] Study Vaccinations			X			X			
Begin 7-Day Diary Card			X			X			
Telephone contact; clinic visit if indicated				X			X		
Counseling on pregnancy prevention			X		X				
CBC, platelets	EDTA	3	3		3				
[3] Pregnancy test: urine (or serum)		X	X		X	X			
Creatinine and ALT	SST	4							
**Research Immunology**									
Antibody assays and serum storage	SST	16[4]	24		32	32		32	32
PBMC and plasma for storage	EDTA	40[4]	80		60	60		60	60
**Daily Volume (mL)**		**63**	**107**	**-**	**95**	**92**	**-**	**92**	**92**
**Max. Cumulative Volume (mL)**		**63**	**170**	**-**	**265**	**357**	**-**	**449**	**541**

[1] Screening informed consent may be signed more than 8 weeks prior to study enrollment; screening evaluations may be repeated, if needed.
The screening blood draw for research does not need to be repeated if it was collected more than 70 days prior to enrollment. Day 0=day of enrollment and HA DNA vaccine injection. Day 0 evaluations prior to first injection are the baseline for assessing adverse events subsequently.
[2] Complete post vaccination evaluations (BP, pulse and injection site assessment) at 15-60 minutes after study injections.
[3] Negative pregnancy test results must be confirmed for women of reproductive potential prior to administering each injection. TIV is not contraindicated for pregnant women, but the woman must be informed of result prior to administering TIV as part of this research protocol. If pregnant, research blood draws will be one-fourth of shown volume or eliminated if the pregnant participant is anemic.
Visit windows: Schedule Visits 02A through 03 with respect to Day 0; the following visit windows apply: Visit 02A (+4 days); Visit 03(+7 days).
Schedule Visit 05 so that the 2013/2014 TIV injection is administered before the end of October 2013; for convenience this is shown as "Week 44" in the schedule.
Schedule Visits 05A through 07 with respect to actual Visit 05 date; the following windows apply:
Visit 05A(+4 days); Visit 06 (+7 days), Visit 07 (±7 day window).
[Note: "Visit 04" was deleted from the schedule for Groups 3-6 before any subjects reached this visit.]
[4]Storage samples of peripheral blood mononuclear cells (PBMCs) and serum collected during screening may be used for assay validation and site proficiency testing.

Appendix IV: Assessment of Relationship to Vaccine and Adverse Event Severity Grading

<u>Assessment of Causality Relationship of an Adverse Event (AE) to Study Vaccine</u>:

The relationship between an AE and the vaccine will be assessed by the investigator on the basis of his or her clinical judgment and the definitions below.

- **Definitely Related**. The AE and administration of study agent are related in time, and a direct association can be demonstrated.
- **Probably Related**. The AE and administration of study agent are reasonably related in time, and the AE is more likely explained by study agent than other causes.
- **Possibly Related**. The AE and administration of study agent are reasonably related in time, but the AE can be explained equally well by causes other than study agent.
- **Not Related**. There is not a reasonable possibility that the AE is related to the study agent.

For purposes of preparing data reports in which AE attributions are limited to "**Related**" or "**Not Related**", in this protocol, the "Definitely, Probably and Possibly" attributions will be mapped to the "Related" category. The definitions that apply when these two categories alone are used are as follows:

- **Related** – There is a reasonable possibility that the AE may be related to the study agent.
- **Not Related** – There is not a reasonable possibility that the AE is related to the study agent.

Grading the Severity of Adverse Events:
The FDA Guidance for Industry (September 2007): "Toxicity Grading Scale for Healthy Adult and Adolescent Volunteers Enrolled in Preventive Vaccine Clinical Trials" is the basis for the severity grading of adverse events in this protocol. Several modifications were made to the table as follows:

- "Emergency room visit" is not automatically considered a life-threatening event; these words have been removed from any "grade 4' definition where they appear in the table copied from the guidance document.
- Any laboratory value shown as a "graded" value in the table that is within the institutional normal range will not be severity graded or recorded as an adverse event.
- Severity grading for hemoglobin decrease on the basis of the magnitude of decrease from baseline is not applicable at the grade 1 level; only absolute hemoglobin will be used to define grade 1 decrease. Increases in hemoglobin are AEs only for values above the upper limit of normal and are graded by the systemic illness clinical criteria.
- Severity grading definition for Grade 4 local reaction to injectable product (Erythema/Redness and Induration/Swelling) included added text "requiring medical attention".
- 1 X ULN was removed from the definition for PT increase.
- Severity grading definition for hypotension includes added clarifications such that an asymptomatic low blood pressure reading is not an adverse event.

When not otherwise specified in the table, the following guidance will be used to assign a severity grade:

Grade 1 (Mild): No effect on activities of daily living
Grade 2 (Moderate): Some interference with activity not requiring medical intervention
Grade 3 (Severe): Prevents daily activity and requires medical intervention
Grade 4 (Life-threatening): Hospitalization; immediate medical intervention or therapy required to prevent death.
Grade 5 (Death): Death is assigned a Grade 5 severity.
Only the single adverse event that is assessed as the primary cause of death should be assigned "grade 5" severity.

Toxicity Grading Scale for Healthy Adult and Adolescent Volunteers Enrolled in Preventive Vaccine Clinical Trials
Modified from FDA Guidance - September 2007

A. Tables for Clinical Abnormalities

**Local Reaction to Injectable Product**	**Mild (Grade 1)**	**Moderate (Grade 2)**	**Severe (Grade 3)**	**Potentially Life Threatening (Grade 4)**
Pain	Does not interfere with activity	Repeated use of non-narcotic pain reliever > 24 hours or interferes with activity	Any use of narcotic pain reliever or prevents daily activity	Hospitalization
Tenderness	Mild discomfort to touch	Discomfort with movement	Significant discomfort at rest	Hospitalization
[1]Erythema/Redness	2.5 – 5 cm	5.1 – 10 cm	> 10 cm	Necrosis or exfoliative dermatitis requiring medical attention
[2] Induration/Swelling	2.5 – 5 cm and does not interfere with activity	5.1 – 10 cm or interferes with activity	> 10 cm or prevents daily activity	Necrosis requiring medical attention
**[3] Vital Signs**	**Mild (Grade 1)**	**Moderate (Grade 2)**	**Severe (Grade 3)**	**Potentially Life Threatening (Grade 4)**
[4] Fever (°C)<br>(°F)	38.0 – 38.4<br>100.4 – 101.1	38.5 – 38.9<br>101.2 – 102.0	39.0 – 40<br>102.1 – 104	> 40<br>> 104
Tachycardia - beats per minute	101 – 115	116 – 130	> 130	Hospitalization for arrhythmia
[5] Bradycardia - beats per Minute	50 – 54	45 – 49	< 45	Hospitalization for arrhythmia
Hypertension (systolic) - mm Hg	141 – 150	151 – 155	> 155	Hospitalization for malignant hypertension
Hypertension (diastolic) - mm Hg	91 – 95	96 – 100	> 100	Hospitalization for malignant hypertension
Hypotension (systolic) – mm Hg	85 – 89 and symptomatic	80 – 84 and symptomatic and requiring oral fluids	< 80 and symptomatic and requiring IV fluids	Hospitalization for hypotensive shock
Respiratory Rate – breaths per minute	17 – 20	21 – 25	> 25	Intubation

1. In addition to grading the measured local reaction at the greatest single diameter, the measurement should be recorded as a continuous variable.
2. Induration/Swelling should be evaluated and graded using the functional scale as well as the actual measurement.
3. Subject should be at rest for all vital sign measurements.
4. Oral temperature; no recent hot or cold beverages or smoking.
5. When resting heart rate is between 60 – 100 beats per minute. Use clinical judgment when characterizing Bradycardia among some healthy subject populations, for example, conditioned athletes.

**Systemic (General)**	**Mild (Grade 1)**	**Moderate (Grade 2)**	**Severe (Grade 3)**	**Potentially Life Threatening (Grade 4)**
Nausea/vomiting	No interference with activity or 1 – 2 episodes/24 hours	Some interference with activity or > 2 episodes/24 hours	Prevents daily activity, requires outpatient IV hydration	Hospitalization for hypotensive shock
Diarrhea	2 – 3 loose stools or < 400 gms/24 hours	4 – 5 stools or 400 – 800 gms/24 hours	6 or more watery stools or > 800gms/24 hours or requires outpatient IV hydration	Hospitalization
Headache	No interference with activity	Repeated use of non-narcotic pain reliever > 24 hours or some interference with activity	Significant; any use of narcotic pain reliever or prevents daily activity	Hospitalization
Fatigue	No interference with activity	Some interference with activity	Significant; prevents daily activity	Hospitalization
Myalgia	No interference with activity	Some interference with activity	Significant; prevents daily activity	Hospitalization

**Systemic Illness**	**Mild (Grade 1)**	**Moderate (Grade 2)**	**Severe (Grade 3)**	**Potentially Life Threatening (Grade 4)**
Illness or clinical adverse event (as defined according to applicable regulations)	No interference with activity	Some interference with activity not requiring medical intervention	Prevents daily activity and requires medical intervention	Hospitalization

B. Tables for Laboratory Abnormalities

**Serum ***	**Mild (Grade 1)**	**Moderate (Grade 2)**	**Severe (Grade 3)**	**Potentially Life Threatening (Grade 4)**
Sodium – Hyponatremia mEq/L	132 – 134	130 – 131	125 – 129	< 125
Sodium – Hypernatremia mEq/L	144 – 145	146 – 147	148 – 150	> 150
Potassium – Hyperkalemia mEq/L	5.1 – 5.2	5.3 – 5.4	5.5 – 5.6	> 5.6
Potassium – Hypokalemia mEq/L	3.5 – 3.6	3.3 – 3.4	3.1 – 3.2	< 3.1
Glucose – Hypoglycemia mg/dL	65 – 69	55 – 64	45 – 54	< 45
Glucose – Hyperglycemia<br>Fasting – mg/dL<br>Random – mg/dL	 100 – 110<br>110 – 125	 111 – 125<br>126 – 200	 >125<br>>200	Insulin requirements or hyperosmolar coma
Blood Urea Nitrogen BUN mg/dL	23 – 26	27 – 31	> 31	Requires dialysis
Creatinine – mg/dL	1.5 – 1.7	1.8 – 2.0	2.1 – 2.5	> 2.5 or requires dialysis
Calcium – hypocalcemia mg/dL	8.0 – 8.4	7.5 – 7.9	7.0 – 7.4	< 7.0
Calcium – hypercalcemia mg/dL	10.5 – 11.0	11.1 – 11.5	11.6 – 12.0	> 12.0
Magnesium – hypomagnesemia mg/dL	1.3 – 1.5	1.1 – 1.2	0.9 – 1.0	< 0.9
Phosphorous – hypophosphatemia mg/dL	2.3 – 2.5	2.0 – 2.2	1.6 – 1.9	< 1.6
CPK – mg/dL	1.25–1.5 xULN**	1.6 – 3.0 x ULN	3.1 –10 x ULN	> 10 x ULN
Albumin – Hypoalbuminemia g/dL	2.8 – 3.1	2.5 – 2.7	< 2.5	--
Total Protein – Hypoproteinemia g/dL	5.5 – 6.0	5.0 – 5.4	< 5.0	--
Alkaline phosphate – increase by factor	1.1 – 2.0 x ULN	2.1 – 3.0 x ULN	3.1 – 10 x ULN	> 10 x ULN
Liver Function Tests –ALT, AST increase by factor	1.1 – 2.5 x ULN	2.6 – 5.0 x ULN	5.1 – 10 x ULN	> 10 x ULN
Bilirubin – when accompanied by any increase in Liver Function Test increase by factor	1.1 – 1.25 x ULN	1.26 – 1.5 x ULN	1.51 – 1.75 x ULN	> 1.75 x ULN
Bilirubin – when Liver Function Test is normal; increase by factor	1.1 – 1.5 x ULN	1.6 – 2.0 x ULN	2.0 – 3.0 x ULN	> 3.0 x ULN
Cholesterol	201 – 210	211 – 225	> 226	---
Pancreatic enzymes – amylase, lipase	1.1 – 1.5 x ULN	1.6 – 2.0 x ULN	2.1 – 5.0 x ULN	> 5.0 x ULN

* The laboratory values provided in the tables serve as guidelines and are dependent upon institutional normal parameters. Institutional normal reference ranges should be provided to demonstrate that they are appropriate.
**ULN" is the upper limit of the normal range.

Hematology *	Mild (Grade 1)	Moderate (Grade 2)	Severe (Grade 3)	Potentially Life Threatening (Grade 4)
Hemoglobin (Female) - gm/dL	11.0 – 12.0	9.5 – 10.9	8.0 – 9.4	< 8.0
Hemoglobin (Female) decrease from baseline value - gm/dL	not applicable	1.6 – 2.0	2.1 – 5.0	> 5.0
Hemoglobin (Male) - gm/dL	12.5 – 13.5	10.5 – 12.4	8.5 – 10.4	< 8.5
Hemoglobin (Male) decrease from baseline value – gm/dL	not applicable	1.6 – 2.0	2.1 – 5.0	> 5.0
WBC Increase - cell/mm^3	10,800 – 15,000	15,001 – 20,000	20,001 – 25, 000	> 25,000
WBC Decrease - cell/mm^3	2,500 – 3,500	1,500 – 2,499	1,000 – 1,499	< 1,000
Lymphocytes Decrease - cell/mm^3	750 – 1,000	500 – 749	250 – 499	< 250
Neutrophils Decrease - cell/mm^3	1,500 – 2,000	1,000 – 1,499	500 – 999	< 500
Eosinophils - cell/mm^3	650 – 1500	1501 - 5000	> 5000	Hypereosinophilic
Platelets Decreased - cell/mm^3	125,000 – 140,000	100,000 – 124,000	25,000 – 99,000	< 25,000
PT – increase by factor (prothrombin time)	1.10 x ULN**	1.11 – 1.20 x ULN	1.21 – 1.25 x ULN	> 1.25 ULN
PTT – increase by factor (partial thromboplastin time)	1.10 – 1.20 x ULN	1.21 – 1.4 x ULN	1.41 – 1.5 x ULN	> 1.5 x ULN
Fibrinogen increase - mg/dL	400 – 500	501 – 600	> 600	--
Fibrinogen decrease - mg/dL	150 – 200	125 – 149	100 – 124	< 100 or associated with gross bleeding or disseminated intravascular coagulation (DIC)

* The laboratory values provided in the tables serve as guidelines and are dependent upon institutional normal parameters. Institutional normal reference ranges should be provided to demonstrate that they are appropriate.
**ULN" is the upper limit of the normal range.
