# Supplementary material for: Safety and immunogenicity of investigational seasonal influenza hemagglutinin DNA vaccine followed by trivalent inactivated vaccine administered intradermally or intramuscularly in healthy adults: An open-label randomized phase 1 clinical trial
Source: PLoS One. 2019 Sep 18;14(9):e0222178. doi: 10.1371/journal.pone.0222178 (PMC6750650; doi:10.1371/journal.pone.0222178)
Supplement: S1 Table — (PDF) [file pone.0222178.s004.pdf]

S1 Table. Summary of solicited systemic reactogenicity after prime and boost vaccination

Symptoms Intensity	Group 1 DNA/IIV3 ID (N=53/N=51)[a]	Group 2 DNA/IIV3 IM (N=57/N=56)	Group 3 IIV3-IIV3 ID (N=50/N=45)	Group 4 IIV3-IIV3 IM (N=56/N=55)	Group 5 DNA/IIV3-IIV3 ID (N=48/N=45)	Group 6 DNA-IIV3/IIV3 IM (N=52/N=47)
**Prime Vaccination**						
**MALAISE**						
**None**	41 (77.4%)	42 (73.7%)	40 (80.0%)	47 (83.9%)	38 (79.2%)	43 (82.7%)
**Mild**	10 (18.9%)	10 (17.5%)	8 (16.0%)	7 (12.5%)	9 (18.8%)	9 (17.3%)
**Moderate**	2 (3.8%)	5 (8.8%)	2 (4.0%)	2 (3.6%)	1 (2.1%)	0 (0.0%)
**MYALGIA**						
**None**	49 (92.5%	51 (89.5%)	43 (86.0%)	51 (91.1%)	43 (89.6%)	45 (86.5%)
**Mild**	3 (5.7%)	4 (7.0%)	6 (12.0%)	4 (7.1%)	5 (10.4%)	7 (13.5%)
**Moderate**	1 (1.9%)	2 (3.5%)	31 (2.0%)	1 (1.8%)	0 (0.0%)	0 (0.0%)
**HEADACHE**						
**None**	38 (71.7%)	48 (84.2%)	40 (80.0%)	44 (78.6%)	32 (66.7%)	45 (86.5%)
**Mild**	15 (28.3%)	8 (14.0%)	9 (18.0%)	10 (17.9%)	13 (27.1%)	6 (11.5%)
**Moderate**	0 (0.0%)	1 (1.8%)	1 (2.0%)	2 (3.6%)	2 (4.2%)	1 (1.9%)
**Severe**	0 (0.0%)	0 (0.0%)	0 (0.0%)	0 (0.0%)	1 (2.1%)	0 (0.0%)
**CHILLS**						
**None**	51 (96.2%)	53 (93.0%)	47 (94.0%)	54 (96.4%)	46 (95.8%)	51 (98.1%)
**Mild**	1 (1.9%)	2 (3.5%)	2 (4.0%)	2 (3.6%)	2 (4.2%)	1 (1.9%)
**Moderate**	1 (1.9%)	2 (3.5%)	1 (2.0%)	0 (0.0%)	0 (0.0%)	0 (0.0%)
**NAUSEA**						
**None**	50 (94.3%)	54 (94.7%)	46 (92.0%)	54 (96.4%)	44 (91.7%)	52 (100.0%)
**Mild**	2 (3.8%)	1 (1.8%)	4 (8.0%)	2 (3.6%)	4 (8.3%)	0 (0.0%)
**Moderate**	1 (1.9%)	2 (3.5%)	0 (0.0%)	0 (0.0%)	0 (0.0%)	0 (0.0%)
**TEMPERATURE**						
**None**	52 (98.1%)	57 (100.0%)	49 (98.0%)	56 (100.0%)	47 (97.9%)	52 (100.0%)
**Mild**	0 (0.0%)	0 (0.0%)	1 (2.0%)	0 (0.0%)	1 (2.1%)	0 (0.0%)
**Missing**	1 (1.95)	0 (0.0%)	0 (0.0%)	0 (0.0%)	0 (0.0%)	0 (0.0%)
**ANY SYSTEMIC SYMPTOM[b]**						
**None**	29 (54.7%)	38 (66.7%)	33 (66.0%)	36 (64.3%)	25 (52.1%)	37 (71.2%)
**Mild**	22 (41.5%)	14 (24.6%)	15 (30.0%)	16 (28.6%)	20 (41.7%)	14 (26.9%)
**Moderate**	2 (3.8%)	5 (8.8%)	2 (4.0%)	4 (&.1%)	2 (4.2%)	1 (1.9%)
**Severe**	0 (0.0%)	0 (0.0%)	0 (0.0%)	0 (0.0%)	1 (2.1%)	0 (0.0%)
**Booster Vaccination**						
**MALAISE**						
**None**	45 (88.2%)	49 (87.5%)	35 (77.8%)	46 (83.6%)	39 (86.7%)	38 (80.9%)
**Mild**	5 (9.8%)	6 (10.7%)	9 (20.0%)	7 (12.7%)	4 (8.9%)	5 (10.6%)
**Moderate**	1 (2.0%)	1 (1.8%)	1 (2.2%)	2 (3.6%)	1 (2.2%)	4 (8.5%)
**Severe**	0 (0.0%)	0 (0.0%)	0 (0.0%)	0 (0.0%)	1 (2.2%)	0 (0.0%)
**MYALGIA**						

**None**	48 (94.1%)	51 (91.1%)	34 (75.6%)	51 (92.7%)	41 (91.1%)	43 (91.5%)
**Mild**	2 (3.9%)	5 (8.9%)	10 (22.2%)	4 (7.3%)	3 (6.7%)	1 (2.1%)
**Moderate**	1 (2.0%)	0 (0.0%)	1 (2.2%)	0 (0.0%)	1 (2.2%)	3 (6.4%)
**HEADACHE**						
**None**	44 (86.3%)	48 (85.7%)	33 (73.3%)	49 (89.1%)	39 (86.7%)	40 (85.1%)
**Mild**	6 (11.8%)	6 (10.7%)	11 (24.4%)	5 (9.1%)	2 (4.4%)	4 (8.5%)
**Moderate**	1 (2.0%)	2 (3.6%)	1 (2.2%)	1 (1.8%)	3 (6.7%)	3 (6.4%)
**Severe**	0 (0.0%)	0 (0.0%)	0 (0.0%)	0 (0.0%)	1 (2.2%)	0 (0.0%)
**CHILLS**						
**None**	49 (96.1%)	56 (100.0%)	41 (91.1%)	55 (100.0%)	43 (95.6%)	45 (95.7%)
**Mild**	2 (3.9%)	0 (0.0%)	2 (4.4%)	0 (0.0%)	1 (2.2%)	2 (4.3%)
**Moderate**	0 (0.0%)	0 (0.0%)	2 (4.4%)	0 (0.0%)	1 (2.2%)	0 (0.0%)
**NAUSEA**						
**None**	50 (98.0%)	55 (98.2%)	41 (91.1%)	52 (94.5%)	41 (91.1%)	45 (95.7%)
**Mild**	1 (2.0%)	1 (1.8%)	4 (8.9%)	1 (1.8%)	2 (4.4%)	2 (4.3%)
**Moderate**	0 (0.0%)	0 (0.0%)	0 (0.0%)	2 (3.6%)	1 (2.2%)	0 (0.0%)
**Severe**	0 (0.0%)	0 (0.0%)	0 (0.0%)	0 (0.0%)	1 (2.2%)	0 (0.0%)
**TEMPERATURE**						
**None**	49 (96.1%)	55 (98.2%)	44 (97.8%)	55 (100.0%)	44 (97.8%)	47 (100.0%)
**Mild**	0 (0.0%)	0 (0.0%)	0 (0.0%)	0 (0.0%)	1 (2.2%)	0 (0.0%)
**Moderate**	0 (0.0%)	0 (0.0%)	1 (2.2%)	0 (0.0%)	0 (0.0%)	0 (0.0%)
**Severe**	1 (2.0%)	0 (0.0%)	0 (0.0%)	0 (0.0%)	0 (0.0%)	0 (0.0%)
**Missing**	1 (2.0%)	1 (1.8%)	0 (0.0%)	0 (0.0%)	0 (0.0%)	0 (0.0%)
**ANY SYSTEMIC SYMPTOM[b]**						
**None**	42 (82.4%)	40 (71.4%)	29 (64.4%)	41 (74.5%)	35 (77.8%)	35 (74.5%)
**Mild**	6 (11.8%)	13 (23.2%)	14 (31.1%)	10 (18.2%)	5 (11.1%)	7 (14.9%)
**Moderate**	2 (3.9%)	3 (5.4%)	2 (4.4%)	4 (7.3%)	4 (8.9%)	5 (10.6%)
**Severe**	1 (2.0%)	0 (0.0%)	0 (0.0%)	0 (0.0%)	1 (2.2%)	0 (0.0%)
**Missing**	0 (0.0%)	0 (0.0%)	0 (0.0%)	0 (0.0%)	0 (0.0%)	0 (0.0%)

For participants reporting a symptom on multiple days, the symptom is counted once at the maximum severity.

[a](N=# subjects receiving prime vaccination/N=# subjects receiving boost vaccination)

[b]Any Systemic Symptoms displays the summation of all individually listed solicited systemic reactogenicity.
