# Supplementary material for: Safety and immunogenicity of investigational seasonal influenza hemagglutinin DNA vaccine followed by trivalent inactivated vaccine administered intradermally or intramuscularly in healthy adults: An open-label randomized phase 1 clinical trial
Source: PLoS One. 2019 Sep 18;14(9):e0222178. doi: 10.1371/journal.pone.0222178 (PMC6750650; doi:10.1371/journal.pone.0222178)
Supplement: S2 Table — (PDF) [file pone.0222178.s005.pdf]

S2 Table. Seroconversion rates for study groups as measured by HAI: % of subjects (95% CI)

HAI Antigen and Time point	Vaccine Regimen					
	DNA-IIV3		IIV3-IIV3		DNA/IIV3-IIV3	
	ID (n=51)[b]	IM (n=56)	ID (n=49)	IM (n=56)	ID (n=47)	IM (n=50)
A/California/07/2009 [A(H1N1)pdm09]						
**≥1:10 at baseline**	62.7 (48.1, 75.9)	57.1 (43.2, 70.3)	53.1 (38.3, 67.5)	53.6 (39.7, 67.0)	46.8 (32.1, 61.9)	62.0 (47.2, 75.3)
**≥1:40 at baseline**	45.1 (31.1, 59.7)	39.3 (26.5, 53.2)	34.7 (21.7, 49.6)	44.6 (31.1, 58.5)	36.2 (22.7, 51.5)	44.0 (30.0, 58.7)
**positive 3 weeks post prime[a]**	2.0 (0.0, 10.4)	1.8 (0.0 ,9.6)	44.9 (30.7, 59.8)	46.4 (33.0, 60.3)	34.0 (20.9, 49.3)	50.0 (35.5, 64.5)
**positive 3 weeks post-boost**	41.2 (27.6, 55.8)	43.6 (30.3, 57.7)	45.5 (30.4, 61.2)	50.9 (37.1, 64.6)	34.9 (21.0, 50.9)	59.6 (44.3, 73.6)
**positive 24 weeks post-boost**	31.3 (18.7, 46.3)	26.4 (15.3, 40.3)	34.1 (20.5, 49.9)	32.7 (20.3, 47.1)	27.9 (15.3, 43.7)	34.1 (20.5, 49.9)
A/Victoria/361/2011 (H3N2)						
**≥1:10 at baseline**	35.3 (22.4, 49.9)	41.1 (28.1, 55.0)	28.6 (16.6, 43.3)	21.4 (11.6, 34.4)	29.8 (17.3, 44.9)	26.0 (14.6, 40.3)
**≥1:40 at baseline**	25.5 (14.3, 39.6)	21.4 (11.6, 34.4)	16.3 (7.3, 29.7)	16.1 (7.6, 28.3)	17.0 (7.6, 30.8)	20.0 (10.0, 33.7)
**positive 3 weeks post prime**	3.9 (0.5, 13.5)	0.0 (0.0, 6.4)	40.8 (27.0, 55.8)	60.7 (46.8, 73.5)	36.2 (22.7, 51.5)	56.0 (41.3, 70.0)
**positive 3 weeks post-boost**	60.8 (46.1, 74.2)	50.9 (37.1, 64.6)	60.0 (44.3, 74.3)	80.0 (67.0, 89.6)	55.8 (39.9, 70.9)	70.2 (55.1, 82.7)
**positive 24 weeks post-boost**	43.8 (29.5, 58.8)	35.8 (23.1, 50.2)	43.2 (28.3, 59.0)	44.2 (30.5, 58.7)	30.2 (17.2, 46.1)	56.8 (41.0, 71.7)
B/Wisconsin/1/2010						
**≥1:10 at baseline**	35.3 (22.4, 49.9)	30.4 (18.8, 44.1)	20.4 (10.2, 34.3)	19.6 (10.2, 32.4)	25.5 (13.9, 40.3)	28.0 (16.2, 42.5)
**≥1:40 at baseline**	23.5 (12.8, 37.5)	17.9 (8.9, 30.4)	14.3 (5.9, 27.2)	16.1 (7.6, 28.3)	21.3 (10.7, 35.7)	20.0 (10.0, 33.7)
**positive 3 weeks post prime**	5.9 (1.2, 16.2)	1.8 (0.0, 9.6)	30.6 (18.3, 45.4)	55.4 (41.5, 68.7)	23.4 (12.3, 38.0)	54.0 (39.3, 68.2)
**positive 3 weeks post-boost**	39.2 (25.8, 53.9)	32.7 (20.7, 46.7)	26.7 (14.6, 41.9)	56.4 (42.3, 69.7)	34.9 (21.0, 50.9)	46.8 (32.1, 61.9)
**positive 24 weeks post-boost**	27.1 (15.3, 41.8)	18.9 (9.4, 32.0)	20.5 (9.8, 35.3)	25.0 (14.0, 38.9)	23.3 (11.8, 38.6)	29.5 (16.8, 45.2)
B/Texas/6/2011						
**≥1:10 at baseline**	56.0 (41.3, 70.0)	42.9 (29.7, 56.8)	34.7 (21.7, 49.6)	38.2 (25.4, 52.3)	36.2 (22.7, 51.5)	40.0 (26.4, 54.8)
**≥1:40 at baseline**	26.0 (14.6, 40.3)	19.6 (10.2, 32.4)	16.3 (7.3, 29.7)	16.4 (7.8, 28.8)	21.3 (10.7, 35.7)	20.0 (10.0, 33.7)
**positive 3 weeks post prime**	4.0 (0.5, 13.7)	0.0 (0.0, 6.4)	26.5 (14.9, 41.1)	58.2 (44.1, 71.3)	25.5 (13.9, 40.3)	51.0 (36.3, 65.6)
**positive 3 weeks post-boost**	48.0 (33.7, 62.6)	49.1 (35.4, 62.9)	22.7 (11.5, 37.8)	40.7 (27.6, 55.0)	25.6 (13.5, 41.2)	42.2 (27.7, 57.8)
**positive 24 weeks post-boost**	25.5 (13.9, 40.3)	20.8 (10.8, 34.1)	11.6 (3.9, 25.1)	15.7 (7.0, 28.6)	25.6 (13.5, 41.2)	27.3 (15.0, 42.8)
A/Texas/50/2012 (H3N2)						
**≥1:10 at baseline**	62.7 (48.1, 75.9)	69.6 (55.9, 81.2)	57.1 (42.2, 71.2)	50.0 (36.3, 63.7)	56.7 (43.2, 73.0)	62.0 (47.2, 75.3)
**≥1:40 at baseline**	47.1 (32.9, 61.5)	42.9 (29.7, 56.8)	40.8 (27.0, 55.8)	41.1 (28.1, 55.0)	41.3 (27.0, 56.8)	46.0 (31.8, 60.7)
**positive 3 weeks post prime**	7.8 (2.2, 18.9)	5.4 (1.1, 14.9)	33.3 (20.4, 48.4)	57.1 (43.2, 70.3)	32.6 (19.5, 48.0)	42.0 (28.2, 56.8)
**positive 3 weeks post-boost**	49.0 (34.8, 63.4)	41.8 (28.7, 55.9)	40.0 (25.7, 55.7)	65.5 (51.4, 77.8)	37.2 (23.0, 53.3)	55.3 (40.1, 69.8)
**positive 24 weeks post-boost**	33.3 (20.4, 48.4)	22.6 (12.3, 36.2)	29.5 (16.8, 45.2)	40.4 (27.0, 54.9)	23.3 (11.8, 38.6)	36.4 (22.4, 52.2)
B/Massachusetts/2/2012						
**≥1:10 at baseline**	37.3 (24.1, 51.9)	32.1 (20.3, 46.0)	22.4 (11.8, 36.6)	39.3 (26.5, 53.2)	29.8 (17.3, 44.9)	30.0 (17.9, 44.6)
**≥1:40 at baseline**	17.6 (8.4, 30.9)	14.3 (6.4, 26.2)	16.3 (7.3, 29.7)	21.4 (11.6, 34.4)	17.0 (7.6, 30.8)	8.0 (2.2, 19.2)
**positive 3 weeks post prime**	2.0 (0.0, 10.4)	0.0 (0.0, 6.4)	22.4 (11.8, 36.6)	41.1 (28.1, 55.0)	19.1 (9.1, 33.3)	52.0 (37.4, 66.3)
**positive 3 weeks post-boost**	33.3 (20.8, 47.9)	30.9 (19.1, 44.8)	11.1 (3.7, 24.1)	25.5 (14.7, 39.0)	20.9 (10.0, 36.0)	27.7 (15.6, 42.6)
**positive 24 weeks post-boost**	14.6 (6.1, 27.8)	18.9 (9.4, 32.0)	2.3 (0.1, 12.0)	13.5 (5.6, 25.8)	9.3 (2.6, 22.1)	11.4 (3.8, 24.6)

[a]Positive immune response defined as four-fold increase if reference titer (baseline or pre-boost) is ≥1:10, or ≥1:40 if reference titer is <1:10

[b]Number of subjects per group is based on the number of samples run at baseline for A/California/07/09
