# Supplementary material for: Safety and immunogenicity of investigational seasonal influenza hemagglutinin DNA vaccine followed by trivalent inactivated vaccine administered intradermally or intramuscularly in healthy adults: An open-label randomized phase 1 clinical trial
Source: PLoS One. 2019 Sep 18;14(9):e0222178. doi: 10.1371/journal.pone.0222178 (PMC6750650; doi:10.1371/journal.pone.0222178)
Supplement: S3 Table — (PDF) [file pone.0222178.s006.pdf]

S3 Table. Magnitude of antibody responses for study groups as measured by HAI: GMT (95% CI)

HAI Antigen and Time point	Vaccine Regimen					
	DNA-IIV3		IIV3-IIV3		DNA/IIV3-IIV3	
	ID (n=51)[a]	IM (n=56)	ID (n=49)	IM (n=56)	ID (n=47)	IM (n=50)
A/California/07/2009 [A(H1N1)pdm09]						
**baseline**	22.9 (15.7, 33.5)	20.8 (14.3, 30.2)	16.8 (11.5, 24.4)	20.9 (14.1, 30.9)	16.9 (11.0, 25.8)	27.9 (17.4, 44.7)
**3 weeks post prime**	22.1 (15.2, 32.2)	19.0 (13.5, 26.8)	94.1 (60.4, 146.7)	125.7 (85.1, 185.7)	69.5 (44.8, 108.0)	168.0 (114.3, 246.8)
**3 weeks post boost**	108.6 (77.1, 153.0)	86.3 (62.1, 119.9)	78.7 (51.3, 121.0)	111.0 (74.5, 165.4)	73.8 (47.6, 114.3)	142.2 (98.9, 204.4)
**24 weeks post boost**	62.1 (40.6, 95.1)	51.6 (34.5, 77.3)	50.3 (32.8, 77.1)	60.5 (38.7, 94.4)	45.5 (28.7, 72.2)	67.3 (43.4, 104.2)
A/Victoria/361/2011 (H3N2)						
**baseline**	11.9 (8.3, 16.9)	12.4 (8.7, 17.7)	10.6 (7.0, 15.9)	8.8 (6.5, 11.8)	10.2 (7.1, 14.7)	10.8 (7.2, 16.2)
**3 weeks post prime**	11.5 (8.4, 15.6)	10.7 (7.7, 15.0)	53.8 (33.6, 86.3)	80.5 (54.0, 120.0)	40.0 (24.9, 64.3)	97.1 (59.1, 159.6)
**3 weeks post boost**	71.8 (48.7, 105.8)	65.4 (43.2, 98.9)	84.4 (54.0, 132.1)	111.7 (77.0, 162.2)	68.5 (43.4, 107.9)	114.0 (71.1, 182.8)
**24 weeks post boost**	49.0 (33.0, 72.6)	45.6 (31.2, 66.6)	43.6 (28.5, 66.9)	44.2 (29.5, 66.2)	34.6 (21.3, 56.1)	60.7 (39.2, 93.7)
B/Wisconsin/1/2010						
**baseline**	11.5 (8.1, 16.4)	9.8 (7.3, 13.1)	8.5 (6.1, 11.8)	8.5 (6.3, 11.3)	9.9 (6.8, 14.3)	9.9 (7.3, 13.5)
**3 weeks post prime**	13.2 (9.3, 18.7)	9.6 (7.1, 12.8)	27.7 (18.8, 40.9)	57.6 (38.2, 86.9)	28.3 (17.9, 44.7)	66.8 (43.2, 103.4)
**3 weeks post boost**	45.4 (31.5, 65.4)	36.3 (24.7, 53.4)	21.3 (14.1, 32.0)	48.6 (33.8, 69.9)	37.2 (24.4, 56.7)	43.7 (29.3, 65.1)
**24 weeks post boost**	29.1 (19.5, 43.5)	21.5 (14.6, 31.6)	16.8 (11.4, 24.8)	26.3 (18.0, 38.5)	19.8 (12.8, 30.7)	30.8 (20.1, 47.4)
B/Texas/6/2011						
**baseline**	14.7 (10.6, 20.6)	10.9 (8.2, 14.5)	10.7 (7.7, 15.0)	10.5 (7.9, 13.9)	11.8 (8.1, 17.0)	10.7 (8.0, 14.4)
**3 weeks post prime**	16.5 (11.9, 22.9)	11.3 (8.5, 15.1)	34.0 (23.2, 49.8)	62.6 (41.7, 94.0)	33.3 (21.6, 51.2)	70.4 (45.3, 109.6)
**3 weeks post boost**	73.6 (50.5, 107.3)	51.1 (33.4, 78.4)	24.0 (15.8, 36.3)	39.0 (26.6, 57.2)	31.7 (20.6, 48.8)	42.5 (27.7, 65.4)
**24 weeks post boost**	37. 4 (25.4, 55.2)	25.8 (17.2, 38.8)	16.0 (10.6, 24.1)	22.3 (15.2, 32.7)	21.3 (13.8, 33.0)	23.0 (15.4, 34.5)
A/Texas/50/2012 (H3N2)						
**baseline**	24.5 (16.2, 37.1)	27.1 (18.6, 39.4)	23.9 (15.0, 37.9)	20.6 (13.6, 31.4)	24.0 (14.6, 39.3)	28.3 (17.8, 44.9)
**3 weeks post prime**	34.4 (22.9, 51.9)	32.8 (22.7, 47.4)	98.6 (67.1, 145.0)	125.7 (85.4, 185.1)	68.8 (43.0, 110.0)	145.2 (91.5, 230.5)
**3 weeks post boost**	121.9 (85.5, 173.9)	105.8 (73.8, 151.5)	142.9 (95.8, 213.2)	154.1 (108.3, 219.1)	101.9 (66.1, 157.1)	182.3 (118.8, 279.6)
**24 weeks post boost**	59.9 (39.5, 90.8)	56.2 (38.3, 82.5)	63.7 (40.7, 99.5)	68.2 (45.0, 103.2)	53.0 (32.6, 86.2)	85.9 (53.7, 137.3)
B/Massachusetts/2/2012						
**baseline**	9.7 (7.5, 12.5)	8.5 (6.7, 10.7)	8.6 (6.5, 11.5)	11.0 (8.3, 14.7)	9.4 (7.0, 12.7)	8.4 (6.5, 10.6)
**3 weeks post prime**	10.6 (8.1, 13.8)	9.3 (7.4, 11.8)	24.6 (17.7, 34.1)	39.0 (27.2, 56.1)	21.7 (14.9, 31.5)	48.2 (33.8, 68.8)
**3 weeks post boost**	31.7 (23.0, 43.9)	27.8 (19.4, 39.8)	11.2 (7.8, 16.1)	21.3 (14.8, 30.7)	15.6 (10.3, 23.5)	19.7 (13.5, 28.8)
**24 weeks post boost**	18.6 (13.4, 25.8)	14.8 (10.5, 20.9)	9.0 (6.5, 12.5)	13.1 (9.1, 18.7)	12.6 (8.6, 18.5)	13.1 (8.9, 19.2)

[a]Number of subjects per group is based on the number of samples run at baseline for A/California/07/09
