# Supplementary material for: Safety and immunogenicity of investigational seasonal influenza hemagglutinin DNA vaccine followed by trivalent inactivated vaccine administered intradermally or intramuscularly in healthy adults: An open-label randomized phase 1 clinical trial
Source: PLoS One. 2019 Sep 18;14(9):e0222178. doi: 10.1371/journal.pone.0222178 (PMC6750650; doi:10.1371/journal.pone.0222178)
Supplement: S4 Table — (PDF) [file pone.0222178.s007.pdf]

S4 Table. Magnitude of antibody responses for age subgroups as measured by HAI: GMT (95% CI)

HAI Antigen and Time Point	Vaccine Regimen					
	DNA-IIV3		IIV3-IIV3		DNA/IIV3-IIV3	
	ID (n=51)[a]	IM (n=56)	ID (n=49)	IM (n=56)	ID (n=47)	IM (n=50)
A/California/07/2009 A(H1N1)pdm09						
**Baseline**						
**(18-50 years)**	25.1 (15.8, 39.9)	25.9 (15.3, 43.7)	14.9 (9.3, 24.0)	25.4 (15.4, 41.9)	21.1 (11.7, 38.3)	35.0 (18.2, 67.1)
**(51-70 years)**	17.5 (8.5, 36.1)	14.4 (8.7, 23.9)	20.8 (10.8, 40.2)	15.1 (7.8, 29.2)	10.9 (6.6, 18.0)	19.3 (9.8, 38.1)
**3 weeks post prime**				$p=0.012$	$p=0.002$	
**(18-50 years)**	24.9 (16.0, 38.6)	22.1 (13.6, 35.8)	94.1 (53.3, 166.0)	189.3 (125.9, 284.6)	107.0 (61.1, 187.5)	209.2 (127.1, 344.5)
**(51-70 years)**	15.7 (7.2, 34.5)	14.9 (9.3, 23.8)	94.2 (42.8, 207.4)	63.5 (30.2, 133.4)	30.2 (17.3, 52.7)	117.3 (62.7, 219.7)
**3 weeks post boost**				$p=0.018$	$p=0.002$	
**(18-50 years)**	125.1 (85.9, 182.1)	100.1 (63.3, 158.3)	77.1 (43.2, 137.5)	165.0 (108.2, 251.4)	116.0 (67.8, 198.3)	154.2 (93.1, 255.4)
**(51-70 years)**	71.9 (31.2, 165.7)	67.8 (42.5, 108.2)	81.8 (40.8, 163.9)	58.5 (27.4, 124.6)	31.7 (17.3, 58.4)	126.2 (72.3, 220.3)
**24 weeks post boost**		$p=0.039$		$p=0.003$	$p=0.002$	
**(18-50 years)**	71.0 (42.3, 119.4)	71.0 (40.7, 124.0)	51.9 (29.1, 92.6)	101.5 (59.9, 172.1)	72.5 (41.0, 128.1)	91.4 (53.2, 157.1)
**(51-70 years)**	43.3 (19.5, 96.3)	31.7 (18.3, 55.1)	47.6 (24.0, 94.3)	26.4 (13.2, 52.9)	19.1 (10.0, 36.4)	43.2 (20.5, 90.9)
A/Victoria/361/2011 (H3N2)						
**Baseline**			$p=0.032$			
**(18-50 years)**	13.3 (8.5, 20.8)	14.9 (9.6, 23.1)	13.8 (7.8, 24.6)	10.3 (6.7, 15.7)	11.2 (6.6, 18.9)	10.7 (6.3, 18.2)
**(51-70 years)**	8.5 (5.2, 14.0)	9.2 (5.0, 17.0)	6.4 (4.1, 9.9)	6.7 (4.6, 9.8)	8.6 (5.6, 13.2)	11.0 (5.4, 22.1)
**3 weeks post prime**	$p=0.035$					
**(18-50 years)**	13.3 (9.0, 19.7)	13.3 (8.8, 20.2)	54.8 (28.5, 105.1)	90.1 (54.1, 149.9)	48.4 (25.9, 90.2)	86.5 (43.9, 170.4)
**(51-70 years)**	7.5 (5.0, 11.0)	7.4 (4.2, 13.1)	52.1 (26.5, 102.6)	66.7 (33.4, 133.3)	27.7 (13.0, 59.1)	117.3 (54.3, 253.8)
**3 weeks post boost**	$p=0.01$			$p=0.003$		
**(18-50 years)**	97.8 (64.6, 148.1)	76.0 (44.6, 129.6)	117.4 (67.6, 203.8)	177.2 (121.1, 259.3)	94.0 (55.1, 160.1)	143.1 (75.5, 271.4)
**(51-70 years)**	29.0 (12.8, 65.8)	51.2 (25.5, 103.0)	49.0 (23.0, 104.7)	53.0 (26.9, 104.3)	37.9 (16.3, 88.2)	81.5 (39.0, 170.0)
**24 weeks post boost**	$p=0.013$	$p=0.014$		$p<0.001$		
**(18-50 years)**	64.3 (404., 102.5)	67.3 (43.9, 103.0)	48.2 (26.7, 86.8)	71.8 (42.7, 120.6)	42.6 (23.3, 77.6)	78.9 (43.3, 143.6)
**(51-70 years)**	23.5 (12.1, 45.4)	25.2 (13.0, 49.0)	36.7 (19.3, 69.7)	20.3 (12.1, 34.2)	23.5 (9.8, 56.5)	41.6 (22.0, 78.6)
B/Wisconsin/1/2010						
**Baseline**				$p=0.03$		
**(18-50 years)**	12.6 (8.2, 19.5)	11.7 (7.7, 17.9)	9.9 (6.2, 15.9)	10.4 (6.8, 16.0)	11.1 (6.7, 18.2)	12.0 (7.7, 19.0)
**(51-70 years)**	8.8 (4.6, 16.6)	7.2 (5.0, 10.4)	6.4 (4.7, 8.7)	6.0 (4.6, 7.8)	7.9 (4.4, 14.2)	7.2 (5.1, 10.1)
**3 weeks post prime**	$p=0.026$	$p=0.013$	$p=0.001$	$p=0.008$	$p=0.013$	
**(18-50 years)**	16.1 (10.6, 24.4)	12.2 (8.0, 18.7)	42.2 (26.4, 67.6)	90.1 (58.5, 138.8)	40.4 (22.1, 74.1)	77.4 (47.3, 126.5)
**(51-70 years)**	7.5 (4.3, 13.0)	6.4 (4.8, 8.5)	12.5 (7.1, 21.9)	27.4 (12.7, 59.0)	14.1 (7.8, 25.7)	52.6 (21.8, 126.7)
**3 weeks post boost**	$p=0.002$	$p<0.001$	$p=0.017$	$p=0.003$	$p=0.001$	
**(18-50 years)**	65.9 (46.2, 93.9)	61.8 (38.4, 99.5)	30.1 (17.3, 52.3)	76.0 (52.8, 109.5)	60.2 (37.3, 97.1)	59.4 (38.0, 93.0)
**(51-70 years)**	15.3 (6.9, 33.9)	15.4 (9.4, 25.2)	12.0 (7.0, 20.5)	23.6 (12.1, 46.1)	15.2 (8.0, 28.8)	27.8 (13.3, 5.1)
**24 weeks post boost**	$p=0.001$	$p<0.001$	$p=0.007$	$p=0.008$	$p=0.003$	
**(18-50 years)**	42.9 (27.9, 65.9)	38.7 (23.3, 64.3)	23.8 (14.0, 40.3)	39.1 (25.0, 61.2)	30.1 (17.3, 52.5)	36.0 (22.0, 58.7)
**(51-70 years)**	10.3 (5.0, 21.2)	8.8 (6.1, 12.5)	9.2 (5.8, 14.6)	13.9 (7.4, 26.1)	9.1 (5.1, 16.2)	24.7 (10.7, 56.9)
B/Texas/6/2011						
**Baseline**		$p=0.04$	$p=0.009$			
**(18-50 years)**	16.4 (10.9, 24.5)	13.3 (8.9, 20.1)	13.8 (8.6, 22.2)	12.4 (8.4, 18.3)	14.1 (8.7, 23.0)	10.9 (7.2, 16.6)

**(51-70 years)**	10.6 (5.6, 19.9)	7.8 (5.6, 8.8)	6.7 (5.0, 8.8)	8.1 (5.5, 11.9)	8.2 (4.6, 14.6)	10.4 (6.7, 16.1)
**3 weeks post prime**<br>**(18-50 years)**<br>**(51-70 years)**	 19.1 (13.0, 28.2)<br>10.3 (5.9, 19.9)	*p=0.03*<br>14.0 (9.2, 21.4)<br>7.9 (5.9, 10.8)	*p=0.008*<br>48.1 (29.9, 77.2)<br>17.7 (9.9, 31.6)	*p=0.036*<br>90.4 (59.9, 136.6)<br>34.5 (15.2, 77.9)	*p=0.002*<br>50.6 (29.3, 87.3)<br>14.8 (8.4, 25.6)	 88.5 (53.1, 147.3)<br>47.6 (20.0, 113.0)
**3 weeks post boost**<br>**(18-50 years)**<br>**(51-70 years)**	*p=0.003*<br>105.2 (72.9, 151.7)<br>23.8 (10.1, 56.1)	*p<0.001*<br>94.2 (55.8, 158.8)<br>19.0 (11.1, 32.5)	*p=0.005*<br>37.0 (21.8, 62.8)<br>12.0 (6.7, 21.5)	*p=0.018*<br>56.6 (36.2, 88.3)<br>21.7 (11.2, 42.3)	*p<0.001*<br>55.9 (34.2, 91.2)<br>11.0 (6.3, 19.0)	 59.5 (36.5, 97.2)<br>25.7 (11.7, 56.6)
**24 weeks post boost**<br>**(18-50 years)**<br>**(51-70 years)**	*p=0.004*<br>52.3 (34.5, 79.2)<br>14.1 (6.7, 29.8)	*p<0.001*<br>46.0 (26.7, 79.4)<br>10.7 (7.1, 16.0)	*p=0.03*<br>21.9 (12.5, 38.2)<br>9.4 (5.4, 16.3)	*p=0.005*<br>34.2 (21.8, 53.7)<br>11.5 (6.2, 21.4)	*p<0.001*<br>34.9 (20.3, 60.1)<br>8.5 (5.1, 14.3)	 28.3 (17.1, 46.8)<br>17.1 (8.5, 34.5)
A/Texas/50/2012 (H3N2)						
**Baseline**<br>**(18-50 years)**<br>**(51-70 years)**	 24.4 (14.4, 40.8)<br>25.4 (13.0, 49.7)	 31.5 (19.7, 50.5)<br>21.0 (10.8, 40.8)	 28.9 (15.4, 54.1)<br>16.6 (8.5, 32.7)	 26.9 (15.6, 46.6)<br>13.2 (6.9, 25.5)	 27.0 (13.7, 53.4)<br>19.2 (9.4, 39.1)	 27.7 (14.9, 51.5)<br>29.3 (13.8, 62.2)
**3 weeks post prime**<br>**(18-50 years)**<br>**(51-70 years)**	 38.9 (23.5, 64.5)<br>24.1 (11.9, 48.9)	 39.6 (25.6, 61.2)<br>24.0 (12.0, 48.0)	 93.6 (54.5, 160.7)<br>108.6 (63.7, 185.3)	 140.7 (87.5, 226.1)<br>104.2 (51.2, 212.0)	 86.7 (47.8, 157.4)<br>44.6 (20.0, 99.2)	 148.0 (81.8, 267.5)<br>140.8 (62.5 , 317.5)
**3 weeks post boost**<br>**(18-50 years)**<br>**(51-70 years)**	*p=0.004*<br>167.5 (115.6, 242.7)<br>48.2 (23.1, 100.4)	 119.1 (74.2, 191.0)<br>87.4 (48.4, 157.6)	 166.1 (100.4, 274.7)<br>111.6 (54.7, 227.8)	*p=0.02*<br>212.8 (138.8, 326.5)<br>91.3 (51.0, 163.5)	 132.9 (80.6, 219.0)<br>62.0 (26.6, 144.8)	 218.0 (124.4, 382.1)<br>140.0 (68.8, 284.6)
**24 weeks post boost**<br>**(18-50 years)**<br>**(51-70 years)**	*p=0.005*<br>84.9 (53.3, 135.2)<br>23.5 (11.1, 49.6)	*p=0.009*<br>84.5 (53.8, 132.6)<br>30.2 (16.0, 57.0)	 72.5 (40.9, 128.3)<br>50.8 (23.0, 112.0)	*p=0.001*<br>114.4 (70.6, 185.2)<br>29.8 (15.7, 56.4)	 70.7 (39.3, 127.2)<br>31.0 (12.8, 75.3)	 101.7 (54.4, 190.1)<br>67.3 (31.2, 145.2)
B/Massachusetts/2/2012						
**Baseline**<br>**(18-50 years)**<br>**(51-70 years)**	 10.6 (7.7, 14.5)<br>7.5 (4.8, 11.5)	*p=0.019*<br>10.1 (7.2, 14.2)<br>6.3 (5.1, 7.8)	 9.9 (6.7, 14.7)<br>6.7 (4.5, 9.9)	*p=0.044*<br>13.6 (9.3, 19.9)<br>7.8 (5.2, 11.6)	 10.9 (7.2, 16.5)<br>7.1 (4.7, 10.5)	 8.6 (6.1, 12.2)<br>7.9 (5.6, 11.2)
**3 weeks post prime**<br>**(18-50 years)**<br>**(51-70 years)**	 12.0 (8.6, 16.7)<br>7.3 (4.8, 11.0)	*p=0.002*<br>11.7 (8.3, 16.5)<br>6.4 (5.3, 7.7)	*p=0.038*<br>31.5 (21.2, 46.9)<br>15.3 (8.7, 27.2)	*p=0.01*<br>57.7 (39.7, 83.8)<br>20.3 (10.2, 40.6)	*p<0.001*<br>32.0 (19.8, 51.6)<br>10.2 (6.7, 15.7)	 55.3 (36.0, 85.1)<br>38.6 (20.0, 74.5)
**3 weeks post boost**<br>**(18-50 years)**<br>**(51-70 years)**	*p=0.004*<br>41.9 (29.6, 59.3)<br>14.1 (7.5, 26.8)	*p<0.001*<br>47.6 (30.4, 74.4)<br>11.6 (7.7, 17.4)	 14.0 (8.5, 22.9)<br>7.8 (4.7, 12.9)	*p=0.022*<br>29.5 (18.6, 46.6)<br>12.6 (7.1, 22.5)	*p=0.004*<br>22.6 (13.2, 38.9)<br>7.8 (4.7, 12.7)	 24.1 (15.1, 38.4)<br>14.7 (7.5, 28.7)
**24 weeks post boost**<br>**(18-50 years)**<br>**(51-70 years)**	*p=0.002*<br>24.6 (16.9, 35.8)<br>8.8 (5.1, 14.9)	*p<0.001*<br>24.0 (15.0, 38.5)<br>7.1 (5.2, 9.6)	 10.1 (6.4, 16.0)<br>7.4 (4.7, 11.7)	*p=0.025*<br>17.6 (10.8, 28.6)<br>8.1 (5.0, 13.2)	*p=0.013*<br>17.0 (10.2, 28.4)<br>7.2 (4.6, 11.4)	 14.3 (9.0, 22.9)<br>11.4 (5.7, 23.0)

[a]Number of subjects per group is based on the number of samples run at baseline for A/California/07/09

p values were determined by Fisher's exact test, indicate comparisons between the age subgroups at each time point, and are only displayed when $p<0.05$.
