# Supplementary material for: Safety and immunogenicity of investigational seasonal influenza hemagglutinin DNA vaccine followed by trivalent inactivated vaccine administered intradermally or intramuscularly in healthy adults: An open-label randomized phase 1 clinical trial
Source: PLoS One. 2019 Sep 18;14(9):e0222178. doi: 10.1371/journal.pone.0222178 (PMC6750650; doi:10.1371/journal.pone.0222178)
Supplement: S5 Table — (PDF) [file pone.0222178.s008.pdf]

S5 Table. Seroconversion rates for age subgroups as measured by HAI: % of subjects (95% CI)

HAI Antigen and Time point	Vaccine Regimen					
	DNA-IIV3		IIV3-IIV3		DNA/IIV3-IIV3	
	ID (n=51)[b]	IM (n=56)	ID (n=49)	IM (n=56)	ID (n=47)	IM (n=50)
A/California/07/2009 [A(H1N1)pdm09]						
**≥1:10 at baseline**						
**(18-50 years)**	63.2 (46.0, 78.2)	60.0 (42.1, 76.1)	50.0 (31.9, 68.1)	60.0 (42.1, 76.1)	48.4 (30.2, 66.9)	64.5 (45.4, 80.8)
**(51-70 years)**	61.5 (31.6, 86.1)	52.4 (29.8, 74.3)	58.8 (32.9, 81.6)	42.9 (21.8, 66.0)	43.8 (19.8, 70.1)	57.9 (33.5, 79.7)
**≥1:40 at baseline**						
**(18-50 years)**	50.0 (33.4, 66.6)	45.7 (28.8, 63.4)	31.1 (16.1, 50.0)	51.4 (34.0, 68.6)	41.9 (24.5, 60.9)	51.6 (33.1, 69.8)
**(51-70 years)**	30.8 (9.1, 61.4)	28.6 (11.3, 52.2)	41.2 (18.4, 67.1)	33.3 (14.6, 57.0)	25.0 (7.3, 52.4)	31.6 (12.6, 56.6)
**positive 3 weeks post prime[a]**						
**(18-50 years)**	2.6 (0.1, 13.8)	0.0 (0.0, 10.0)	46.9 (29.1, 65.3)	54.3 (36.6, 71.2)	41.9 (24.5, 60.9)	45.2 (27.3, 64.0)
**(51-70 years)**	0.0 (0.0, 24.7)	4.8 (0.1, 23.8)	41.2 (18.4, 67.1)	33.3 (14.6, 57.0)	18.8 (4.0, 45.6)	57.9 (33.5, 79.7)
**positive 3 weeks post-boost**						
**(18-50 years)**	44.7 (28.6, 61.7)	38.2 (22.2, 56.4)	50.0 (30.6, 69.4)	61.8 (43.6, 77.8)	39.3 (21.5, 59.4)	57.1 (37.2, 75.5)
**(51-70 years)**	30.8 (9.1, 61.4)	52.4 (29.8, 74.3)	37.5 (15.2, 64.6)	33.3 (14.6, 57.0)	26.7 (7.8, 55.1)	63.2 (38.4, 83.7)
**positive 24 weeks post-boost**				*p=0.007*		
**(18-50 years)**	34.3 (19.1, 52.2)	31.3 (16.1, 50.0)	39.3 (21.5, 59.4)	46.9 (29.1, 65.3)	32.1 (15.9, 52.4)	42.3 (23.4, 63.1)
**(51-70 years)**	23.1 (5.0, 53.8)	19.0 (5.4, 41.9)	25.0 (7.3, 52.4)	10.0 (1.2, 31.7)	20.0 (4.3, 48.1)	22.2 (6.4, 47.6)
A/Victoria/361/2011 (H3N2)						
**≥1:10 at baseline**						
**(18-50 years)**	36.8 (21.8, 54.0)	48.6 (31.4, 66.0)	37.5 (21.1, 56.3)	28.6 (14.6, 46.3)	29.0 (14.2, 48.0)	25.8 (11.9, 44.6)
**(51-70 years)**	30.8 (9.1, 61.4)	28.6 (11.3, 52.2)	11.8 (1.5, 36.4)	9.5 (1.2, 30.4)	31.3 (11.0, 58.7)	26.3 (9.1, 51.2)
**≥1:40 at baseline**						
**(18-50 years)**	28.9 (15.4, 45.9)	28.6 (14.6, 46.3)	21.9 (9.3, 40.0)	20.0 (8.4, 36.9)	19.4 (7.5, 37.5)	19.4 (7.5, 37.5)
**(51-70 years)**	15.4 (1.9, 45.4)	9.5 (1.2, 30.4)	5.9 (0.1, 28.7)	9.5 (1.2, 30.4)	12.5 (1.6, 38.3)	21.1 (6.1, 45.6)
**positive 3 weeks post prime**						
**(18-50 years)**	5.3 (0.6, 17.7)	0.0 (0.0, 10.0)	34.4 (18.6, 53.2)	57.1 (39.4, 73.7)	41.9 (24.5, 60.9)	58.1 (39.1, 75.5)
**(51-70 years)**	0.0 (0.0, 24.7)	0.0 (0.0, 16.1)	52.9 (27.8, 77.0)	66.7 (43.0, 85.4)	25.0 (7.3, 52.4)	52.6 (28.9, 75.6)
**positive 3 weeks post-boost**						
**(18-50 years)**	65.8 (48.6, 80.4)	50.0 (32.4, 67.6)	60.7 (40.6, 78.5)	85.3 (68.9, 95.0)	60.7 (40.6, 78.5)	78.6 (59.0, 91.7)
**(51-70 years)**	46.2 (19.2, 74.9)	52.4 (29.8, 74.3)	58.8 (32.9, 81.6)	71.4 (47.8, 88.7)	46.7 (21.3, 73.4)	57.9 (33.5, 79.7)
**positive 24 weeks post-boost**				*p=0.044*		
**(18-50 years)**	45.7 (28.8, 63.4)	40.6 (23.7, 59.4)	35.7 (18.6, 55.9)	56.3 (37.7, 73.6)	35.7 (18.6, 55.9)	69.2 (48.2, 85.7)
**(51-70 years)**	38.5 (13.9, 68.4)	28.6 (11.3, 52.2)	56.3 (29.9, 80.2)	25.0 (8.7, 49.1)	20.0 (4.3, 48.1)	38.9 (17.3, 64.3)
B/Wisconsin/1/2010						
**≥1:10 at baseline**						
**(18-50 years)**	39.5 (24.0, 56.6)	37.1 (21.5, 55.1)	21.9 (9.3, 40.0)	25.7 (12.5, 43.3)	29.0 (14.2, 48.0)	35.5 (19.2, 54.6)
**(51-70 years)**	23.1 (5.0, 53.8)	19.0 (5.4, 41.9)	17.6 (3.8, 43.4)	9.5 (1.2, 30.4)	18.8 (4.0, 45.6)	15.8 (3.4, 39.6)
**≥1:40 at baseline**						
**(18-50 years)**	26.3 (13.4, 43.1)	25.7 (12.5, 43.3)	18.8 (7.2, 36.4)	22.9 (10.4, 40.1)	25.8 (11.9, 44.6)	29.0 (14.2, 48.0)
**(51-70 years)**	15.4 (1.9, 45.4)	4.8 (0.1, 23.8)	5.9 (0.1, 28.7)	4.8 (0.1, 23.8)	12.5 (1.6, 38.3)	5.3 (0.1, 26.0)
**positive 3 weeks post prime**					*p=0.009*	
**(18-50 years)**	7.9 (1.7, 21.4)	2.9 (0.1, 14.9)	40.6 (23.7, 59.4)	60.0 (42.1, 76.1)	35.5 (19.2, 54.6)	54.8 (36.0, 72.7)
**(51-70 years)**	0.0 (0.0, 24.7)	0.0 (0.0, 16.1)	11.8 (1.5, 36.4)	47.6 (25.7, 70.2)	0.0 (0.0, 20.6)	52.6 (28.9, 75.6)
**positive 3 weeks post-boost**		*p=0.037*				
**(18-50 years)**	47.4 (31.0, 64.2)	44.1 (27.2, 62.1)	35.7 (18.6, 55.9)	64.7 (46.5, 80.3)	53.6 (33.9, 72.5)	50.0 (30.6, 69.4)

**(51-70 years)**	15.4 (1.9, 45.4)	14.3 (3.0, 36.3)	11.8 (1.5, 36.4)	42.9 (21.8, 66.0)	0.0 (0.0, 21.8)	42.1 (20.3, 66.5)
**positive 24 weeks post-boost**<br>**(18-50 years)**<br>**(51-70 years)**	*p=0.010*<br>37.1 (21.5, 55.1)<br>0.0 (0.0, 24.7)	*p=0.004*<br>31.3 (16.1, 50.0)<br>0.0 (0.0, 16.1)	 25.0 (10.7, 44.9)<br>12.5 (1.6, 38.3)	 31.3 (16.1, 50.0)<br>15.0 (3.2, 37.9)	*p=0.008*<br>35.7 (18.6, 55.9)<br>0.0 (0.0, 21.8)	 34.6 (17.2, 55.7)<br>22.2 (6.4, 47.6)
B/Texas/6/2011						
**≥1:10 at baseline**<br>**(18-50 years)**<br>**(51-70 years)**	 60.5 (43.4, 76.0)<br>41.7 (15.2, 72.3)	 51.4 (34.0, 68.6)<br>28.6 (11.3, 52.2)	 43.8 (26.4, 62.3)<br>17.6 (3.8, 43.4)	 44.1 (27.2, 62.1)<br>20.6 (8.7, 37.9)	 41.9 (24.5, 60.9)<br>25.0 (7.3, 52.4)	 38.7 (21.8, 57.8)<br>42.1 (20.3, 66.5)
**≥1:40 at baseline**<br>**(18-50 years)**<br>**(51-70 years)**	 28.9 (15.4, 45.9)<br>16.7 (2.1, 48.4)	 28.6 (14.6, 46.3)<br>4.8 (0.1, 23.8)	 21.9 (9.3, 40.0)<br>5.9 (0.1, 28.7)	 20.6 (8.7, 37.9)<br>9.5 (1.2, 30.4)	 25.8 (11.9, 44.6)<br>12.5 (1.6, 38.3)	 22.6 (9.6, 41.1)<br>15.8 (3.4, 39.6)
**positive 3 weeks post prime**<br>**(18-50 years)**<br>**(51-70 years)**	 5.3 (0.6, 17.7)<br>0.0 (0.0, 26.5)	 0.0 (0.0, 10.0)<br>0.0 (0.0, 16.1)	 34.4 (18.6, 53.2)<br>11.8 (1.5, 36.4)	 64.7 (46.5, 80.3)<br>47.6 (25.7, 70.2)	 32.3 (16.7, 51.4)<br>12.5 (1.6, 38.3)	 58.1 (39.1, 75.5)<br>38.9 (17.3, 64.3)
**positive 3 weeks post-boost**<br>**(18-50 years)**<br>**(51-70 years)**	*p=0.019*<br>57.9 (40.8, 73.7)<br>16.7 (2.1, 48.4)	*p=0.026*<br>61.8 (43.6, 77.8)<br>28.6 (11.3, 52.2)	 25.9 (11.1, 46.3)<br>17.6 (3.8, 43.4)	 45.5 (28.1, 63.6)<br>33.3 (14.6, 57.0)	*p=0.008*<br>39.3 (21.5, 59.4)<br>0.0 (0.0, 21.8)	 48.1 (28.7, 68.1)<br>33.3 (13.3, 59.0)
**positive 24 weeks post-boost**<br>**(18-50 years)**<br>**(51-70 years)**	 31.4 (16.9, 49.3)<br>8.3 (0.2, 38.5)	*p=0.002*<br>34.4 (18.6, 53.2)<br>0.0 (0.0, 16.1)	 11.1 (2.4, 29.2)<br>12.5 (1.6, 38.3)	 22.6 (9.6, 41.1)<br>5.0 (0.1, 24.9)	*p=0.008*<br>39.3 (21.5, 59.4)<br>0.0 (0.0, 21.8)	 34.6 (17.2, 55.7)<br>16.7 (3.6, 41.4)
A/Texas/50/2012 (H3N2)						
**≥1:10 at baseline**<br>**(18-50 years)**<br>**(51-70 years)**	 55.3 (38.3, 71.4)<br>84.6 (54.6, 98.1)	 74.3 (56.7, 87.5)<br>61.9 (38.4, 81.9)	 59.4 (40.6, 76.3)<br>52.9 (27.8, 77.0)	 60.0 (42.1, 76.1)<br>33.3 (14.6, 57.0)	 53.3 (34.3, 71.7)<br>68.8 (41.3, 89.0)	 58.1 (39.1, 75.5)<br>68.4 (43.3, 87.4)
**≥1:40 at baseline**<br>**(18-50 years)**<br>**(51-70 years)**	 50.0 (33.4, 66.6)<br>38.5 (13.9, 68.4)	 48.6 (31.4, 66.0)<br>33.3 (14.6, 57.0)	 46.9 (29.1, 65.3)<br>29.4 (10.3, 56.0)	 48.6 (31.4, 66.0)<br>28.6 (11.3, 52.2)	 43.3 (25.5, 62.6)<br>37.5 (15.2, 64.6)	 45.2 (27.3, 64.0)<br>47.4 (24.4, 71.1)
**positive 3 weeks post prime**<br>**(18-50 years)**<br>**(51-70 years)**	 5.3 (0.6, 17.7)<br>0.0 (0.0, 26.5)	 0.0 (0.0, 10.0)<br>0.0 (0.0, 16.1)	 34.4 (18.6, 53.2)<br>11.8 (1.5, 36.4)	 64.7 (46.5, 80.3)<br>47.6 (25.7, 70.2)	 32.3 (16.7, 51.4)<br>12.5 (1.6, 38.3)	 58.1 (39.1, 75.5)<br>38.9 (17.3, 64.3)
**positive 3 weeks post-boost**<br>**(18-50 years)**<br>**(51-70 years)**	*p=0.019*<br>57.9 (40.8, 73.7)<br>23.1 (5.0, 53.8)	*p=0.026*<br>38.2 (22.2, 56.4)<br>47.6 (25.7, 70.2)	 39.3 (21.5, 59.4)<br>41.2 (18.4, 67.1)	 61.8 (43.6, 77.8)<br>71.4 (47.8, 88.7)	*p=0.008*<br>35.7 (18.6, 55.9)<br>40.0 (16.3, 67.7)	 67.9 (47.6, 84.1)<br>36.8 (16.3, 61.6)
**positive 24 weeks post-boost**<br>**(18-50 years)**<br>**(51-70 years)**	*p=0.036*<br>42.9 (26.3, 60.6)<br>(7.7, 0.2, 36.0)	 28.1 (13.7, 46.7)<br>14.3 (3.0, 36.3)	 25.0 (10.7, 44.9)<br>37.5 (15.2, 64.6)	 46.9 (29.1, 65.3)<br>30.0 (11.9, 54.3)	 28.6 (13.2, 48.7)<br>13.3 (1.7, 40.5)	 46.2 (26.6, 66.6)<br>22.2 (6.4, 47.6)
B/Massachusetts/2/2012						
**≥1:10 at baseline**<br>**(18-50 years)**<br>**(51-70 years)**	 42.1 (26.3, 59.2)<br>23.1 (5.0, 53.8)	 37.1 (21.5, 55.1)<br>23.8 (8.2, 47.2)	 28.1 (13.7, 46.7)<br>11.8 (1.5, 36.4)	 48.6 (31.4, 66.0)<br>23.8 (8.2, 47.2)	 35.5 (19.2, 54.6)<br>18.8 (4.0, 45.6)	 29.0 (14.2, 48.0)<br>31.6 (12.6, 56.6)
**≥1:40 at baseline**<br>**(18-50 years)**<br>**(51-70 years)**	 21.1 (9.6, 37.7)<br>7.7 (0.2, 36.0)	 22.9 (10.4, 40.1)<br>0.0 (0.0, 16.1)	 21.9 (9.3, 40.0)<br>5.9 (0.1, 28.7)	 25.7 (12.5, 43.3)<br>14.3 (3.0, 36.3)	 22.6 (9.6, 41.1)<br>6.3 (0.2, 30.2)	 9.7 (2.0, 25.8)<br>5.3 (0.1, 26.0)
**positive 3 weeks post prime**<br>**(18-50 years)**<br>**(51-70 years)**	 2.6 (0.1, 13.8)<br>0.0 (0.0, 24.7)	 0.0 (0.0, 10.0)<br>0.0 (0.0, 16.1)	 28.1 (13.7, 46.7)<br>11.8 (1.5, 36.4)	 45.7 (28.8, 63.4)<br>33.3 (14.6, 57.0)	*p=0.019*<br>29.0 (14.2, 48.0)<br>0.0 (0.0, 20.6)	 61.3 (42.2, 78.2)<br>36.8 (16.3, 61.6)
**positive 3 weeks post-boost**<br>**(18-50 years)**	 39.5 (24.0, 56.6)	*p=0.001*<br>47.1 (29.8, 64.9)	 14.3 (4.0, 32.7)	 26.5 (12.9, 44.4)	*p=0.017*<br>32.1 (15.9, 52.4)	 35.7 (18.6, 55.9)

**(51-70 years)**	15.4 (1.9, 45.4)	4.8 (0.1, 23.8)	5.9 (0.1, 28.7)	23.8 (8.2, 47.2)	0.0 (0.0, 21.8)	15.8 (3.4, 39.6)
**positive 24 weeks post-boost**		*p=0.004*				
**(18-50 years)**	20.0 (8.4, 36.9)	31.3 (16.1, 50.0)	3.6 (0.1, 18.3)	18.8 (7.2, 36.4)	14.3 (4.0, 32.7)	15.4 (4.4, 34.9)
**(51-70 years)**	0.0 (0.0, 24.7)	0.0 (0.0, 16.1)	0.0 (0.0, 20.6)	5.0 (0.1, 24.9)	0.0 (0.0, 21.8)	5.6 (0.1, 27.3)

[a]Positive immune response defined as four-fold increase if reference titer (baseline or pre-boost) is ≥1:10, or ≥1:40 if reference titer is <1:10

[b]Number of subjects per group (n=) is based on the number of samples run at baseline for A/California/07/09

p values were determined by Fisher's exact test, indicate comparisons between the age subgroups at each time point, and are only displayed when p<0.05.
