# Supplementary material for: Safety and immunogenicity of investigational seasonal influenza hemagglutinin DNA vaccine followed by trivalent inactivated vaccine administered intradermally or intramuscularly in healthy adults: An open-label randomized phase 1 clinical trial
Source: PLoS One. 2019 Sep 18;14(9):e0222178. doi: 10.1371/journal.pone.0222178 (PMC6750650; doi:10.1371/journal.pone.0222178)
Supplement: S2 Fig — (A) GMT and (B) seroconversion rates are shown for antibody responses measured by HAI at 3 weeks post boost for all 2012/13 and 2013/14 vaccine strains, with error bars indicating the 95% CI. For each vaccination regimen results are displayed for both the ID (blue) and IM (red) routes. Comparisons were made between administration routes for each vaccine regimen. Displayed p values for seroconversion rates were calculated based on Fisher’s Exact test, while GMT comparisons were based on pairwise T-test. (PDF) [file pone.0222178.s010.pdf]

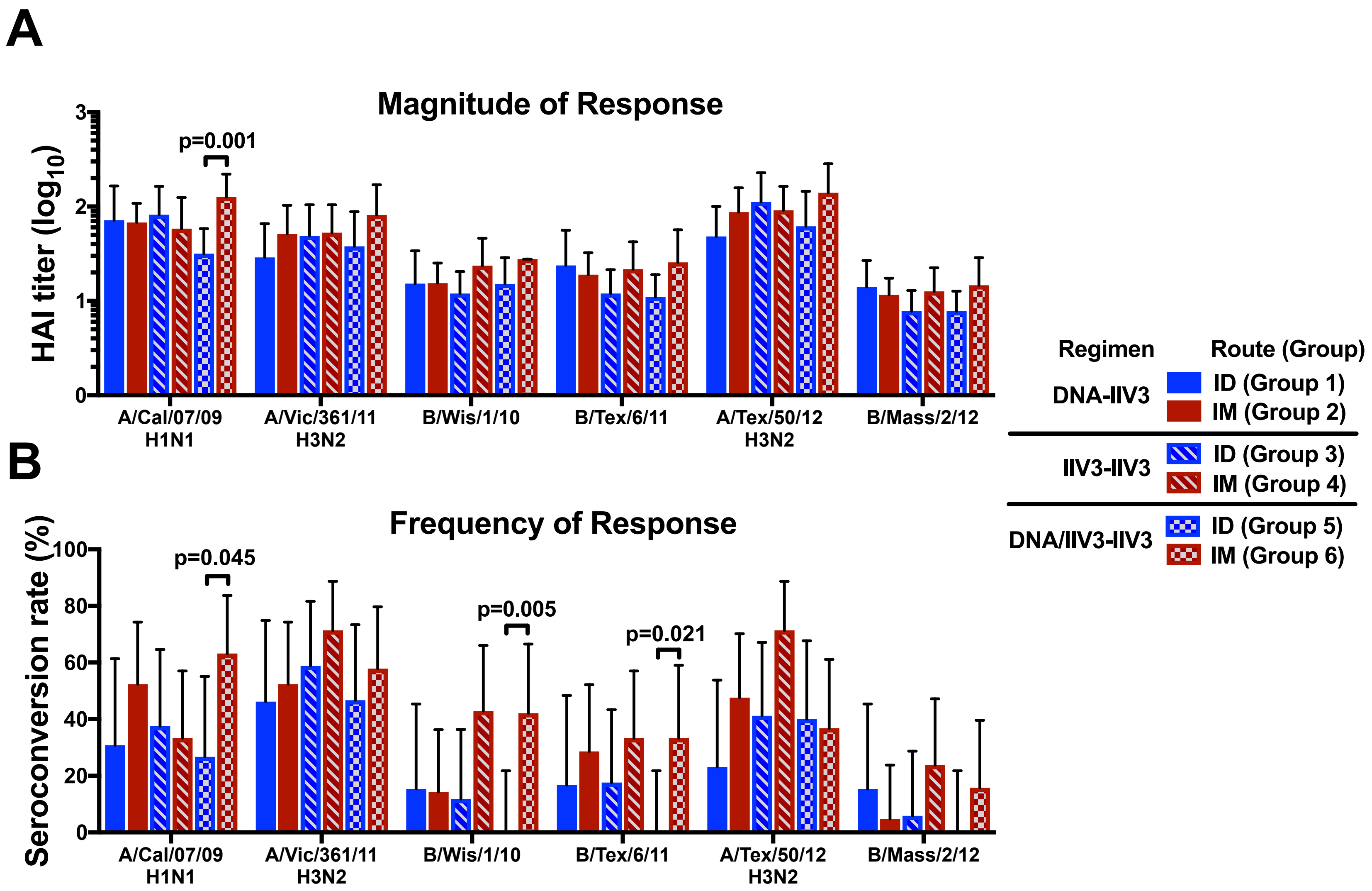

S2 Fig. Route comparison of magnitude and frequency of antibody response in the older adult subgroups post boost.
